# Proteomic analysis of extracellular vesicles derived from canine mammary tumour cell lines identifies protein signatures specific for disease state

**DOI:** 10.1186/s12917-024-04331-1

**Published:** 2024-10-26

**Authors:** Tania Gutierrez-Riquelme, Isabel Karkossa, Kristin Schubert, Gudrun Liebscher, Eva-Maria Packeiser, Ingo Nolte, Martin von Bergen, Hugo Murua Escobar, Matias Aguilera-Rojas, Ralf Einspanier, Torsten Stein

**Affiliations:** 1 Institute of Veterinary Biochemistry, Department of Veterinary Medicine, Freie Universität Berlin, 14163 Berlin, Germany; 2 Department of Molecular Systems Biology, Helmholtz Centre of Environmental Research GmbH – UFZ, 04318 Leipzig, Germany; 3 Reproductive Unit, Clinic for Small Animals, University of Veterinary Medicine Hannover, Foundation, 30559 Hannover, Germany; 4 Department of Small Animal Medicine and Surgery, University of Veterinary Medicine Hannover, Foundation, 30559 Hannover, Germany; 5 Department of Internal Medicine, Medical Clinic III, Clinic for Hematology, Oncology and Palliative Care, University Medical Center Rostock, Ernst-Heydemann-Strasse 6, 18057 Rostock, Germany; 6 Department of Medicine, Health and Medical University, 14471 Potsdam, Germany

**Keywords:** Canine mammary tumour, Cell culture, Extracellular vesicles, Proteomics, Size exclusion chromatography, WGCNA, Biglycan

## Abstract

**Background:**

Canine mammary tumours (CMT) are among the most common types of tumours in female dogs. Diagnosis currently requires invasive tissue biopsies and histological analysis. Tumour cells shed extracellular vesicles (EVs) containing RNAs and proteins with potential for liquid biopsy diagnostics. We aimed to identify CMT subtype-specific proteome profiles by comparing the proteomes of EVs isolated from epithelial cell lines derived from morphologically normal canine mammary tissue, adenomas, and carcinomas.

**Methods:**

Whole-cell protein lysates (WCLs) and EV-lysates were obtained from five canine mammary cell lines: MTH53A (non-neoplastic); ZMTH3 (adenoma); MTH52C (simple carcinoma); 1305, DT1406TB (complex carcinoma); and their proteins identified by LC-MS/MS analyses. Gene Ontology analysis was performed on differentially abundant proteins from each group to identify up- and down-regulated biological processes. To establish CMT subtype-specific proteomic profiles, weighted gene correlation network analysis (WGCNA) was carried out.

**Results:**

WCL and EVs displayed distinct protein abundance signatures while still showing the same increase in adhesion, migration, and motility-related proteins in carcinoma-derived cell lines, and of RNA processing and RNA splicing factors in the adenoma cell line. WGCNA identified CMT stage-specific co-abundant EV proteins, allowing the identification of adenoma and carcinoma EV signatures not seen in WCLs.

**Conclusions:**

EVs from CMT cell lines exhibit distinct protein profiles reflecting malignancy state, allowing us to identify potential biomarkers for canine mammary carcinomas, such as biglycan. Our dataset could therefore potentially serve as a basis for the development of a less invasive clinical diagnostic tool for the characterisation of CMT.

**Supplementary Information:**

The online version contains supplementary material available at 10.1186/s12917-024-04331-1.

## Background

Canine mammary tumours (CMT) are the most common tumours in intact female dogs. Approximately 50% of canine mammary neoplasia cases are malignant and the mortality rate is high if left untreated [1]. CMTs have many similar biological features to human breast cancer, including presentation [2], histopathological features [3], biological behaviour [4] and metastatic patterns [5]. Accordingly, dogs are considered good models for human disease. However, there are also distinct differences between both species mostly associated with morphology and clinical presentation. In dogs, carcinomas are classified as simple, when consisting of luminal epithelial cells or myoepithelial components; complex, when made up of both luminal epithelial and myoepithelial components; or mixed, if they contain both epithelial and/or myoepithelial components along with mesenchymal cells [6].

For the diagnosis and classification of CMT, conventional biopsy with histopathology remains the gold standard [7]. However, the morphological heterogeneity of the tumours and the presence of different cell types make an accurate classification difficult [7]. Moreover, the poor prognosis in dogs with large, visible tumours reflects the advanced stage of the pathology, further emphasizing the importance of early detection due to the high malignancy rate [8]. An accurate biomarker for CMT diagnosis with malignant prediction could improve outcomes through intervention at an earlier stage of disease, as well as assess response to treatment, presence of tumour progression, or future prognosis, especially since CMTs represent a very diverse group of tumours and therefore different approaches of treatment will be necessary [9].

In recent years, the study of extracellular vesicles (EVs) has caught the attention of researchers as an emerging and promising tool for disease diagnosis and prognosis [10]. EVs are defined as a heterogeneous group of small lipid-bilayered particles which are constantly released from cells into the extracellular environment. These vesicles transport biological molecules such as lipids, proteins and nucleic acids, playing a key role in cellular communication in multiple physiological and pathological processes [11]. Several studies have shown that EVs play a direct role in the crosstalk between tumour cells and stromal cells, contributing to tumour growth, enhancement of tumour cell invasion, and potential microenvironmental remodelling, leading to tumour cell metastasis [12, 13]. Moreover, EV cargo is highly stable over extended periods of time due to the EV’s lipid bilayer, which prevents degradation by extracellular proteases and nucleases, thereby enhancing their potential for biomarker discovery and clinical diagnosis [14].

In this study, we performed a comprehensive proteome analysis of CMT cell line-derived EVs and paired WCLs from a benign adenoma, as well as one simple and two complex carcinoma-derived cell lines compared to a cell line established from normal mammary tissue. Our aim was to identify molecular markers for disease stage and to provide useful insights into the molecular and biological processes that may be induced by EVs released from tumour cells. By using weighted gene correlation network analysis (WGCNA), a powerful systems biology method described by Langfelder and Horvath, (2008) to identify correlation patterns among genes/proteins [15], we identified co-abundant proteins, pathways relevant to our dataset, as well as key proteins in the proteomics data derived from different WCLs and EVs that may serve as potential biomarkers or therapeutic targets.

## Methods

### Canine mammary cell lines

The following canine mammary cell lines were used: one non-neoplastic cell line (MTH53A, used as a healthy control), one simple adenoma cell line (ZMTH3), one simple carcinoma cell line (MTH52C) and two complex carcinoma cell lines (1305, DT1406TB). Histological classification of the tissues from which the cell lines have been derived and established was performed by the Department of Pathology of the Stiftung Tierärztliche Hochschule Hannover, Germany [16], according to the proposed classification of canine mammary tumours [17]. Cells were cultured in DMEM/F12 medium (1:1) + GlutaMAX™-l, supplemented with 10% foetal bovine serum (FBS) superior (both from Thermo Fisher Scientific, Paisley, UK), 1% penicillin/streptomycin (P/S) (Life Technologies, Inc., New York, USA) and 1% sodium pyruvate (Sigma-Aldrich, Taufkirchen, Germany). All cells were grown in a humidified incubator at 37 °C and 5% CO_2_.

### Sample preparation for EV isolation

Cells were cultured in T-175 flasks at 90% confluence, rinsed twice with phosphate-buffered saline (PBS) and incubated at 37 °C and 5% CO_2_ for 48 h in serum-free medium (DMEM/F12) containing 1% pyruvate and 1% P/S. 180 ml of conditioned media was harvested per cell line and centrifuged for 30 min at 2000 x g to remove cells and cell debris. Supernatant was collected and stored at 4 °C until further processing.

### EV isolation

The EV isolation protocol was designed and optimised as previously described [18–21], using two replicates per cell line. To initially concentrate EVs, ultrafiltration devices with a cut-off of 50 kDa (Thermo Fisher Scientific) were loaded with conditioned media from each cell line, centrifuged at 3500 x g at 4 °C until the volume was reduced to 300 µl. To isolate EVs, 150 µl of concentrated conditioned culture medium was loaded onto a size-exclusion column (SEC) (Cell Guidance Systems Ltd, Cambridge, UK), and twenty-four 200 µl fractions were eluted by gravity with PBS. This procedure was repeated twice for each cell line and fractions 5–10 were collected and pooled.

### Nanoparticle tracking analysis (NTA)

Concentration and size of EVs were analysed using Nanosight NS500 and Nanosight LM14 devices (both from Malvern, Worcestershire, UK). Samples were processed in duplicate and diluted 100-fold in filtered PBS (0.22 μm pore PVDF filter). All analyses were performed at 25 °C and three videos of 30 to 60 s were recorded for each sample. Data were processed and analysed using the NTA 3.2 software (Malvern).

### Protein extraction

To prepare WCL from each cell line, cells were cultured in 6-well plates until near-confluency. Cells were rinsed twice with PBS and incubated in serum-free medium for 48 h to mirror EV isolation conditions. Cells were lysed in 200 µl RIPA buffer with protease inhibitors for 20 min on ice, cell lysates were scraped off and passed several times through a 25-gauge needle using a 1 ml syringe. Samples were centrifuged at 13,000 x g at 4 °C for 10 min and supernatant was stored at -20 °C until further use. Protein quantification of all samples was determined by Pierce BCA assay (Thermo Fisher Scientific) according to the manufacturer’s instructions.

For protein isolation from EVs, selected SEC fractions were concentrated with a 2 kDa cut-off centrifugation concentrator device (Thermo Fisher Scientific) to approximately 60 µl at 13,000 x g at 4 °C. Samples were lysed in 60 µl 2x RIPA buffer supplemented with protease inhibitors (#5871 S, Cell Signaling Technology, Leiden, The Netherlands) and centrifuged as described for WCLs.

### Immunofluorescence

Sterile 13-mm diameter coverslips (Sarstedt) were placed in a standard 24-well plate, and cell lines were seeded and cultured for 24 h, followed by an additional 48 h in serum-free medium to mimic the conditions used in protein extraction. The cells were washed with cold PBS and fixed with methanol on ice for 15 min. A quenching step with 10 mM glycine was performed for 15 min at room temperature. Coverslips were blocked with 1% BSA/PBST for 30 min at room temperature, followed by incubation in a 50 µL drop of primary antibody dilution in 1% BSA/PBST for 1 h at room temperature (rabbit anti-biglycan (BGN) antibody (16409-1-AP, 1:500, Proteintech, Manchester, UK). Coverslips were washed twice with PBS and incubated with a 50 µL drop of secondary antibody for 1 h at room temperature in the dark (goat-anti rabbit IgG Dylight 488 (1:1000)). The samples were then washed three times with PBS, counterstained and mounted using Prolong Glass with Hoechst (Life Technologies, Willow Creek, Oregon, USA) on a glass slide and cured for 24 h at room temperature in the dark. Images were acquired under identical settings using a Leica DMI6000B inverted microscope and Leica LAS-X software (Leica, Wetzlar, Germany).

### Western blotting

10 µg of EV proteins were separated by 12% Bis/Tris SDS-PAGE and transferred to a nitrocellulose membrane via semidry blot. The membrane was blocked for 30 min on 3% milk/TBST. Primary antibodies used were rabbit anti-BGN antibody (1:500, Proteintech), goat anti-CD63 antibody (1:1000, Antibodies-online GmbH, Aachen, Germany) and mouse anti-CD9 antibody (1:1000, Thermo Fisher Scientific). For BGN detection, a pre-treatment of the samples was necessary to deglycosylate the chondroitin sulphate chains of the proteoglycan, using 0.5 µg of chondroitinase ABC (Bio-Techne, Massachusetts, USA) per 10 µg of protein and incubation at 37 °C for 16 h prior to western blotting. Secondary antibodies were as follows: donkey anti-rabbit (NA934), sheep anti-mouse (NA931) horseradish peroxidase-conjugated antibodies (1:10,000, GE Healthcare, Buckinghamshire, UK), and donkey anti-goat (sc-2020) horseradish peroxidase-conjugated antibody (1:10.000, Santa Cruz Biotechnology, Inc., Heidelberg, Germany). A Prime ECL western blotting detection kit (Cytiva, Little Chalfont, Buckinghamshire, UK) was used and imaged using a Fusion imaging system (Vilber Lourmat, Marne-la-Vallée, France).

### Proteomics using liquid chromatography with tandem mass spectrometry (LC-MS/MS)

Mass spectrometry was performed as previously described [22, 23] using Sera-Mag SP3 beads (GE Healthcare, Solingen, Germany) and label-free protein quantification. Samples were analysed from two independent isolates, each in duplicate per cell line. 2 µL (20 µg) SpeedBeads magnetic carboxylate-modified particles per sample were prepared by washing twice with 200 µl water. Each 15 µl protein sample was brought to a volume of 50 µl with 100 mM triethylammonium bicarbonate (TEAB). Protein sidechains were reduced by addition of 5 µl 200 mM tris(2-carboxyethyl) phosphine in 100 mM TEAB and incubation for 1 h at 55 °C. The alkylation was performed by adding 5 µl 375 mM iodoacetamide in 100 mM TEAB and incubation at room temperature for 30 min in darkness. 70 µl acetonitrile (ACN) was added and incubated with the prepared beads for 8 min at room temperature. Tubes were placed on a magnetic rack for 2 min, supernatant discarded, and beads washed twice with 200 µl ethanol (70% in water, v/v) and once with ACN. Trypsin (1:50 enzyme to protein ratio, diluted in 100 mM TEAB) (Promega Corporation, Madison, USA) was added to each sample for overnight enzymatic cleavage (16 h) at 37 °C. Thereafter, 200 µl ACN was added to the peptides. After 8 min incubation, samples were incubated for 2 min on a magnetic rack, supernatant was discarded, and beads were washed with 200 µl ACN. Peptides were eluted by addition of water with dimethylsulfoxide (2%, v/v) and sonication for 1 min. After 2 min incubation on a magnetic rack, supernatants were collected, vacuum-dried and reconstituted in water with formic acid (0.1%, v/v).

Obtained peptides were separated using an Ultimate 3000 nano ultra-performance liquid chromatography system (Thermo Fisher Scientific). Peptides were first trapped on an Acclaim PepMap 100 C18 column (Acclaim PepMap 100 C18, nanoViper, 2 μm, 75 μm x 5 cm) (Thermo Fisher Scientific), and subsequently separated on an analytical reverse-phase column Acclaim PepMap 100 C18 (Acclaim PepMap 100 C18, nanoViper, 3 μm, 75 μm x 25 cm) (Thermo Fisher Scientific). The separated peptides were injected into a Q Exactive HF Orbitrap mass spectrometer (Thermo Fisher Scientific) equipped with a TriVersa NanoMate system (Advion, Ithaca, New York, USA). Samples were acquired using parameters described previously [22]. Raw data were processed using Proteome Discoverer 2.5 (Thermo Fisher Scientific). The database search was performed against the UniprotKB reference proteome of *Canis lupus familiaris* (from 3rd February 2023). As parameters, oxidation of methionine and acetylation of proteins N-termini were set as variable modifications and carbamidomethylation of cysteines as fixed modification. Two missed tryptic cleavages were allowed and proteins with at least two identified peptides and one unique peptide were considered identified. Proteins were quantified by summing the intensities of all unique peptides.

### **Data processing and statistical analysis**

For statistical analysis and visualisation, R v 3.6.1 with the workflow described by the package proteomicsr was used [24], which applies the following packages: mixOmics [25], corrplot [26], limma [27], PerformanceAnalytics [28], dendsort [29], ComplexHeatmap [30], plyr [31], reshape2 [32], xlsx [29], DEP [33], ggsci [34], circlize [35], calibrate [36], ggplot2 [37], readxls [38], qpcR [39], splitstackshape [40], tidyr [41], ggh4x [42], dendextend [43] and Tmisc [44]. Data were log2-transformed, median-normalized, variance-stabilized, and filtered for proteins quantified in at least two of four replicates. Imputation of noise-like values was applied for conditions with no quantification in any of the available replicates. To assess significant changes, a Student’s t-test with Benjamini & Hochberg adjustment for multiple testing was performed, and proteins with adjusted p-values ≤ 0.05 were considered significantly affected.

### WGCNA analysis

Normalised intensity data were subjected to Weighted Gene Correlation Analysis (WGCNA) [15, 45] using R v.3.6.1. Modules were created applying the default parameters with the following exceptions: soft power threshold: 21, minimum module size: 50, maximum module size: 200, deep split: 0, merge cut height: 0.4. Modules were correlated to traits using Pearson correlation. For module-trait combinations of particular interest, potential key drivers were determined based on their absolute module membership (MM) and absolute trait significance (TS) ≥ 0.75. Alternatively, the top 20 candidates with highest summed absolute MM and TS were evaluated. Log2(FC)s and adjusted p-values of the identified potential key drivers were used for visualisation.

### Gene ontology analysis

Differentially abundant proteins were analysed using gene ontology (GO) functional annotations using ShinyGO 0.80 database [46]. False discovery rate (FDR) cut-off smaller 0.05 was considered significant, and enriched pathways were sorted by fold enrichment (F.E.) values. Venn diagrams were generated for multiple comparisons using the InteractiVenn tool [47].

## Results

### Protein identification by LC-MS/MS

**Fig. 1:**
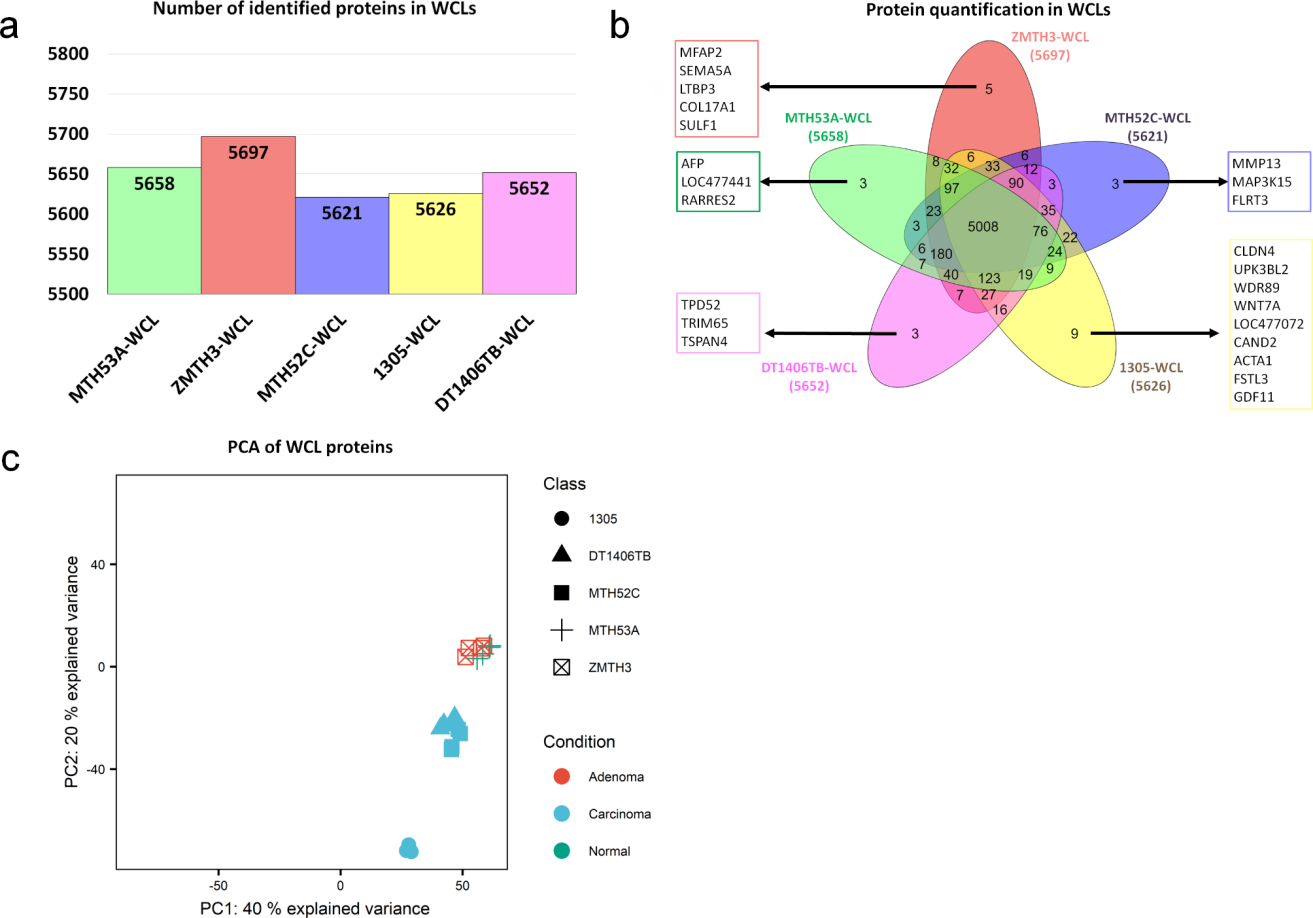
Mass spectrometry-based profiling of whole-cell proteomes. **(a)** Protein groups identified in each WCL-derived cell line. **(b)** Overlap of protein intensities quantified in each WCL-derived cell line and uniquely detected proteins in each cell line. **(c)** Principal component analysis of WCL proteins of healthy control and CMT cell lines

To identify proteins that are specifically associated with normal, adenomatous or cancerous cells, whole-cell lysates (WCLs) from cell lines established from a morphologically normal canine mammary gland (MTH53A), a mammary adenoma (ZMTH3), a simple carcinoma (MTH52C) and two complex carcinomas (1305, DT1406TB) (Additional file 1) were first compared. In total, 5936 proteins were identified by LC-MS/MS, ranging in number between 5621 and 5697 for each WCL sample (Fig. 1a). The vast majority of detected proteins (5006 proteins) was present in all cell lines and only very few cell line-specific proteins (between 3 and 9 per cell line) were detected (Fig. 1b).

Principal component analysis (PCA) of these WCLs (Fig. 1c) revealed that the four technical replicates from two independent WCLs per cell line (biological replicates) clustered very closely together, showing good reproducibility. Proteomes of the WCLs of the non-neoplastic cell line MTH53A (healthy control) and the adenoma ZMTH3 clustered together (Fig. 1c), while those from the simple carcinoma MTH52C and complex carcinomas 1305 and DT1406TB formed two separate clusters. Surprisingly, the two complex carcinoma cell lines did not cluster together but instead DT1406TB clustered with to the simple carcinoma MTH52C, while 1305 cells formed a separate cluster.

### Cell line specific WCL proteomic profiles allow to distinguish CMT subtypes

**Fig. 2:**
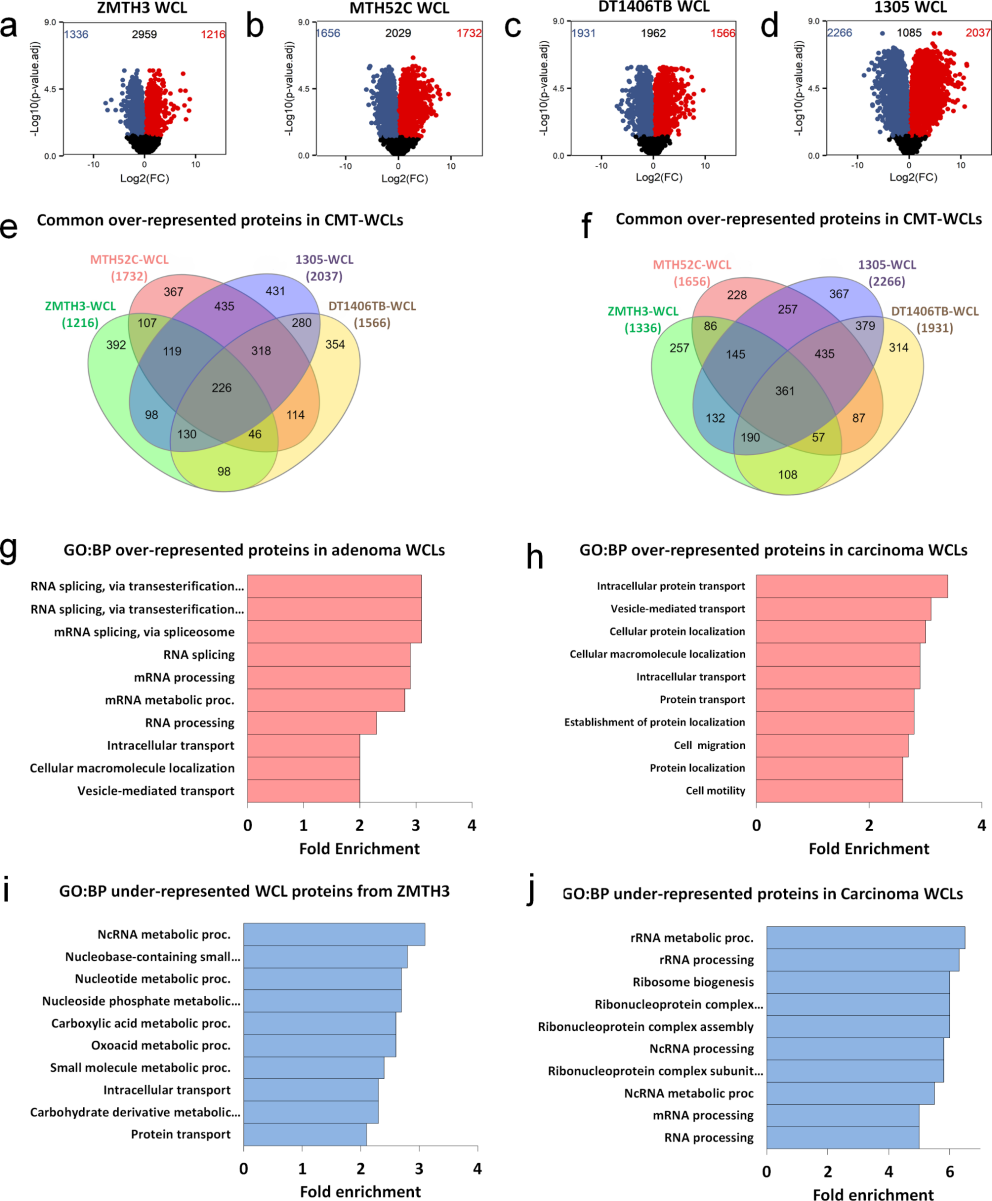
Differential analysis in WCL protein abundance levels of CMTs when compared to healthy control MTH53A. Volcano plots of **(a)** adenoma ZMTH3, **(b)** Volcano plot of simple carcinoma MTH52C, **(c)** complex carcinoma DT1406TB, and **(d)** complex carcinoma 1305, when compared to the healthy control. Red dots in the top right area were over-represented in the CMT cell line relative to MTH53A. Blue dots in the top left area were under-represented in CMT cell line relative to MTH53A. Black dots below the dashed line represent proteins with no statistical difference (*p* > 0.05). **(e)** Venn diagram showing the overlap between the WCL proteins over-represented (*p* < 0.05) in all the CMT cell lines. **(f)** Gene ontology of over-represented ZMTH3 WCL proteins enriched in biological process pathways (GO: BP) expressed as fold enrichment scores (F.E.), considering a false discovery rate (FDR) < 0.05. **(g)** Gene ontology of common over-represented WCL proteins of carcinoma cell lines enriched in biological process pathways (GO: BP). (h) Venn diagram showing the overlap between the WCL proteins under-represented (*p* < 0.05) in all the CMT cell lines. **(i)** Gene ontology of under-represented ZMTH3 WCL proteins enriched in biological process pathways (GO: BP). **(j)** Gene ontology of common under-represented WCL proteins of carcinoma cell lines enriched in biological process pathways (GO: BP)

Despite the low variance in the PCA, the WCL proteome of the adenoma ZMTH3 showed significant differences in protein abundance when directly compared against the healthy control MTH53A, with 2552 differentially abundant proteins (adjusted *p* ≤ 0.05) (Fig. 2a). As expected, WCLs of the carcinoma cell lines showed an even larger difference compared to the healthy control, with between 3388 and 4303 differentially abundant proteins (Fig. 2b-d).

Most over-represented proteins in the adenoma cell line ZMTH3 were associated with RNA-binding and protein expression (EIF1AX, CSRP1, PPIC) (Table 1), suggesting enhanced protein biosynthesis activity. Likewise, top under-represented proteins included those associated with cell adhesion (SERPINB8, DPT), signal transduction (CAPS, DOCK4) and metabolism regulation and homeostasis (CYP39A1, CKB), suggesting a change in adhesiveness together with a dysregulation of transduction pathways to promote cell growth and proliferation.

**Table 1:** Top 10 differentially over- and under-represented proteins identified in adenoma WCLs vs. healthy control

Over-represented proteins			
**Accession number**	**Protein**	**Log2FC**	**Adj. P-value**
A0A8I3QIQ3	EIF1AX	8.865	1.72E-04
A0A8I3NCP2	ACSF2	8.733	1.04E-03
A0A8I3NAS7	OCIAD2	8.120	3.99E-03
A0A8I3NH85	TNS3	8.029	4.57E-05
A0A8I3PWS9	LOC102153069	7.829	4.56E-04
A0A8P0PEA0	EBP	7.563	3.15E-06
A0A8I3P342	ISG15	7.047	4.40E-05
H6VX52	FAM83H	6.540	7.28E-04
A0A8I3N5K4	PPIC	6.353	6.61E-04
A0A8I3P8T5	CSRP1	5.776	4.89E-04
**Under-represented proteins**			
**Accession number**	**Protein**	**Log2FC**	**Adj. P-value**
A0A8I3NQ76	CYP39A1	-7.634	3.16E-04
P10463	CAPS	-7.247	9.38E-04
A0A8P0PP63	CNNM2	-6.508	1.89E-04
A0A8P0S626	SERPINB8	-5.666	9.38E-04
A0A8I3RS96	DPT	-4.693	5.70E-03
A0A8I3SCL9	DOCK4	-4.548	3.86E-04
A0A8I3S958	LOC478277	-4.450	9.68E-04
A0A8P0NPZ9	PLEKHA2	-4.354	6.81E-05
A0A8I3NKV1	TXNIP	-4.298	4.28E-04
P05124	CKB	-4.284	8.63E-06

Among the most over-represented proteins in the WCLs of carcinoma cell lines, proteins associated with actin cytoskeleton-rearrangement (TPM4, MYLK, PTK2B) as well as cell adhesion and migration (MDK, EPCAM) were detected (Additional file 2), thus indicating increased migratory and adhesion activity in the carcinoma cell lines. Surprisingly, several of the most under-represented proteins in the carcinoma WCLs included proteins involved in cell cycle regulation (CDK2, CDC45) and transcription regulation (SMARCA4, CHAF1B) (Additional file 2), indicating a dysregulation of cell growth and gene expression in carcinoma cells (for associations of cancer studies with proteins listed in Table 1 and Additional file 2, refer to Additional file 3).

### Mammary tumour cell lines from dogs share proteins associated with cancer progression

Evaluating the similarities of significantly over-represented proteins in the adenoma and carcinoma cell lines (Fig. 2e), 226 proteins were identified, of which the most over-represented included proteins involved in signal transduction (NUCB1, ZYK) and apoptosis (RIPK3, CASP8) (Table 2). Interestingly, the RNA-binding protein CSRP1 was one of the most abundant proteins for all CMTs, which has been linked to human breast cancer for its role in alteration of gene regulation, cell growth and differentiation [48]. Moreover, 318 over-represented proteins were common in the three carcinoma cell lines, including SERPINB5 among the most abundant proteins (Table 3), which is expressed in highly aggressive human basal-like breast cancers [49].

Additionally, 361 proteins were commonly under-represented in both adenoma and carcinoma cell lines (Fig. 2f), which were mainly associated with DNA replication, transcriptional activation and cell cycle regulation (NOC3L, TRIT1, CDK2, USP19) (Table 2); whereas the carcinoma cell lines shared 435 under-represented proteins, including splicing and chromatin regulator proteins (RTCB, SMARCA4) (Table 3, Additional file 2).

**Table 2:** Top 20 differentially common over-represented WCL proteins vs. healthy control in CMT cell lines

Common over-represented proteins						
**Accession number**	**Protein name**	**Protein abbreviation**	**ZMTH3 Log2FC**	**MTH52C** **Log2FC**	**1305** **Log2FC**	**DT1406TB** **Log2FC**
A0A8I3PIK8	Alpha-2-HS-glycoprotein	AHSG	3.875	7.537	7.181	5.387
A0A8I3NGU2	Folate_rec domain-containing protein	LOC476816	5.108	6.698	5.571	6.387
A0A8I3P8T5	Cysteine and glycine rich protein 1	CSRP1	5.776	2.430	8.488	6.356
A0A8I3N5K4	Peptidyl-prolyl cis-trans isomerase	PPIC	6.353	2.194	6.291	5.153
A0A8I3P375	Hook microtubule tethering protein 3	HOOK3	3.798	5.981	5.837	4.102
A0A8I3Q2T3	Reticulocalbin 1	RCN1	2.783	4.697	7.752	3.734
A0A8I3PW67	DLG associated protein 4	DLGAP4	1.708	4.136	6.593	4.383
Q38JA9	Caspase 8	CASP8	5.210	4.122	4.978	2.240
A0A8I3RP80	Nucleobindin-1	NUCB1	1.601	6.004	5.651	3.035
A0A8I3N5W9	Protein kinase domain-containing protein	RIPK3	3.977	3.259	4.929	3.830
A0A8I3MQM8	Prosaposin	PSAP	1.604	4.820	6.394	2.912
A0A8I3NTX2	Mitochondrial antiviral signaling protein	MAVS	2.310	4.379	6.169	2.631
A0A8I3P225	GLOBIN domain-containing protein	LOC609402	1.814	5.072	6.257	1.981
A0A8P0N4U0	Heme oxygenase	HMOX1	2.849	4.484	4.044	3.503
A0A8P0TML4	Ring finger protein 214	RNF214	2.850	5.212	3.562	3.099
A0A8I3MYL2	Mitochondrial trans-2-enoyl-CoA reductase	MECR	1.343	2.349	7.828	3.124
A0A8I3S1U2	Methyltransferase like 7 A	METTL7A	3.382	1.674	4.967	4.326
A0A8I3NUI7	Coatomer subunit zeta	COPZ2	2.575	2.756	4.486	4.503
A0A8I3MCD6	GLOBIN domain-containing protein	HBQ1	2.048	4.828	5.210	1.888
A0A8I3NVB0	Zyxin	ZYX	1.263	3.905	5.032	3.443
**Common under-represented proteins**						
**Accession number**	**Protein name**	**Protein abbreviation**	**ZMTH3** **Log2FC**	**MTH52C** **Log2FC**	**1305** **Log2FC**	**DT1406TB** **Log2FC**
A0A8I3NQ76	Cytochrome P450 family 39 subfamily A member 1	CYP39A1	-7.634	-5.342	-7.234	-4.981
A0A8I3NB48	Stonin-2	STON2	-3.664	-4.148	-3.505	-2.718
A0A8I3RWC5	Pleckstrin homology and RhoGEF domain containing G4	PLEKHG4	-3.007	-5.073	-5.241	-0.623
A0A8I3RS96	Dermatopontin	DPT	-4.693	-3.372	-4.739	-1.009
A0A8I3PI43	Nucleolar complex protein 3 homolog	NOC3L	-0.736	-3.834	-5.105	-4.048
A0A8I3Q982	Interferon related developmental regulator 1	IFRD1	-2.863	-1.472	-5.130	-4.098
A0A8I3NKM0	tRNA dimethylallyltransferase	TRIT1	-1.911	-2.817	-4.625	-2.501
A0A8I3Q4 × 2	Ubiquitin specific peptidase 19	USP19	-1.888	-2.480	-4.484	-2.824
A0A8I3PIR4	Fibroblast growth factor 7	GALK2	-3.957	-2.692	-2.801	-2.029
A0A8I3N128	Cyclin dependent kinase 2	CDK2	-0.388	-0.379	-7.813	-2.540
A0A8I3RUT0	rRNA adenine N(6)-methyltransferase	DIMT1	-1.667	-2.422	-5.646	-1.383
A0A8I3NJG0	TatD DNase domain containing 1	TATDN1	-2.207	-1.452	-4.510	-2.850
A0A8I3P8U7	Cms1 ribosomal small subunit homolog	CMSS1	-0.995	-2.899	-4.866	-2.214
A0A8I3NFP5	Neurobeachin like 2	NBEAL2	-2.744	-3.091	-3.938	-1.134
A0A8I3PB80	Hyaluronidase 3	HYAL3	-2.310	-2.229	-4.172	-2.080
A0A8I3PRT9	Inosine-5’-monophosphate dehydrogenase	IMPDH1	-3.233	-1.501	-4.005	-1.938
A0A8I3Q7R1	Inosine-5’-monophosphate dehydrogenase	IMPDH2	-2.070	-0.621	-4.916	-2.830
A0A8P0NPZ9	Pleckstrin homology domain containing A2	PLEKHA2	-4.354	-1.288	-2.812	-1.972
A0A8I3S0T5	CCAAT enhancer binding protein zeta	CEBPZ	-1.140	-4.729	-3.102	-1.406
A0A8P0NM37	ACD shelterin complex subunit and telomerase recruitment factor	ACD	-1.818	-1.722	-4.163	-2.623

**Table 3:** Top 20 differentially common over-represented WCL proteins vs. healthy control in the carcinoma cell lines

Common over-represented proteins					
**Accession number**	**Protein name**	**Protein abbreviation**	**MTH52C** **Log2FC**	**1305** **Log2FC**	**DT1406TB** **Log2FC**
A0A8I3MKP5	Serpin B5	SERPINB5	5.987	11.184	4.273
A0A8I3RRG1	Heat shock protein family B (small) member 6	HSPB6	6.269	9.503	4.919
A0A8I3Q497	RAB8A, member RAS onco family	TPM4	7.749	8.158	1.989
A0A8P0P7T7	Transforming acidic coiled-coil containing protein 1	TACC1	6.187	6.939	4.416
A0A8I3N4I3	Trophoblast glycoprotein	TPBG	4.280	5.255	7.631
A0A8I3Q6P3	RAB8A, member RAS onco family	TPM4	7.882	6.517	2.211
A0A8I3P542	fatty acid amide hydrolase	FAAH	3.651	7.188	5.457
A0A8I3S7B1	Bridging integrator 1	BIN1	5.290	6.640	3.024
A0A8I3PMV7	CutA divalent cation tolerance homolog	CUTA	5.372	7.128	2.272
A0A8P0NGG9	Galectin	LGALS3	1.521	7.307	5.419
A0A8I3NMV9	PBX homeobox interacting protein 1	PBXIP1	4.881	5.810	3.389
A0A8I3MUR7	DAB adaptor protein 2	DAB2	4.991	5.599	3.370
A0A8I3RR31	Myosin regulatory light chain 12B	MYL12B	2.191	6.458	5.301
A0A8I3NML2	Clathrin light chain	CLTB	3.828	7.208	2.910
A0A8I3MJ17	Abl interactor 1	ABI1	5.757	6.161	1.656
A0A8I3NZF1	Acylphosphatase	ACYP1	3.241	6.659	3.557
A0A8P0SCK9	IF rod domain-containing protein	KRT86	3.257	4.057	5.955
A0A8P0N5R9	Glycoprotein nmb	GPNMB	1.419	7.333	4.212
A0A8P0P6 × 6	Cadherin-2	CDH2	1.605	5.693	5.647
A0A8I3P9R4	Ectonucleoside triphosphate diphosphohydrolase 3	ENTPD3	3.408	5.338	4.067
**Common under-represented proteins**					
**Accession number**	**Protein name**	**Protein abbreviation**	**MTH52C** **Log2FC**	**1305** **Log2FC**	**DT1406TB** **Log2FC**
A0A8I3PDZ7	RNA-splicing ligase RtcB homolog	RTCB	-6.061	-5.268	-6.013
A0A8I3P3E3	Transglutaminase 3	TGM3	-4.386	-3.891	-6.058
A0A8I3PJ19	SWI/SNF related, matrix associated, actin dependent regulator of chromatin, subfamily a, member 4	SMARCA4	-4.700	-5.013	-4.087
A0A8I3MM38	Fibrillarin	FBL	-4.018	-7.114	-1.962
A0A8I3RZK8	Zinc finger CCHC-type containing 3	ZCCHC3	-3.665	-5.718	-3.381
A0A8P0STD7	40 S ribosomal protein S2	3 SV	-5.069	-5.791	-0.657
A0A8I3PKM8	Nuclear transcription factor, X-box binding 1	NFX1	-3.588	-4.875	-3.002
A0A8I3Q3M7	Cell division cycle 45	CDC45	-1.025	-7.065	-2.788
A0A8I3PMM3	Cellular tumor antigen p53	TP53	-2.145	-3.902	-4.705
A0A8I3N527	Nuclear RNA export factor 1	NXF1	-3.800	-5.267	-1.536
A0A8I3PYF0	FACT complex subunit	SUPT16H	-3.071	-5.592	-1.828
A0A8P0SQL8	Ribosomal protein L37a	3 SV	-3.965	-5.827	-0.663
A0A8I3MSY1	Nucleosome assembly protein 1 like 1	NAP1L1	-1.885	-4.066	-4.485
A0A8P0T777	WD repeat domain 70	WDR70	-2.919	-6.089	-1.424
A0A8I3NZ09	Transducin beta like 3	TBL3	-1.844	-3.417	-4.811
A0A8P0N906	DNA polymerase epsilon catalytic subunit	POLE	-2.923	-3.493	-3.655
A0A8I3P419	Serine/threonine-protein kinase PRP4 homolog	PRPF4B	-4.051	-3.781	-2.207
A0A8I3S988	Chromosome transmission fidelity factor 18	CHTF18	-1.533	-6.296	-2.166
A0A8I3MIZ2	Pumilio RNA binding family member 3	PUM3	-3.700	-3.651	-2.437
A0A8I3PVA9	CWC22 spliceosome associated protein homolog	CWC22	-3.135	-4.297	-2.187

### Gene ontology assignment to WCL proteins indicates different biological patterns in adenoma and carcinoma cell lines

To further identify biological processes implicated in the set of differentially abundant proteins from each subtype (adenoma, carcinoma) compared to the healthy control, Gene Ontology (GO) analysis was performed. Over-represented proteins from the adenoma ZMTH3 were primarily associated with *RNA splicing via transesterification reactions* (fold enrichment (F.E.) 3.0), *mRNA splicing via spliceosome* (F.E. 3.0), *mRNA processing* (F.E. 2.9), *intracellular transport* (F.E. 2.0), and *vesicle-mediated transport* (F.E. 2.0) (Fig. 2g). Likewise, GO analysis on the shared proteins from the three carcinoma cell lines showed that they were significantly involved in biological processes such as *intracellular protein transport* (F.E. 3.4), *vesicle-mediated transport* (F.E. 3.1), *cellular protein localization* (F.E. 3), *cell migration* (F.E. 2.7) and *cell motility* (F.E. 2.6) (Fig. 2h).

GO analysis on the differentially under-represented proteins from ZMTH3 showed significant enrichment in proteins associated with *ncRNA metabolic process* (F.E. 3.1), *nucleotide metabolic process* (F.E. 2.7), *carboxylic acid metabolic process* (F.E. 2.6), *oxoacid metabolic process* (F.E. 2.6), and again *intracellular transport* (F.E. 2.3) (Fig. 2i). In contrast, common under-represented proteins from the carcinoma cell lines identified pathways associated with *rRNA metabolic process* (F.E. 6.5); *ribosome biogenesis* (F.E. 6); *ncRNA processing* (F.E. 5.8); *mRNA processing* (F.E. 5) and *RNA processing* (F.E. 5) (Fig. 2j).

These results demonstrated that WCLs show a large variance in protein abundance patterns among CMT samples when compared to the healthy control. The observed changes were characterised in the adenoma subtype by alterations in abundance of proteins enriched for RNA processing pathways, which indicates a higher protein biosynthesis activity for subsequent cell growth; whereas carcinoma WCLs showed a high abundance of proteins enriched for migratory, motility and adhesion pathways, which may contribute to tumour development and progression. These biological patterns provided a distinctive phenotype for each CMT subtype, representing different pathways of tumorigenic nature.

### Extracellular vesicle proteomes show a higher variance than WCLs proteomes

To assess whether the identified differences among WCLs were also reflected in the proteomes of their excreted vesicles, exosomes were isolated from their respective growth media by size exclusion chromatography. As it was not possible with our method to accurately separate exosomes from other secreted extracellular vesicles (EVs) of similar size, these isolated vesicles are simply referred to as “EVs”.

**Fig. 3:**
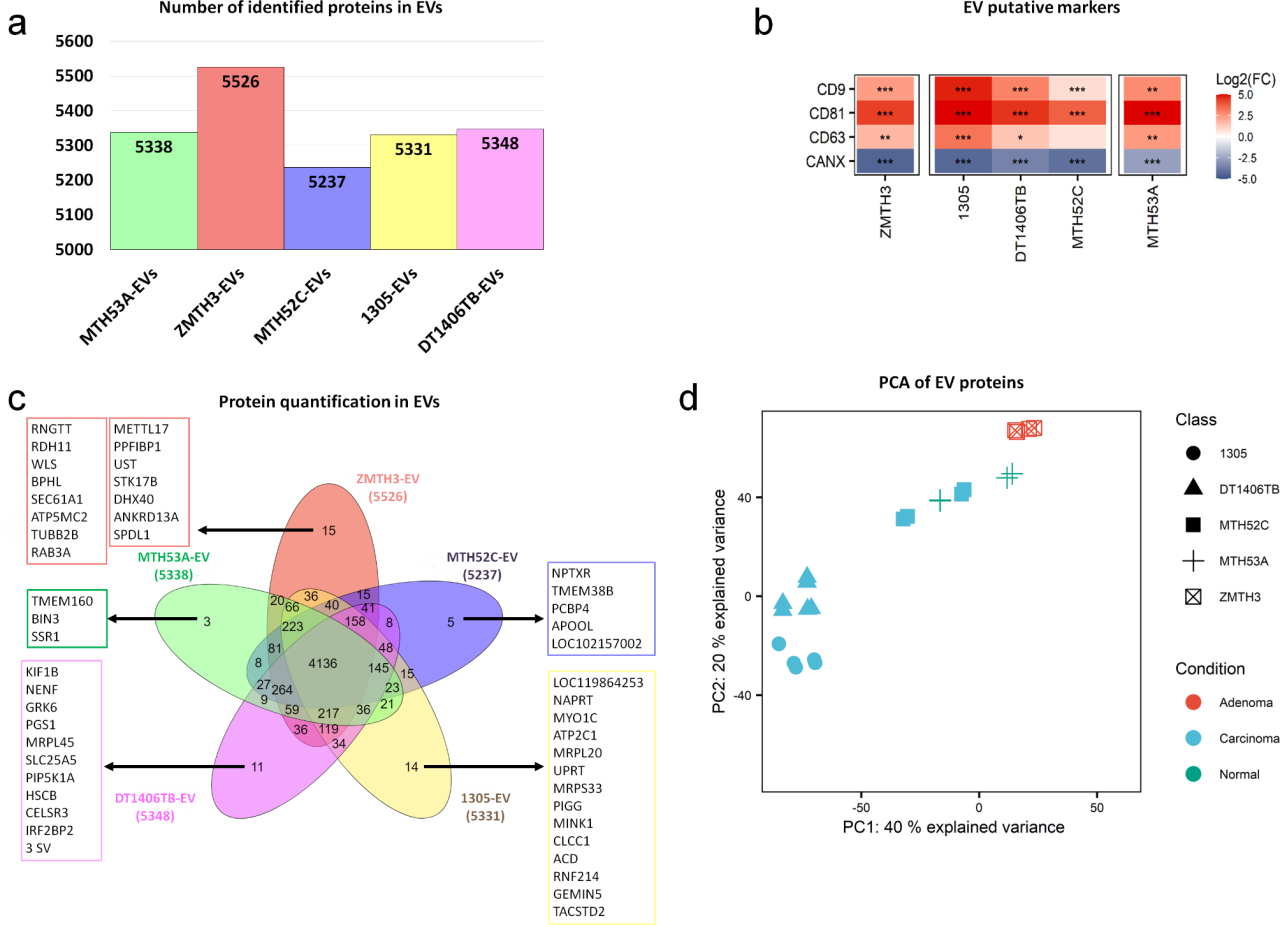
Mass spectrometry-based profiling of EV proteomes. **(a)** Protein groups identified in each EV-derived cell line. **(b)** Heatmap illustrating EV protein markers CD9, CD63, CD81 and calnexin (CANX) in EV-derived protein compared with the WCLs based on their log2-transformed fold-changes. **(c)** Overlap of protein intensities quantified in each EV-derived cell line. **(d)** Principal component analysis of EV proteins of healthy control and CMT cell lines

NTA characterisation of EV fractions showed particle concentrations ranging from 2.94 to 10.56 × 10^11^ particles/ml and the diameter ranged mostly from 120 to 230 nm (Additional file 4). For LC-MS/MS-based proteomics, biological duplicates for healthy control, adenoma, and simple carcinoma EVs (triplicates for both complex carcinoma EVs) and their technical replicates were analysed. Quantified proteins in EVs ranged from 5237 to 5526 (Fig. 3a). To confirm successful EV enrichment, the presence of putative EV markers CD9, CD63 and CD81 in our EV protein extracts was compared to their equivalent WCLs. All three markers were significantly more abundant in the EV protein extracts than in the WCLs (Fig. 3b). In contrast, calnexin (CANX), a protein used as an indicator of intracellular contamination [50], was under-represented in all EV-derived protein extracts. CD9 and CD63 EV markers were also confirmed by western blot (Additional file 5). These results are consistent with a significant enrichment of EVs and very low contamination with non-EV proteins.

Evaluation of the number of unique and jointly identified EV proteins in each cell line (Fig. 3b) found that similarly to WCLs (Fig. 1b) the majority of proteins (4136) was still identified in all EV samples; however, the percentage of proteins found in all EV fractions was noticeably lower than in the WCLs (69.7% in EVs vs. 84.4% in WCLs). Similarly, the number of unique proteins in EVs, though still very small with only 3–15 proteins, was higher in the complex carcinoma DT1406TB (11 vs. 3), complex carcinoma 1305 (14 vs. 9), and adenoma ZMTH3 (15 vs. 5) EV protein fractions. Hence, the EV proteomes differed more strongly between CMT cell lines than the WCL proteomes.

Despite the very small number of unique proteins in each EV isolate, PCA analysis (Fig. 3d) confirmed a higher variation between the EV proteomes of each cell line compared to the WCLs. Surprisingly, the healthy control MTH53A did not only cluster together with the adenoma cell line ZMTH3 but was also closer to the morphologically similar simple carcinoma cell line MTH52C (Additional file 1). In contrast, EV proteomes of the two complex carcinoma cell lines DT1406TB and 1305 together formed a separate cluster. These results emphasise again the similarities between protein abundance patterns of the healthy control MTH53A and adenoma ZMTH3 cells. It also showed that the EV proteomes did not allow significant separation of normal/benign cell lines from the simple carcinoma MTH52C as was the case for the WCLs.

### EVs display larger differences in protein abundance compared to their corresponding WCLs

**Fig. 4:**
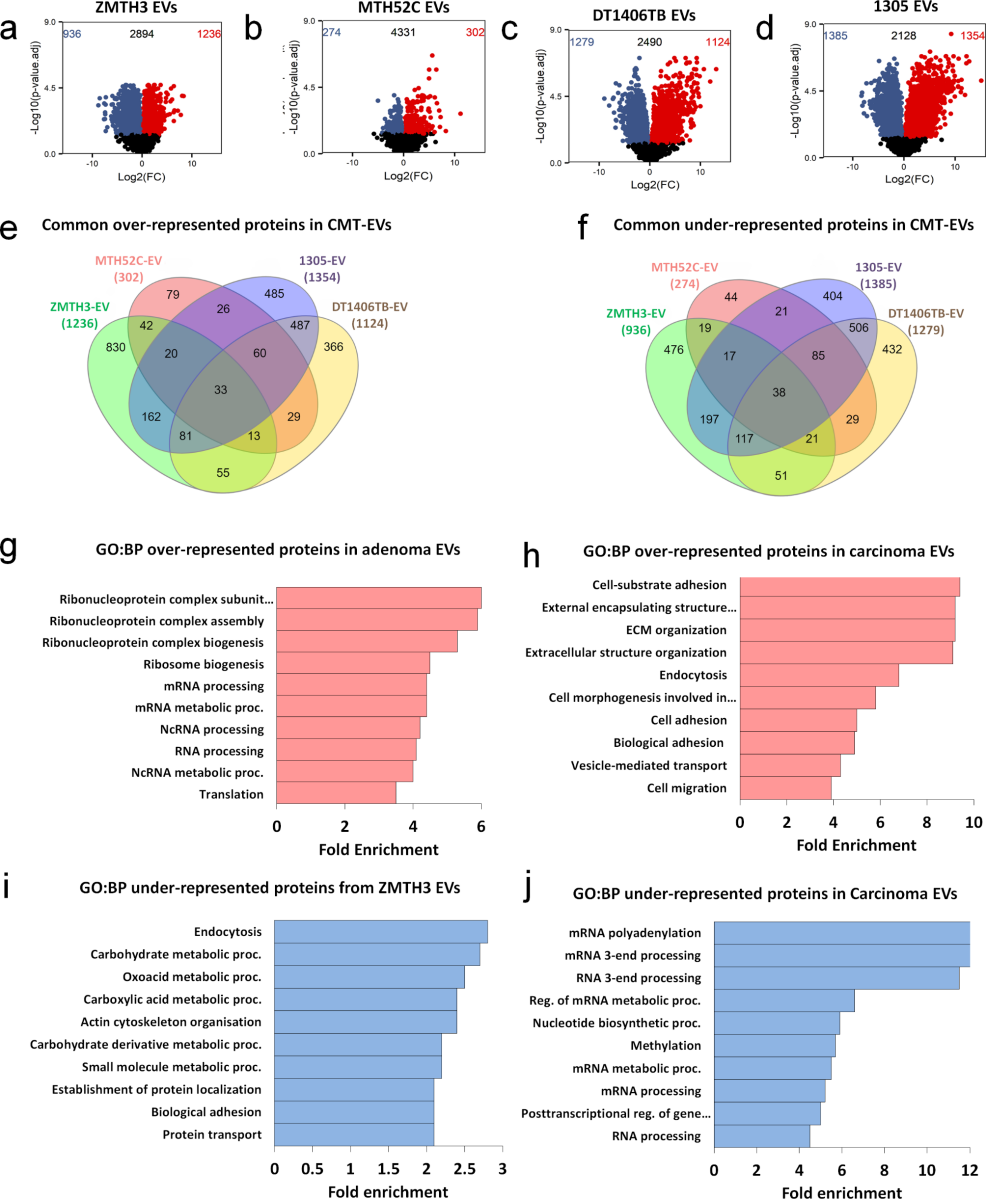
Differential analysis in EV protein abundance levels of CMTs when compared to healthy control MTH53A. Volcano plots of **(a)** adenoma ZMTH3, **(b)** simple carcinoma MTH52C, **(c)** complex carcinoma DT1406TB, and **(d)** complex carcinoma 1305, when compared to the healthy control. Red dots in the top right area were over-represented in the CMT cell line relative to MTH53A. Blue dots in the top left area were under-represented in CMT cell line relative to MTH53A. Black dots below the dashed line represent proteins with no statistical difference (*p* > 0.05). **(e)** Venn diagram showing the overlap between the EV proteins over-represented (*p* < 0.05) in all the CMT cell lines. **(f)** Gene ontology of over-represented ZMTH3 EV proteins enriched in biological process pathways (GO: BP) expressed as fold enrichment scores (F.E.), considering a false discovery rate (FDR) < 0.05. **(g)** Gene ontology of common over-represented EV proteins of carcinoma cell lines enriched in biological process pathways (GO: BP). **(h)** Venn diagram showing the overlap between the EV proteins under-represented (*p* < 0.05) in all the CMT cell lines. **(i)** Gene ontology of under-represented ZMTH3 EV proteins enriched in biological process pathways (GO: BP). **(j)** Gene ontology of common under-represented EV proteins of carcinoma cell lines enriched in biological process pathways (GO: BP)

The EV proteome of the adenoma (ZMTH3) showed significant differences from that of the MTH53A cell line (non-neoplastic) when protein abundance levels were directly compared, with 2172 differentially abundant proteins (Fig. 4a). Surprisingly, EV proteomes from the three carcinoma cell lines contained fewer significantly abundant proteins than the WCLs when compared to the healthy control. The simple carcinoma MTH52C EV proteome only had 576 differentially abundant proteins (Fig. 4b), while complex carcinomas 1305 and DT1406TB EVs had 739 and 2403 differentially abundant proteins, respectively (Fig. 4c-d).

Top over-represented EV proteins from the adenoma ZMTH3 (Table 4) were mainly related to immune response (ISG15, BST2, IFI44), while top under-represented proteins identified in the adenoma ZMTH3 EVs were associated with migratory and adhesion activity (MXRA8, TNN, SERPINB8) (Table 4), similar to the WCLs.

**Table 4:** Top 10 differentially over- and under-represented proteins identified in adenoma EVs vs. healthy control

Over-represented proteins			
**Accession number**	**Protein**	**Log2FC**	**Adj. P-value**
A0A8I3QAX6	CTPS2	8.361	1.17E-04
A0A8I3NTB5	CANT1	8.065	1.12E-04
A0A8I3PAV5	TCN2	7.800	2.25E-03
A0A8I3P342	ISG15	7.219	1.16E-03
A0A8I3MLA0	MFAP2	6.528	1.10E-03
A0A8P0TVC6	ADAP1	6.482	1.24E-03
A0A8I3PFH2	ZPLD1	6.409	2.46E-05
J9NVI2	BST2	6.396	5.88E-03
A0A8I3MKR1	IFI44	6.365	1.49E-03
A0A8P0N757	PRSS23	6.058	2.00E-04
**Under-represented proteins**			
**Accession number**	**Protein**	**Log2FC**	**Adj. P-value**
A0A8I3PNQ7	MXRA8	-8.773	4.98E-04
A0A8I3N7R9	ANGPTL2	-8.497	1.65E-04
A0A8I3Q6P3	TPM4	-7.629	4.39E-03
A0A8I3NDQ9	TNN	-7.596	3.69E-04
A0A8I3Q497	TPM4	-7.441	4.26E-03
A0A8I3PGJ4	PTX3	-7.425	1.51E-03
A0A8I3MER3	NN*	-7.400	2.04E-03
A0A8I3NUH5	NDUFB8	-7.308	4.40E-04
A0A8P0T552	RBP4	-7.285	2.86E-02
A0A8P0S626	SERPINB8	-7.266	1.40E-04

*Rad60-SLD domain-containing protein

Furthermore, top over-represented proteins in carcinoma EVs (Additional file 6) included ECM-related proteins associated with migration and adhesion (LUM, COL14A1, FN1) whereas top under-represented proteins from carcinoma EVs included proteins associated with RNA processing (ELAVL2, SNU13, SARS2) (for associations and references of proteins listed in Table 4 and Additional file 5 with cancer studies, refer to Additional file 7).

### CMT-derived EVs contained a set of common proteins with distinct biological behaviour

The number of proteins that were over-represented in all CMT-derived EVs (Fig. 4e) was significantly lower than in the WCLs (33 vs. 226 common proteins; Fig. 2e). Among the most abundant proteins, based on the average of all four cell lines, were ECM-associated glycoproteins OLFML2B and THBS2, as well as the proteoglycan LUM and matrix-metalloproteinase MMP19 (Table 5).

**Table 5:** Top 20 differentially common under-represented EV proteins vs. healthy control in CMT cell lines

Common over-represented proteins						
**Accession number**	**Protein name**	**Protein abbreviation**	**ZMTH3 Log2FC**	**MTH52C** **Log2FC**	**1305** **Log2FC**	**DT1406TB** **Log2FC**
A0A8I3NTB5	Galectin-3-binding protein	CANT1	8.065	3.700	12.760	9.220
A0A8P0N757	Serine protease 23	PRSS23	6.058	3.657	8.085	8.682
A0A8I3Q308	Olfactomedin like 2B	OLFML2B	1.900	5.182	7.641	9.408
A0A8I3P684	Lumican	LUM	2.505	11.155	1.433	8.507
A0A8I3PKT4	Complement C1s	C1S	4.888	4.440	9.560	3.265
A0A8I3N2B1	Beta-1,3-N-acetylglucosaminyltransferase	LFNG	2.676	4.905	7.775	6.669
A0A8I3QK41	Pirin	PIR	1.312	3.451	4.550	8.350
A0A8I3S0C0	Lysyl oxidase homolog	LOXL3	0.979	2.957	7.004	6.529
A0A8I3PRC1	Semaphorin-3 C	SEMA3C	3.698	1.429	5.501	4.237
A0A8I3MTX4	Matrix metallopeptidase 19	MMP19	3.141	3.013	3.129	4.508
A0A8I3MIR7	Lysophosphatidylcholine acyltransferase 2	LPCAT2	0.925	2.086	3.281	7.370
A0A8I3PN55	BRO1 domain-containing protein	PDCD6IP	1.429	3.837	5.822	2.288
A0A8I3S0A8	Tripartite motif containing 25	TRIM25	3.737	2.188	4.888	1.959
A0A8P0NAE9	Calpastatin	CAST	1.081	3.637	5.163	2.655
A0A8I3NB75	Thrombospondin 2	THBS2	1.132	0.915	2.530	7.351
A0A8I3PVI8	LRR binding FLII interacting protein 2	LRRFIP2	1.281	1.685	2.837	6.001
A0A8P0TPH1	Ras-related protein Rab-4	RAB4A	2.203	2.294	3.509	2.816
Q38JA9	Caspase 8	CASP8	1.839	1.735	2.756	3.669
A0A8I3Q6P2	Progesterone receptor membrane component 2	PGRMC2	1.278	0.761	4.155	3.248
P06625	Signal recognition particle receptor subunit alpha	SRPRA	2.452	1.162	3.140	2.625
**Common under-represented proteins**						
**Accession number**	**Protein name**	**Protein abbreviation**	**ZMTH3** **Log2FC**	**MTH52C** **Log2FC**	**1305** **Log2FC**	**DT1406TB** **Log2FC**
A0A8P0SLL9	Exportin 6	XPO6	-1.488	-5.010	-7.770	-6.686
A0A8I3NJG0	TatD DNase domain containing 1	TATDN1	-3.524	-1.490	-5.855	-3.802
A0A8I3MMH6	Chromosome 4 C5orf22 homolog	C4H5orf22	-2.712	-2.001	-3.975	-4.296
A0A8I3PRT9	Inosine-5’-monophosphate dehydrogenase	IMPDH1	-3.416	-2.789	-4.010	-1.788
A0A8I3Q4 × 2	Ubiquitin specific peptidase 19	USP19	-0.530	-1.699	-3.658	-5.477
A0A8I3QXV7	GDP-mannose pyrophosphorylase A	GMPPA	-0.529	-2.945	-3.164	-4.572
A0A8I3MYE7	Regulation of nuclear pre-mRNA domain-containing protein	RPRD1A	-3.296	-1.052	-4.263	-2.261
A0A8I3S4H8	Adenosylhomocysteinase like 2	AHCYL2	-2.212	-2.263	-4.242	-1.195
A0A8I3PIR4	Fibroblast growth factor 7	GALK2	-1.182	-1.436	-1.055	-5.984
A0A8I3NSZ0	Argininosuccinate lyase	GUSB	-1.617	-1.238	-3.938	-2.246
A0A8P0NK55	Dipeptidyl peptidase 9	DPP9	-2.086	-1.861	-3.587	-1.368
A0A8I3MP00	TIP41-like protein	TBX19	-0.767	-0.800	-4.802	-2.381
A0A8P0TU84	Core-binding factor subunit beta	CBFB	-1.526	-0.825	-3.184	-3.002
A0A8I3PTA7	Dihydrolipoyl dehydrogenase, mitochondrial	DLD	-2.055	-2.534	-2.086	-1.690
A0A8I3Q7R1	Inosine-5’-monophosphate dehydrogenase	IMPDH2	-1.877	-1.302	-3.304	-1.716
A0A8I3NZF6	ATPase GET3	GET3	-0.356	-0.947	-3.553	-2.850
A0A8I3PVF4	Methylthioribose-1-phosphate isomerase	MRI1	-0.989	-2.024	-1.683	-2.792
A0A8I3N997	Polyadenylate-binding protein	PABPC4	-2.455	-1.357	-1.701	-1.906
A0A8I3PG05	Mevalonate kinase	MVK	-0.321	-1.320	-3.876	-1.746
A0A8P0TF63	Pseudouridine synthase 7	PUS7	-1.685	-0.915	-2.967	-1.626

Carcinoma EVs shared 60 over-represented proteins, including ECM proteins involved in cell adhesion, motility, wound healing and maintenance of cell shape (FN1, BGN, HAPLN1) (Table 6), which have all been previously identified in human breast cancer and canine mammary cancer studies [51–53]. The EVs of the two complex carcinoma cell lines shared 487 over-represented proteins, mostly associated with angiogenesis, cell migration, adhesion and motility (ECM1, EDIL3, VEGFC) (Additional file 8).

**Table 6:** Top 20 differentially common over-represented EV proteins vs. healthy control in the carcinoma cell lines

Common over-represented proteins					
**Accession number**	**Protein name**	**Protein abbreviation**	**MTH52C** **Log2FC**	**1305** **Log2FC**	**DT1406TB** **Log2FC**
A0A8I3NGU2	Folate_rec domain-containing protein	LOC476816	7.242	8.684	9.315
A0A8P0TLW8	Scavenger receptor cysteine rich family member with 5 domains	SSC5D	8.289	10.690	5.018
Q28275	Fibronectin (Fragment)	FN1	4.837	6.517	10.712
A0A8P0SNB9	Biglycan	BGN	4.906	7.438	8.390
A0A8I3PAS0	Hyaluronan and proteoglycan link protein 1	HAPLN1	6.534	2.065	11.965
A0A8I3MGS0	72 kDa type IV collagenase	MMP2	3.103	6.260	8.838
A0A8I3Q318	Complement factor I	CFI	3.497	4.469	8.752
A0A8I3MZE8	GM2 ganglioside activator	GM2A	4.393	5.453	6.426
A0A8I3PHI4	Fibronectin	FN1	3.907	4.087	7.910
A0A8I3PMB5	Secretogranin-3	SCG3	4.982	2.825	7.256
A0A8I3PPU8	Thrombospondin 1	THBS1	1.870	7.201	5.508
A0A8P0P6 × 6	Cadherin-2	CDH2	2.801	4.917	5.930
A0A8I3Q5D9	Procollagen C-endopeptidase enhancer 2	PCOLCE2	2.813	3.477	7.098
A0A8I3MTJ9	Laminin subunit gamma 1	LAMC1	3.385	5.938	3.625
A0A8I3PM12	L1 cell adhesion molecule	L1CAM	1.683	7.241	3.972
A0A8I3PNM0	G_PROTEIN_RECEP_F3_4 domain-containing protein	GPRC5D	1.755	6.742	3.767
A0A8P0TM99	Pleckstrin homology like domain family B member 2	PHLDB2	2.235	6.263	3.720
A0A8P0SGF7	Hyaluronan and proteoglycan link protein 3	HAPLN3	3.031	3.779	5.017
A0A8I3NXQ5	Ethylmalonyl-CoA decarboxylase	3 SV	1.567	4.820	5.429
A0A8I3S4E2	Versican	VCAN	1.607	4.248	5.805
**Common under-represented proteins**					
**Accession number**	**Protein name**	**Protein abbreviation**	**MTH52C** **Log2FC**	**1305** **Log2FC**	**DT1406TB** **Log2FC**
A0A8I3Q193	Selenocysteine lyase	SCLY	-1.972	-5.637	-5.324
A0A8I3NJ98	N-sulfoglucosamine sulfohydrolase	SGSH	-2.091	-4.984	-4.477
A0A8I3QBZ4	Taxilin gamma	TXLNG	-0.878	-4.915	-5.585
A0A8P0SNX6	tRNA: m(4)X modification enzyme TRM13	TRMT13	-2.591	-5.158	-3.569
A0A8P0TLB2	UBX domain-containing protein 7	UBXN7	-2.140	-4.895	-3.880
A0A8I3MKV2	Malignant T-cell-amplified sequence	LOC487150	-2.309	-4.124	-3.935
A0A8I3PUG8	TATA-box binding protein	TBP	-2.315	-3.893	-4.015
A0A8I3RWI4	Section 1 family domain containing 2	SCFD2	-2.539	-2.898	-4.163
A0A8I3PMJ4	DIS3-like exonuclease 2	DIS3L2	-3.638	-2.854	-2.612
A0A8I3N7E1	CTP synthase	CTPS1	-2.172	-3.350	-3.558
A0A8I3MKB3	CYRIA-B_Rac1-bd domain-containing protein	CYFIP2	-2.791	-3.719	-2.455
A0A8I3RYJ8	Lysine demethylase 2 A	KDM2A	-0.797	-4.070	-4.012
A0A8I3RXS7	2-(3-amino-3-carboxypropyl)histidine synthase subunit 1	DPH1	-2.365	-2.992	-3.444
A0A8I3RW28	ASPSCR1 tether for SLC2A4, UBX domain containing	ASPSCR1	-1.848	-4.301	-2.302
A0A8I3RX66	thioredoxin-disulfide reductase	TXNRD3	-1.867	-4.939	-1.281
A0A8I3NN15	Small nuclear ribonucleoprotein Sm D1	SNRPD1	-0.857	-4.393	-2.586
A0A8P0NJW0	Metastasis associated 1	MTA1	-1.104	-4.682	-1.985
A0A8I3S647	Propionyl-CoA carboxylase alpha chain, mitochondrial	PCCA	-2.235	-3.662	-1.763
P10463	Calcyphosin	CAPS	-1.802	-2.734	-2.894
A0A8P0SK07	Complement component 1 Q subcomponent-binding protein, mitochondrial	C1QBP	-0.944	-3.812	-2.622

Evaluation of the overlaps of the under-represented proteins in all CMT EVs identified only 38 common proteins mostly related to DNA/RNA processing (TATDN1, IMPDH1, IMPDH2, PAPBC4, PUS7) (Fig. 4f; Table 5). Interestingly the least abundant protein amongst them, XPO6, has previously been reported to be downregulated in human breast cancer (Additional file 7). Moreover, carcinoma EVs shared 85 under-represented proteins involved in regulating the cellular protein metabolism (ASPSCR1, DIS3L2, C1QBP) (Table 6), whereas complex carcinoma EVs shared 506 under-represented proteins, with the least abundant being involved in RNA processing and cell cycle (WDR3, TP53, BRIX1, RPS13) (Additional file 8).

### Gene ontology of EV-derived proteins revealed similar biological patterns as WCLs

All over-represented proteins in the adenoma ZMTH3 EVs were again mainly associated with GO biological processes such as *ribonucleoprotein complex subunit organisation* (F.E. 6.0); *ribosome biogenesis* (F.E. 4.5); *mRNA processing* (F.E. 4.4); *RNA processing* (F.E. 4.0) and *translation* (F.E. 3.5) (Fig. 4g). Likewise, GO analysis of the common over-represented proteins identified in the carcinoma EVs were enriched for proteins associated with *cell-substrate adhesion* (F.E. 9.4); *ECM organisation* (F.E. 9.2); *endocytosis* (F.E. 6.8); *cell adhesion* (F.E. 5); *vesicle-mediated transport* (F.E. 4.3) and *cell migration* (F.E. 3.9) (Fig. 4h).

Under-represented proteins from the adenoma ZMTH3 EVs were associated with endocytosis (F.E. 2.8); carbohydrate metabolic process (F.E. 2.7); oxoacid metabolic process (F.E. 2.4); actin cytoskeleton organisation (F.E. 2.4); establishment of protein localization (F.E. 2.1) and biological adhesion (F.E. 2.1) pathways (Fig. 4i), whereas shared under-represented proteins in carcinoma EVs were associated with RNA polyadenylation (F.E. 16), RNA 3- end processing (F.E. 11.5), reg. of mRNA metabolic process (F.E. 6.6), RNA catabolic process (F.E. 6), methylation (F.E. 5.7) and mRNA processing (F.E. 5.2) (Fig. 4j).

Overall, CMT cell line-derived EV proteomes presented patterns of protein abundance different to their equivalent WCLs, indicating potential selectivity. Nevertheless, these proteomes still reflected the same biological behaviour of their parent cells (WCLs). While the adenoma subtype was highly abundant in proteins enriched for RNA splicing process, the carcinoma subtype had a high abundance of proteins associated with ECM organisation, a main component of the tumour microenvironment, potentially facilitating cancer progression by promoting cell migration.

### EV proteomes allow identification of potential biomarkers for disease state

Given the potential of EV protein cargo to distinguish between different types of CMT, we sought to identify signatures that could serve as biomarkers for each of the tested CMT types based on the abundance of WCL and EV proteins in the CMT cell lines.

**Fig. 5:**
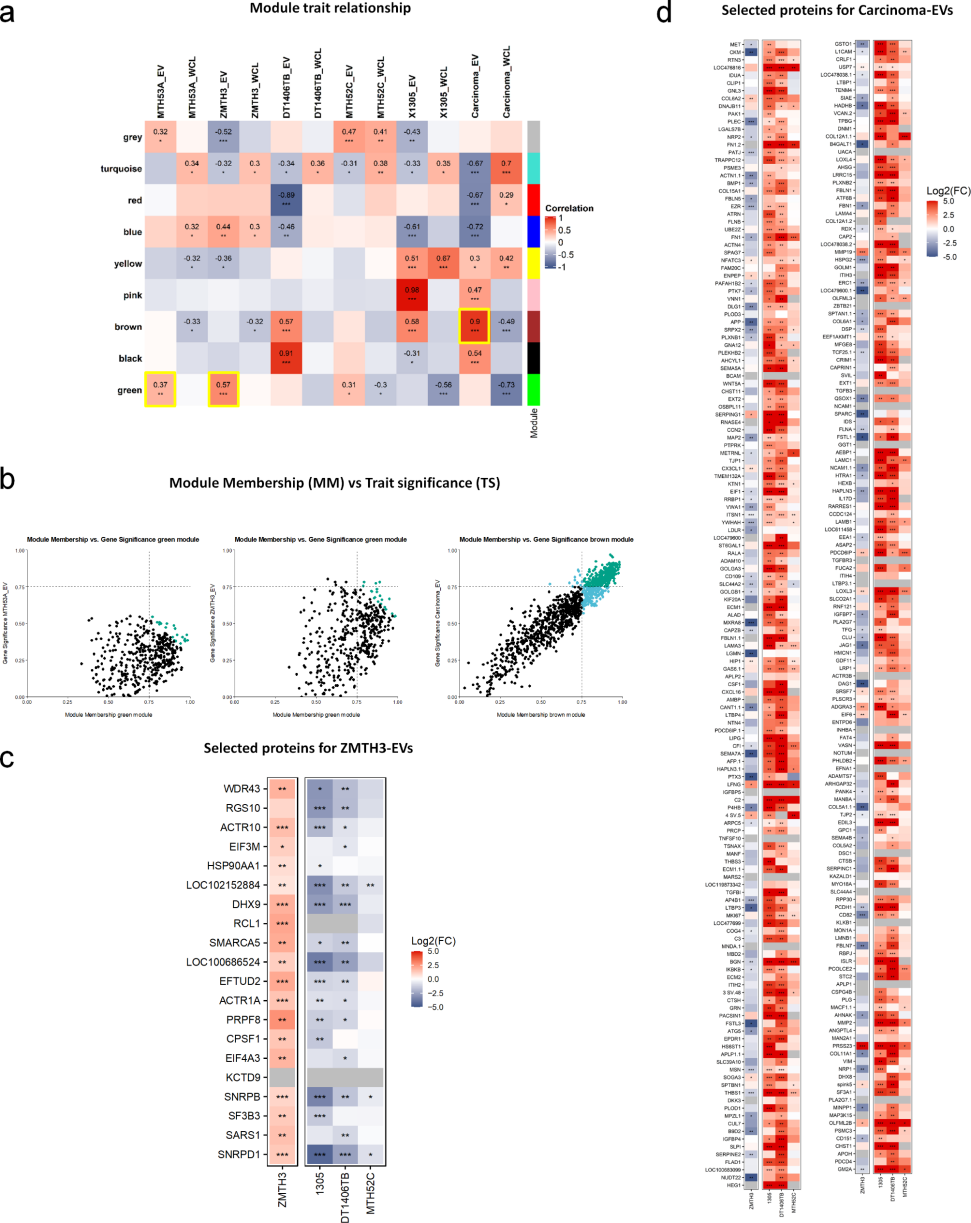
Selection of protein “key drivers” for further EV proteomic signatures. **a)** Heatmap of the EV proteomic profiles selected for each cell line (protein “key drivers”). **(a)** Module trait relationship. Pearson correlation of co-abundant proteins for each trait (Sample types: WCL and EVs; groups: carcinoma, simple carcinoma, and complex carcinoma) in every module colour. The significance of the correlation is indicated with asterisks (* *p* ≤ 0.05, ** *p* ≤ 0.01, *** *p* ≤ 0.001). Yellow squares highlight the modules of interest for further key driver identification in each subtype (EVs). **(b)** Module membership (MM) vs trait significance (TS) with a correlation cut-off ≥ 0.75. From left to right: selected module for healthy control MTH53A; selected module (green) for adenoma ZMTH3; selected module (brown) for Carcinoma. Real “key drivers” are shown in green colour. **(c)** Adenoma ZMTH3 top 20 selected protein key driver. **(d)** All three carcinoma cell lines-derived protein key drivers. Significance of log2FCs is indicated with asterisks (* *p* ≤ 0.05, ** *p* ≤ 0.01, *** *p* ≤ 0.001)

To identify co-abundant proteins that may play an important role in the progression of canine mammary cancer, a weighted gene correlation network analysis (WGCNA) was performed on all available data obtained from the EVs and WCLs of each cell line. Hierarchical cluster analysis identified 9 modules of co-abundant proteins, identified by colours (Additional file 9). Module-trait relationships based on Pearson correlation were assessed to identify modules highly correlating with particular traits (e.g. EV and WCL samples as well as carcinoma and complex carcinoma groups) (Fig. 5a). The most representative modules for each cell line were selected according to their positive correlation (green module for healthy control MTH53A and adenoma ZMTH3 EVs: correlation 0.37 and 0.57, respectively; brown module for carcinoma EVs: correlation 0.9) (Fig. 5a). For these module-trait combinations, absolute module memberships (MM) and trait significances (TS) were determined, and proteins were considered potential key drivers if both values were ≥ 0.75 (Fig. 5b). Using this approach, all proteins in EVs derived from the healthy control MTH53A were below the correlation cut-off (≥ 0.75). Therefore, key drivers could not be identified. Only four key drivers were identified for the adenoma ZMTH3 EVs. Hence, absolute MM and TS values were summed to identify the top 20 key driver candidates for ZMTH3 (Fig. 5c), which were again mostly associated with RNA processing, mRNA processing, and RNA splicing (Table 7). Finally, proteins highly correlating with both simple and complex carcinoma (Fig. 5d) were mainly related to pathways including ECM organisation, cell adhesion, cell motility, and cell migration (Table 7), including ECM-associated proteins previously associated with mammary cancer, such as BGN and FN1 (Additional file 7). As BGN was one of the top most over-represented proteins in the carcinoma signature, western blot was performed on new, independent EV isolates (Additional file 10), which confirmed higher abundance of BGN in the carcinoma EVs. We further performed immunofluorescence to assess the protein abundance in the parent cell lines. Consistent with our proteomic analysis data, no significant differences were found between the cancer cell lines and the normal or adenoma cell lines (Additional file 11). Therefore, BGN may prove useful as a specific EV biomarker for canine mammary carcinomas.

**Table 7:** GO pathway categories from protein profiles (key drivers) in each cell line derived EVs

Cell type/Cell line	GO pathway categories	Proteins
Adenoma ZMTH3	RNA processing	CPSF1 WDR43 EIF4A3 SNRPB DHX9 EFTUD2 SNRPD1 PRPF8 SF3B3
	mRNA processing	CPSF1 EIF4A3 SNRPB DHX9 EFTUD2 SNRPD1 PRPF8 SF3B3
	mRNA metabolic process	CPSF1 EIF4A3 SNRPB DHX9 EFTUD2 SNRPD1 PRPF8 SF3B3
	RNA splicing	EIF4A3 SNRPB DHX9 EFTUD2 SNRPD1 PRPF8 SF3B3
Simple + complex carcinoma	ECM organisation	MMP19 FBLN1 EXT1 TGFBI B4GALT1 ECM2 COL15A1 NTN4 LOXL3 APP PTX3 LOXL4 MMP2 PHLDB2 FBLN5 DAG1 COL6A1 QSOX1 OLFML2B LAMC1 LTBP3 PLOD3 ADAMTS7 COL5A2 VWA1 COL11A1 KAZALD1
	Cell adhesion	EZR PLXNB2 FBLN1 EXT1 TGFBI PTPRK PTK7 B4GALT1 ECM2 IGFBP7 NRP1 FAT4 LAMA4 BCAM TENM4 JAG1 ACTN4 P4HB PCDH1 ATRN NTN4 SPINK5 LOXL3 EDIL3 THBS1 CX3CL1 DSP PHLDB2 HAPLN3 PLXNB1 LAMC1 DLG1 NCAM1 RDX FN1 FBN1 ADAM10 MSN THBS3 EFNA1 SRPX2 DSC1 LAMA3 MXRA8 L1CAM FLNA CSF1 FSTL3 LAMB1 WNT5A CCN2 EPDR1
	Cell motility	PLXNB2 PLG FBLN1 EXT1 PTPRK PTK7 B4GALT1 PLA2G7 MET NRP1 LAMA4 PRCP PAK1 JAG1 ATRN NTN4 APP THBS1 CX3CL1 SEMA5A PHLDB2 LGMN FSTL1 DAG1 APOH ENPEP ECM1 GPC1 PLXNB1 NRP2 LAMC1 ARPC5 LRRC15 CTSH GRN RDX FN1 IGFBP5 CXCL16 ADAM10 MSN EFNA1 SRPX2 SPARC SEMA7A LAMA3 MYO18A L1CAM FLNA CSF1 TGFBR3 CD151 LAMB1 WNT5A GNA12
	Cell migration	PLXNB2 PLG EXT1 PTPRK PTK7 B4GALT1 PLA2G7 MET NRP1 LAMA4 PRCP PAK1 JAG1 ATRN NTN4 APP THBS1 CX3CL1 SEMA5A PHLDB2 LGMN FSTL1 DAG1 APOH ENPEP ECM1 GPC1 PLXNB1 NRP2 LAMC1 ARPC5 LRRC15 CTSH GRN RDX FN1 IGFBP5 CXCL16 ADAM10 MSN EFNA1 SRPX2 SPARC SEMA7A LAMA3 MYO18A L1CAM FLNA CSF1 TGFBR3 CD151 LAMB1 WNT5A GNA12

## Discussion

Over the last decades, EVs have come to the forefront of cancer research due to their role in transferring disease-related signalling molecules, thereby facilitating cell-to-cell communication [1, 54]. Indeed, EV proteins released by highly invasive breast cancer cells can affect the growth and metastatic progression of more benign human breast cancer cells [12, 55]. Previous studies in dogs aimed at characterising EVs in CMT cell lines mainly focused on miRNA content [56]. Here we present the first proteomic analysis of EVs from normal, benign and malignant canine mammary cell lines to identify distinct protein profiles. Such signatures could be used to differentiate between normal mammary gland and CMTs, between CMT types (carcinoma and adenoma), as well as between carcinoma subtypes (simple and complex). Our results form the basis for the development of a potential new serum-EV-based diagnostic procedure for CMT.

PCA analysis and hierarchical clustering (Figs. 1c and 3d **+ 5**) confirmed that proteins identified in WCLs and EVs depend on CMT subtype. One exception was the simple carcinoma MTH52C EV proteome, which clustered together with the healthy and benign control despite a higher variance in WCL sample types. This was also reflected by a reduced number of differentially abundant proteins in these EVs compared to the two other carcinoma-derived cell lines and may reflect the similar 2D morphology of simple carcinoma MTH52C and healthy control MTH53A cell lines. Nevertheless, simple carcinoma MTH52C cell line EVs contained a number of proteins that were also found in the two other carcinoma cell lines and are therefore likely to reflect their nature as carcinoma-derived cells. Tumours are diverse and therefore phenotypic diversity of canine mammary tumours can be expected to be accompanied by a respective variance in protein abundance patterns, as in human breast cancer [57].

Unlike the carcinoma cell lines, the adenoma ZMTH3 was highly abundant in proteins involved in RNA processing and RNA splicing in WCLs and EVs, respectively, processes that are major mediators of proteome diversity by interacting in more than one cancer hallmark, establishing complex regulatory networks that simultaneously coordinate multiple cancer characteristics [58]. One possible explanation for this biological difference between the adenoma and carcinoma cell types is that RNA-splicing factors may be required for cancer initiation, while being dispensable for tumour maintenance [59]. Interestingly, a recent proteomic study identified an enrichment of mRNA processing-associated proteins among phosphoproteins down-regulated in metastatic human breast cancer patients compared to non-metastatic cancers [60], indicating that a change in mRNA processing may be a specific trait for more aggressive mammary cancers.

The carcinoma subtype showed high abundance of proteins enriched for cell migration, adhesion, and motility in both WCLs and EV proteomes consistent with an increased migratory ability of carcinoma cells [61]. This was highlighted by a high over-representation of proteins associated with cytoskeleton organisation in the carcinoma cell-line WCLs. Moreover, reorganization of intermediate filaments, such as vimentin (average log2-fold change 1.23 in WCLs), in tumour cells is associated with epithelial-mesenchymal transition (EMT), promoting migratory and invasive activity of cancer cells of more aggressive phenotypes [61, 62]. Further, the carcinoma-derived EVs had a high abundance of ECM-related proteins consistent with their role in facilitating cell survival, growth, migration and invasion of cells [63].

It was noticeable that EVs did not purely reflect the parental WCL protein content but showed different protein abundance patterns; yet the same biological behaviour was still described in each CMT subtype. Nevertheless, it remains unclear whether there is a selective mechanism for sorting proteins into EVs in these cells. The higher concentration of specific proteins in EVs compared to WCLs suggests that there may be a targeted mechanism to direct these proteins into EVs, which could be cancer-specific [64]. Further studies should determine whether such an EV protein cargo sorting mechanism responds to a functional purpose, and what the biological effect of this selection in the tumour microenvironment is.

Co-abundant proteins were identified for the adenoma and carcinoma EV subtypes by WGCNA (Fig. 5). With this approach, proteins showing specific regulation in carcinoma EVs were identified, which may serve as potential biomarkers and/or therapeutic targets for CMT in the future. Unfortunately, the signature for the adenoma ZMTH3 was composed of proteins that did not show convincing correlation with the module and the trait, indicating that the selection of protein key drivers is not representative enough to establish a reliable adenoma signature based on these data. Nevertheless, the identified potential candidates show consistent and specific regulation over the different conditions investigated here, suggesting them to be a suitable starting point for the development of a representative and reliable signature. Notably, 9 out of the 20 co-abundant proteins were associated with RNA processing, mRNA processing and RNA splicing, correlating with the gene ontology results in the EV proteome and thus providing functional insights into the adenoma CMT phenotype. Further investigations are needed to establish a specific signature for benign mammary tumours in dogs.

Despite the differences in EV protein abundance between simple carcinoma MTH52C and complex carcinoma cell lines, there were expected similarities in biological processes resulting in a clear carcinoma protein pattern. Thus, the carcinoma signature included many of the most over-represented proteins that were strongly associated with ECM organisation, cell adhesion, cell motility, and cell migration, similar to results from previous proteomic studies of human breast cancer EVs [12, 54]. This is also consistent with observations from a previous cDNA microarray study on spontaneous CMTs, in which the majority of differentially expressed genes coded for proteins involved in cell motility, cytoskeletal organisation and ECM production [65]. In addition, proteomic analysis of cancer-associated stroma from 14 formalin-fixed paraffin-embedded canine mammary carcinomas also identified an over-representation of proteins associated with the ECM and cytoskeleton [66]. These observations show that the proteomes of the isolated EVs not only resemble those of the total proteomes of the CMT subtype but also reflect the biological processes related to carcinogenesis, both in vitro and in vivo. Taken together, our data is consistent with previous evidence that EVs could play a crucial role in cell behaviour changes, which may lead to cancer progression and therefore have strong potential for diagnostic purposes.

Two proteins included in the carcinoma EV signature were the ECM proteins biglycan (BGN) and fibronectin (FN1), which were among the most over-represented ECM-related proteins in EV isolates from all carcinoma cell lines (Table 6; Fig. 5c). BGN plays major roles in cellular processes including migration, adhesion, inflammation, cell growth and apoptosis [67]. In dogs, BGN upregulation has been identified in cancer-associated stroma of malignant CMTs, both in proteomic and transcriptomic approaches, and these results were consistent with human breast cancer studies [52]. FN1 is a major component of the ECM involved in cell adhesion, proliferation, migration, blood coagulation, wound healing and embryogenesis [51]. In human breast cancer tissues, FN1 is upregulated compared with normal tissues, and correlates with poor clinical outcomes [68]. Both FN1 and BGN correlate positively in a transcriptomic study between canine mammary carcinomas and human breast cancer [69]. Therefore, our findings support and strengthen the potential role for these proteins as CMT biomarkers in EVs.

In our isolation procedure we were unable to distinguish between exosomes and microvesicles. However, the EVs of the different cell lines had similar size distributions though varying amounts of CD9 and CD63 protein abundance. This might indicate that the isolated EVs contained different proportions of exosomes and MVs [70]. Nevertheless, the higher biglycan abundance in the carcinoma EVs was clearly not due to these differences as MTH53A EVs expressed similar CD9 levels as the complex carcinoma cell lines, but biglycan was hardly detectable (Additional file 10).

Apart from the obvious limitation that in vitro cell systems cannot represent the whole spectrum of CMTs, another major limitation in our study is that EV isolation from cell culture media only provides relatively low yields; therefore, it cannot be excluded that low-level proteins have not been detected. Moreover, using serum-free medium was exclusively chosen to avoid any possible contamination for proteomics application. We acknowledge this might affect the cellular response and therefore we cannot exclude that some proteins might be detected because of cell starvation. Another important limitation was the scarcity of canine proteomic data in the UniProtKB database as many canine proteins were not mapped. Nevertheless, our data suggests that subtyping disease stages from EVs proteins is feasible and that our proteomic analyses could now form the basis to develop biomarkers suitable for clinical diagnostics of canine mammary cancers. Follow-up studies will now investigate whether these CMT signatures can also be detected in serum samples from clinical patients and even identify CMT subtypes.

## Conclusions

Our study is the first to describe the proteomic profiles of WCLs and EVs from canine mammary tumour-derived cell lines. We found that WCLs can be used to distinguish different CMT phenotypes based on protein abundance, and that EVs derived from CMTs resemble the biological behaviour of their (parental) WCLs. Thus, EVs could potentially be used as diagnostic tools for detecting specific biomarkers of disease state in liquid biopsies, enabling the prediction of tumour development and progression in conjunction with conventional techniques. BGN was one of the most over-represented proteins in the carcinoma EV signature and shows potential as an EV-biomarker for canine mammary tumours. However, further potential markers, including FN1, require further verification. Equally, further studies with larger CMT subtype cohorts as well as patient material will be needed to experimentally validate these protein markers. Given the apparent similarities between canine mammary cancer and human breast cancer, it is possible that such data could also be valuable in the diagnosis of the human disease.

## Electronic supplementary material

Below is the link to the electronic supplementary material.

Supplementary Material 1

## Data Availability

The mass spectrometry proteomics data have been deposited to the ProteomeXchange Consortium via the PRIDE [71] partner repository with the dataset identifier PXD046033 and 10.6019/PXD046033. Reviewer account details: Username: reviewer_pxd046033@ebi.ac.uk. Password: dMG0adD3.

## Abbreviations

CMT: Canine mammary tumour
EVs: Extracellular vesicles
WCL: Whole-cell lysate
FBS: Foetal bovine serum
SEC: Size exclusion chromatography
NTA: Nanoparticle tracking analysis
LC-MS/MS: Liquid chromatography with tandem mass spectrometry
TMT: Tandem mass tag
TEAB: Triethylammonium bicarbonate
ACN: Acetonitrile
log2(FC): Log2-transformed fold change
WGCNA: Weighted gene correlation network analysis
GO: Gene ontology
FDR: False discovery rate
PCA: Principal component analysis
F.E: Fold enrichment
ECM: Extracellular matrix
BGN: Biglycan
