# Supplementary material for: Proteomic analysis of extracellular vesicles derived from canine mammary tumour cell lines identifies protein signatures specific for disease state
Source: BMC Vet Res. 2024 Oct 26;20:488. doi: 10.1186/s12917-024-04331-1 (PMC11515202; doi:10.1186/s12917-024-04331-1)
Supplement: Supplementary file 1 — Supplementary Material 1 [file 12917_2024_4331_MOESM1_ESM.pdf]

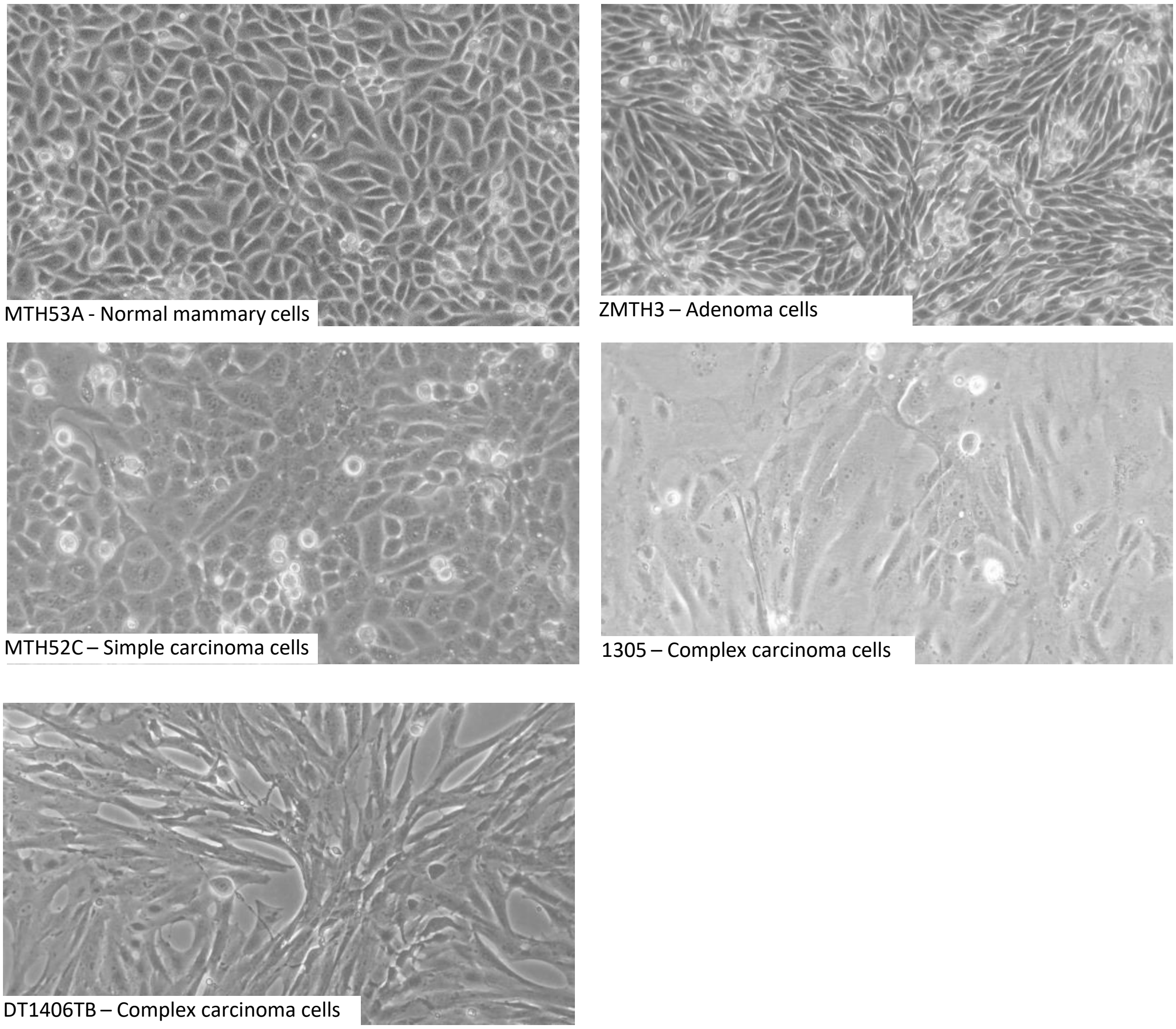

Additional file 1. Morphology of the canine mammary cell lines used in our study. While the cell lines MTH53A and MTH52C have the typical epithelial ‘cobblestone‘-appearance the complex carcinoma cell lines show an elongated morphology, with the adenoma cell line showing an intermediate growth pattern. Inverted microscopy; 100x magnification.

Additional file 2. Top 10 differentially over- and under-represented proteins identified in carcinoma WCLs vs healthy control

Over-represented proteins											
**MTH52C vs MTH53A**				**1305 vs MTH53A**				**DT1406TB vs MTH53A**			
**Accession**	**Protein**	**Log2FC**	**Adj. P-value**	**Accession**	**Protein**	**Log2FC**	**Adj.P-value**	**Accession**	**Protein**	**Log2FC**	**Adj. P-value**
A0A8I3P1M0	DCTPP1	9.513	7.55E-05	A0A8I3MKP5	SERPINB5	11.184	1.06E-06	A0A8I3RXX3	NDUFA4L2	9.627	4.33E-05
A0A8I3P452	CA3	8.004	1.57E-04	A0A8I3P342	ISG15	11.148	8.37E-07	A0A8I3PLN9	ADH4	7.849	7.71E-05
A0A8I3Q6P3	TPM4	7.882	8.06E-05	A0A8I3PI03	TSPAN8	10.741	5.71E-04	A0A8I3NAS7	OCIAD2	7.775	2.88E-03
A0A8I3Q497	TPM4	7.749	4.05E-05	A0A8I3MST9	DHRS2	10.636	3.69E-05	A0A8I3NCP2	ACSF2	7.730	9.20E-04
A0A8I3PIK8	AHSG	7.537	6.37E-05	A0A8I3MI64	SULT2B1	10.562	6.80E-06	A0A8I3N4I3	TPBG	7.631	2.77E-04
A0A8P0NQA3	PTMA	7.315	2.40E-05	A0A8I3RSK1	EPCAM	10.081	1.67E-05	A0A8P0TPH2	NN[b]	7.395	5.68E-06
A0A8I3Q8W7	NN[a]	7.170	1.92E-03	A0A8I3PTT4	MYLK	9.804	4.00E-06	A0A8I3N072	SULT1C4	7.128	1.35E-04
A0A8I3NGU2	LOC476816	6.698	1.09E-03	A0A8I3RRG1	HSPB6	9.503	2.74E-05	A0A8I3NXI7	CASP14	7.041	1.69E-03
A0A8I3PZB0	MDK	6.614	1.05E-04	A0A8I3PLN9	ADH4	9.464	1.40E-05	A0A8I3NW79	PTK2B	6.784	9.42E-04
A0A8P0S8Q7	RTN4	6.397	9.00E-06	J9NVI2	BST2	9.453	1.51E-05	A0A8I3PPA0	PTGIS	6.691	2.00E-06
**Under-represented proteins**											
**MTH52C vs MTH53A**				**1305 vs MTH53A**				**DT1406TB vs MTH53A**			
**Accession**	**Protein**	**Log2FC**	**Adj. P-value**	**Accession N°**	**Protein**	**Log2FC**	**Adj.P-value**	**Accession**	**Protein**	**Log2FC**	**Adj. P-value**
A0A8I3PDZ7	RTCB	-6.061	3.80E-05	A0A8I3SAW2	CHAF1B	-8.716	4.72E-06	A0A8I3PVE8	LOC119868340	-6.999	5.00E-04
A0A8I3PYS5	EIF4G1	-5.624	3.89E-04	A0A8I3N128	CDK2	-7.813	1.96E-06	A0A8I3RRM5	SLC7A5	-6.557	7.39E-04
A0A8I3N1X7	PLRG1	-5.528	1.40E-06	A0A8I3NQ76	CYP39A1	-7.234	6.89E-07	A0A8I3S5W2	MID1	-6.256	3.59E-04
A0A8I3NQ76	CYP39A1	-5.342	1.07E-06	A0A8I3MM38	FBL	-7.114	3.34E-05	A0A8I3P3E3	TGM3	-6.058	2.08E-04
A0A8I3RWC5	PLEKHG4	-5.073	4.79E-05	A0A8I3PVE8	LOC119868340	-7.090	4.17E-04	A0A8I3PDZ7	RTCB	-6.013	1.15E-03
A0A8P0STD7	NN[c]	-5.069	6.21E-03	A0A8I3Q3M7	CDC45	-7.065	4.95E-04	A0A8I3NSD1	CUBN	-5.952	4.36E-05
A0A8P0NNH4	TENM3	-4.901	1.33E-04	A0A8I3MU85	TIMM50	-6.760	7.95E-05	A0A8I3NZL8	CLDND1	-5.828	4.65E-03
A0A8I3S0T5	CEBPZ	-4.729	6.27E-05	A0A8P0SH46	CA5B	-6.755	1.88E-06	A0A8I3MVP4	ARFGEF3	-5.276	4.37E-05
A0A8I3PJ19	SMARCA4	-4.700	1.07E-05	A0A8I3RTJ1	PRIM2	-6.627	1.18E-05	A0A8P0PC40	USP9X	-5.276	1.65E-02
A0A8I3MKV2	LOC487150	-4.693	3.03E-04	A0A8I3S0M7	DPYD	-6.594	1.00E-06	A0A8I3NR12	CDKN2B	-5.176	1.37E-03

a: Prefoldin subunit 4; b: IF rod domain containing protein; c: 40S ribosomal protein S2

Additional file 3. Top over- and under-represented proteins in WCL- derived proteomes and their association with cancer, according to literature research

№	**Cell line**	**Sample type**	**Condition**	**Protein name**	**Protein abbreviation**	**Accession number**	**Association with cancer**
**1**	ZMTH3	WCL	Over-represented	Eukaryotic translation initiation factor 4C	EIF1AX	A0A8I3QIQ3	Significantly higher in human breast cancer [1], mutations related to malignancy in human thyroid cancer [2, 3].
**2**	ZMTH3	WCL	Over-represented	Acyl-CoA synthetase family member 2	ACSF2	A0A8I3NCP2	High expression levels in human hepatocellular carcinoma. May estimate prognosis of breast cancer patients [4].
**3**	ZMTH3	WCL	Over-represented	OCIA domain containing 2	OCIAD2	A0A8I3NAS7	Higher expression in human lung adenocarcinoma. Promotes survival of lung cancer cells [5].
**4**	ZMTH3	WCL	Over-represented	Tensin 3	TNS3	A0A8I3NH85	Downregulated in human kidney tumours. Correlated negatively with tumour grade, but not tumour size. Metastasis suppressor [6].
**5**	ZMTH3	WCL	Over-represented	ISG15 ubiquitin like modifier	ISG15	A0A8I3P342	Pro-tumoural and upregulated in melanoma and lung, breast, prostate, nasopharyngeal and oral cancers; tumour suppressor in several cancer types of cervix, blood, and ovaries [7].
**6**	ZMTH3	WCL	Over-represented	FAM83H protein	FAM83H	H6VX52	Downregulated in cutaneous squamous cell carcinoma (SCC) and correlated with differentiation grade. Overexpression led to decreases in cell migration and invasion [8]. Overexpression increased proliferation and invasion in human osteosarcoma, as well as tumour growth and expression of β-catenin [9]. Overexpressed in liver cancer cells and poor prognostic indicator of human hepatocellular carcinoma [10].
**7**	ZMTH3	WCL	Over-represented	Peptidyl-prolyl cis-trans isomerase	PPIC	A0A8I3N5K4	Upregulated in human glioma tissue [11].
**8**	ZMTH3	WCL	Over-represented	Cysteine and glycine rich protein 1	CSRP1	A0A8I3P8T5	Overexpressed in human colon adenocarcinoma in more advanced tumour stages and worst prognosis [12].
**9**	ZMTH3	WCL	Under-represented	Cytochrome P450 family 39 subfamily A member 1	CYP39A1	A0A8I3NQ76	Downregulation is associated with human hepatocellular carcinoma carcinogenesis, tumour differentiation and poor overall survival [13].
**10**	ZMTH3	WCL	Under-represented	Calcyphosin	CAPS	P10463	Higher levels of CAPS in human oesophageal squamous cell carcinoma than non-cancerous samples. Positively associated with histological grade and shorter overall survival time [14].
**11**	ZMTH3	WCL	Under-represented	Dermatopontin	DPT	A0A8I3RS96	Under-expressed in most human colorectal cancer samples [15]. Downregulated in oral squamous cell carcinoma and correlated with cell adhesion and

№	Cell line	Sample type	Condition	Protein name	Protein abbreviation	Accession number	Association with cancer
							invasiveness [16]. Downregulated in human hepatocellular carcinoma [17].
**12**	ZMTH3	WCL	Under-represented	Dedicator of cytokinesis 4	DOCK4	A0A8I3SCL9	Overexpressed in human stomach adenocarcinoma, with negative impact on prognosis. Associated with tumour immune infiltration [18]. Overexpression in early human breast cancer is associated with aggressive disease and future bone metastasis [19].
**13**	ZMTH3	WCL	Under-represented	creatine kinase	LOC478277	A0A8I3S958	Poor prognostic factor in overall survival of endometrial cancer patients [20].
**14**	ZMTH3	WCL	Under-represented	Thioredoxin interacting protein	TXNIP	A0A8I3NKV1	Expressed in tumour from various cancer cells, including breast, colorectal, lung and liver cancer [21].
**15**	ZMTH3	WCL	Under-represented	Creatine kinase B-type	CKB	P05124	Downregulation is a poor prognosis marker in prostate cancer [22]. Overexpression has a protective effect in colorectal cells [23].
**16**	MTH52C	WCL	Over-represented	dCTP pyrophosphatase 1	DCTPP1	A0A8I3P1M0	Elevated levels correlate with poor breast cancer prognosis [24, 25].
**17**	MTH52C	WCL	Over-represented	Carbonic anhydrase	CA3	A0A8I3P452	Overexpression associated with cancer progression in oral squamous cell carcinoma [26].
**18**	MTH52C	WCL	Over-represented	RAB8A, member RAS onco family	TPM4	A0A8I3Q6P3	Upregulated in lung cancer. Potential marker of ovarian cancer, breast cancer, colon cancer, keratoacanthoma and oesophageal squamous cell carcinoma [27].
**19**	MTH52C	WCL	Over-represented	RAB8A, member RAS onco family	TPM4	A0A8I3Q497	Described above (nº 18).
**20**	MTH52C	WCL	Over-represented	Alpha-2-HS-glycoprotein	AHSG	A0A8I3PIK8	High serum levels in gastric cancer patients with favourable diagnostic values; associated with tumour stage [28] Contributes to tumour progression. Its presence in tumour microenvironment may also promote efficient synthesis, secretion and endocytic uptake of exosomes, promoting tumour growth [29].
**21**	MTH52C	WCL	Over-represented	Prothymosin alpha	PTMA	A0A8P0NQA3	Upregulated in oesophageal squamous cell carcinoma tissue [30] and bladder cancer tissue [31].
**22**	MTH52C	WCL	Over-represented	Prefoldin subunit 4	3 SV	A0A8I3Q8W7	Overexpression is related to better prognosis in colorectal cancer [32].
**23**	MTH52C	WCL	Over-represented	Folate_rec domain-containing protein	LOC476816	A0A8I3NGU2	Overexpressed in ovarian carcinoma in higher grade than breast cancer effusion samples [33].
**24**	MTH52C	WCL	Over-represented	Midkine	MDK	A0A8I3PZB0	Overexpression correlated with malignancy in non-small cell lung cancer [34, 35], pancreatic cancer, bladder cancer, melanoma, brain tumours, oesophageal cancer, breast cancer and ovarian cancer [36].

Nº	Cell line	Sample type	Condition	Protein name	Protein abbreviation	Accession number	Association with cancer
**25**	MTH52C	WCL	Over-represented	Reticulon	RTN4	A0A8P0S8Q7	Overexpression associated with poor survival in lung, breast, cervical and renal cancer [37].
**26**	MTH52C	WCL	Under-represented	RNA-splicing ligase RtcB homolog	RTCB	A0A8I3PDZ7	Inhibition improves response to therapy in antioestrogen-resistant breast cancer cells [38].
**27**	MTH52C	WCL	Under-represented	Eukaryotic translation initiation factor 4 gamma 1	EIF4G1	A0A8I3PYS5	Overexpression in breast cancer is associated with cell survival and DNA damage response, preventing autophagy and apoptosis [39]. Overexpression in lung cancer had immunoregulatory functions [40].
**28**	MTH52C	WCL	Under-represented	Cytochrome P450 family 39 subfamily A member 1	CYP39A1	A0A8I3NQ76	Described above (nº 9).
**29**	MTH52C	WCL	Under-represented	Pleckstrin homology and RhoGEF domain containing G4	PLEKHG4	A0A8I3RWC5	Upregulated in human thyroid cancer tissue. Loss of PLEKHG4 enhanced apoptosis ability and stemness properties [41].
**30**	MTH52C	WCL	Under-represented	40S ribosomal protein S2	3 SV	A0A8P0STD7	Overexpression activate oncogenes involved in tumour development [42].
**31**	MTH52C	WCL	Under-represented	Teneurin transmembrane protein 3	TENM3	A0A8P0NNH4	Overexpressed in head and neck squamous cell carcinoma, pancreatic adenocarcinoma, thymoma and neuroblastoma. Upregulated in breast and colorectal cancer with metastatic potential. Downregulation associated with poor prognosis in cervical, pancreatic and renal cancer [43].
**32**	MTH52C	WCL	Under-represented	SWI/SNF related, matrix associated, actin dependent regulator of chromatin, subfamily a, member 4	SMARCA4	A0A8I3PJ19	Overexpression is associated with poor prognosis in many types of tumours, including liver and renal cancer. Silenced or mutated in many cancer cells [44].
**33**	MTH52C	WCL	Under-represented	Malignant T-cell-amplified sequence	LOC487150	A0A8I3MKV2	Overexpression is associated with malignancy in human breast cancer, lung cancer, oral cancer and mesothelioma [45-47].
**34**	1305	WCL	Over-represented	Serpin B5	SERPINB5	A0A8I3MKP5	Tumour suppressor activity in breast and prostatic cancers, but tumour progressive features in colorectal cancer [48].
**35**	1305	WCL	Over-represented	ISG15 ubiquitin like modifier	ISG15	A0A8I3P342	Described above (nº 5).
**36**	1305	WCL	Over-represented	Tetraspanin	TSPAN8	A0A8I3PI03	Overexpressed and associated with poor prognosis in pancreatic cancer, colon cancer, gastric cancer, liver cancer, lung cancer, breast cancer, ovarian cancer, glioma, melanoma, oesophageal cancer, nasopharyngeal cancer, cancer stem cells [49]. Increases extracellular vesicles release [50].
**37**	1305	WCL	Over-represented	Dehydrogenase/reductase 2	DHRS2	A0A8I3MST9	Downregulated in oesophageal squamous cell carcinoma and ovarian cancer [51]. Tumour suppressor activity.
**38**	1305	WCL	Over-represented	Sulfotransferase	SULT2B1	A0A8I3MI64	Overexpressed in colorectal cancer tissue [52] and cervical cancer [53]. Tumour progression activity.

№	Cell line	Sample type	Condition	Protein name	Protein abbreviation	Accession number	Association with cancer
**39**	1305	WCL	Over-represented	Epithelial cell adhesion molecule	EPCAM	A0A8I3RSK1	Upregulated in solid epithelial cancers and stems cells. Can also be found in disseminated tumour cells and circulating tumour cells [54].
**40**	1305	WCL	Over-represented	Myosin light chain kinase, smooth muscle	MYLK	A0A8I3PTT4	Upregulated in breast cancer cells, responsible of high proliferative ability with metastatic potential [55].
**41**	1305	WCL	Over-represented	Heat shock protein family B (small) member 6	HSPB6	A0A8I3RRG1	Overexpressed in lung cancer. Tumour progression protein [56].
**42**	1305	WCL	Over-represented	Alcohol dehydrogenase 4 (class II), pi polypeptide	ADH4	A0A8I3PLN9	Low expression in hepatocellular carcinoma vs normal tissue. Tumour suppressor protein associated with worse overall survival rate [57].
**43**	1305	WCL	Over-represented	Bone marrow stromal antigen 2 type 2	BST2	J9NVI2	Overexpression associated with bone metastasis formation in human breast cancer [58]. Associated with poor survival in oesophageal, gastric and colorectal cancer [59].
**44**	1305	WCL	Under-represented	Chromatin assembly factor 1 subunit B	CHAF1B	A0A8I3SAW2	Tumour progression protein in lung cancer [60]. Overexpression associated with poor prognosis in melanoma, prostate cancer, salivary gland tumours, nasopharyngeal cancer, breast cancer, high grade glioma and hepatocellular carcinoma [60].
**45**	1305	WCL	Under-represented	Cyclin dependent kinase 2	CDK2	A0A8I3N128	Overexpressed with oncogenic activity in ovarian cancer, hepatocellular carcinoma, glioblastoma, prostate cancer, and B cell lymphoma [61].
**46**	1305	WCL	Under-represented	Cytochrome P450 family 39 subfamily A member 1	CYP39A1	A0A8I3NQ76	Described above (nº 9).
**47**	1305	WCL	Under-represented	Fibrillarin	FBL	A0A8I3MM38	Overexpression associated with tumour progression in hepatocellular carcinoma [62]. Low expression associated with poor outcome in breast cancer [63].
**48**	1305	WCL	Under-represented	MAGE domain-containing protein	LOC119868340	A0A8I3PVE8	Oncogenic activity through control of p53 tumour suppressor [64].
**49**	1305	WCL	Under-represented	Cell division cycle 45	CDC45	A0A8I3Q3M7	Overexpressed in human cancer-derived cells (carcinoma-, sarcoma-, leukaemia-, and lymphoma-derived cells [65], as well as cervical cancer tissue [66].
**50**	1305	WCL	Under-represented	Mitochondrial import inner membrane translocase subunit TIM50	TIMM50	A0A8I3MU85	Oncogenic protein overexpressed in lung cancer [67] and breast cancer [68].
**51**	1305	WCL	Under-represented	Carbonic anhydrase	CA5B	A0A8P0SH46	CA5B mRNA upregulation in acute myeloid leukaemia, prostate and renal cell carcinomas [69].
**52**	1305	WCL	Under-represented	DNA primase large subunit	PRIM2	A0A8I3RTJ1	Overexpressed and associated with poor prognosis in lung cancer [70].
**53**	1305	WCL	Under-represented	Dihydropyrimidine dehydrogenase [NADP(+)]	DPYD	A0A8I3S0M7	Low expression of DPD mRNA in colorectal tumours, and colorectal liver metastasis [71].
**54**	DT1406TB	WCL	Over-represented	NDUFA4 mitochondrial complex associated like 2	NDUFA4L2	A0A8I3RXX3	Overexpressed in lung cancer [72], glioblastoma [73] and colorectal cancer [74].
**55**	DT1406TB	WCL	Over-represented	Alcohol dehydrogenase 4 (class II), pi polypeptide	ADH4	A0A8I3PLN9	Described above (nº 42).

Nº	Cell line	Sample type	Condition	Protein name	Protein abbreviation	Accession number	Association with cancer
**56**	DT1406TB	WCL	Over-represented	OCIA domain containing 2	OCIAD2	A0A8I3NAS7	Described above (nº 3).
**57**	DT1406TB	WCL	Over-represented	Acyl-CoA synthetase family member 2	ACSF2	A0A8I3NCP2	Described above (nº 2).
**58**	DT1406TB	WCL	Over-represented	Trophoblast glycoprotein	TPBG	A0A8I3N4I3	Overexpressed in lung cancer and gastric cancer, associated with poor prognosis [75].
**59**	DT1406TB	WCL	Over-represented	Caspase 14	CASP14	A0A8I3NXI7	Overexpressed in breast cancer. Marker of aggressiveness mainly associated with triple negative phenotypes and stemness [76].
**60**	DT1406TB	WCL	Over-represented	non-specific protein-tyrosine kinase	PTK2B	A0A8I3NW79	Overexpression correlates with reduced survival in pure HER2 breast cancer patients [77].
**61**	DT1406TB	WCL	Over-represented	Prostacyclin synthase	PTGIS	A0A8I3PPA0	Overexpression associated with poor overall survival in lung, ovarian and gastric cancer. Metastasis predictor [78]. Predictive marker of ovarian cancer [79].
**62**	DT1406TB	WCL	Under-represented	MAGE domain-containing protein	LOC119868340	A0A8I3PVE8	Described above (nº 48).
**63**	DT1406TB	WCL	Under-represented	Solute carrier family 7 member 5	SLC7A5	A0A8I3RRM5	Overexpressed in human breast cancer, and correlated with larger tumour size and higher grade [80].
**64**	DT1406TB	WCL	Under-represented	E3 ubiquitin-protein ligase Midline-1	MID1	A0A8I3S5W2	Low expression in many cancers, including colon and breast cancer, associated with high invasion and metastasis [81].
**65**	DT1406TB	WCL	Under-represented	Transglutaminase 3	TGM3	A0A8I3P3E3	Upregulated in some tumour tissues like hepatocellular carcinoma, and downregulated in other epithelial carcinomas such as laryngeal carcinoma and oesophageal carcinoma, in which high expression indicates poor prognosis [82]. Candidate tumour suppressor gene in human head and neck cancer [83].
**66**	DT1406TB	WCL	Under-represented	RNA-splicing ligase RtcB homolog	RTCB	A0A8I3PDZ7	Described above (nº 26).
**67**	DT1406TB	WCL	Under-represented	Cubilin	CUBN	A0A8I3NSD1	Lower expression is correlated with poor prognosis in renal cell carcinoma. Overexpressed in colorectal cancer [84].
**68**	DT1406TB	WCL	Under-represented	Claudin domain containing 1	CLDND1	A0A8I3NZL8	Downregulation in breast cancer induces apoptosis caspase-dependent [85].
**69**	DT1406TB	WCL	Under-represented	ARFGEF family member 3	ARFGEF3	A0A8I3MVP4	Overexpressed in the majority of breast cancers. Knockdown has tumour suppression effect [86].
**70**	DT1406TB	WCL	Under-represented	ubiquitinyl hydrolase 1	USP9X	A0A8P0PC40	Upregulated in liver cancer cells [87]. Downregulated in cholangiocarcinoma, which contributes to tumorigenesis [88].

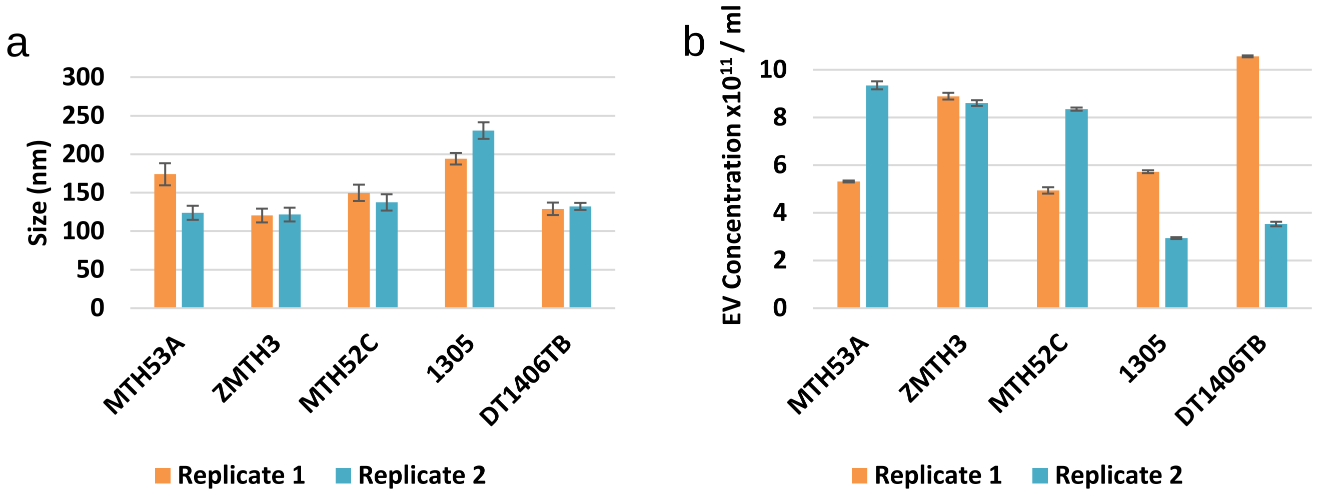

Additional file 4. Characterisation of EVs obtained from size exclusion chromatography by nanoparticle tracking analysis (NTA) **a)** Mean particle size (nm) **b)** Concentration of EVs. The graphs show the typical mean particle sizes of extracellular vesicles (EV) with concentrations ranging from 3 to 10 x 10^{11} particles per ml in the two replicate isolates.

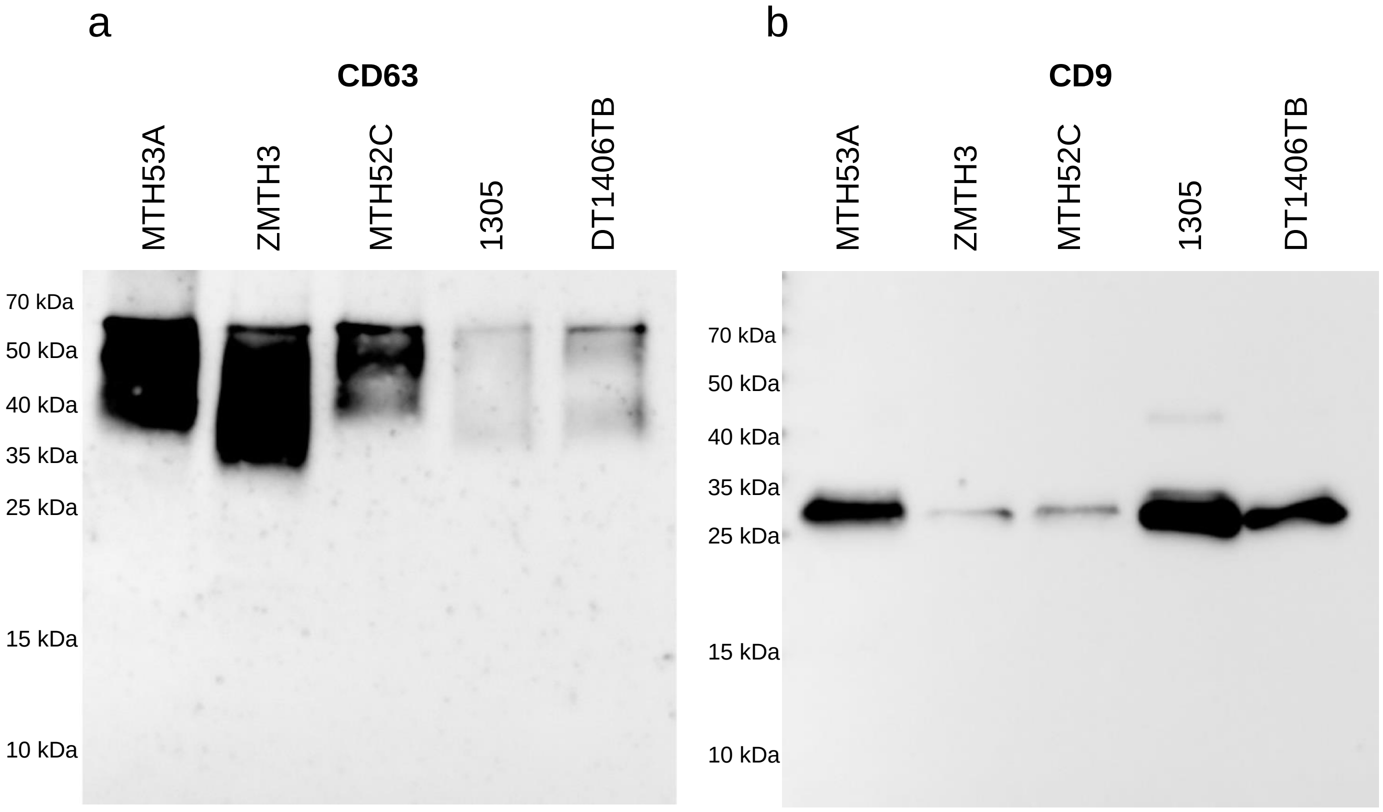

Additional file 5. Western blot for EV markers **a)** CD63 and **b)** CD9 using protein extracts from the isolated EVs. The filter was incubated first with an anti-CD9 and subsequently with an anti-CD63 antibody after mild stripping. The images show the typical smeary pattern for CD63 between 35 and 63kDa and a single ~26kDa band for CD9, with variable expression of the two markers. Images were acquired after 5 minutes of exposure using a Fusion imaging system.

Additional file 6. Top 10 differentially over- and under-represented proteins identified in carcinoma EVs vs healthy control

Over-represented proteins

MTH52C vs MTH53A				1305 vs MTH53A				DT1406TB vs MTH53A			
**Accession**	**Protein**	**Log2FC**	**Adj. P-value**	**Accession**	**Protein**	**Log2FC**	**Adj.P-value**	**Accession**	**Protein**	**Log2FC**	**Adj. P-value**
A0A8I3P684	LUM	11.155	2.26E-03	A0A8I3MHG8	MRPL18	15.114	7.19E-06	A0A8I3P5P0	ECM1	13.144	4.73E-07
A0A8P0TLW8	SSC5D	8.289	3.37E-02	A0A8I3NTB5	CANT1	12.760	3.62E-07	A0A8I3PAS0	HAPLN1	11.965	3.31E-06
A0A8I3NGU2	LOC476816	7.242	3.78E-03	A0A8I3QWY5	LIPH	12.233	1.65E-07	Q28275	FN1	10.712	4.68E-07
Q6QNF3	PDGFRB	7.129	1.77E-02	A0A8I3Q5H9	A2M	12.219	4.89E-07	A0A8I3PUQ0	SBSPON	10.018	1.59E-04
A0A8I3NPK1	FSCN1	6.969	1.37E-03	J9NVI2	BST2	11.935	6.70E-05	A0A8I3S4S1	IGFBP6	9.869	1.44E-06
A0A8I3PAS0	HAPLN1	6.534	4.35E-04	A0A8I3RZF7	PSMB10	11.264	6.31E-06	A0A8P0NSQ8	IGFBP6	9.728	3.64E-05
A0A8I3NL35	COL12A1	6.405	2.30E-06	A0A8I3PZB0	MDK	11.262	2.35E-05	A8QWU1	SLPI	9.622	2.12E-05
A0A8I3PBE9	SPON1	6.124	1.76E-04	A0A8I3RVN0	COL14A1	11.132	4.31E-05	A0A8P0TAG1	PACSIN1	9.461	9.47E-06
A0A8P0TJJ1	RELN	6.078	3.90E-02	A0A8P0TLW8	SSC5D	10.690	2.38E-07	A0A8I3Q308	OLFML2B	9.408	4.33E-06
A0A8I3NZ42	CLEC11A	5.667	1.58E-02	A0A8I3S3F7	GPRC5C	10.618	1.75E-05	A0A8I3NGU2	LOC476816	9.315	1.65E-07

Under-represented proteins

MTH52C vs MTH53A				1305 vs MTH53A				DT1406TB vs MTH53A			
**Accession**	**Protein**	**Log2FC**	**Adj. P-value**	**Accession**	**Protein**	**Log2FC**	**Adj.P-value**	**Accession**	**Protein**	**Log2FC**	**Adj. P-value**
A0A8P0SLL9	XPO6	-5.010	2.94E-04	Q9XSJ7	COL1A1	-8.277	7.62E-05	A0A8I3PHH6	QPCT	-8.963	4.74E-05
A0A8I3PRZ8	NRCAM	-4.151	4.25E-02	A0A8I3PI61	SLX9	-8.153	4.61E-05	A0A8I3RZY0	LETM1	-7.998	1.42E-04
A0A8I3NRJ5	ELAVL2	-4.016	2.12E-02	A0A8I3QI05	SORD	-8.099	3.46E-04	A0A8P0SUA9	TNN	-7.259	5.05E-05
A0A8I3PMJ4	DIS3L2	-3.638	5.07E-03	A0A8I3P795	SNU13	-7.794	1.15E-03	A0A8I3PX13	H3C4	-7.153	4.04E-05
A0A8I3PCK9	FAT1	-3.429	1.78E-02	A0A8P0SLL9	XPO6	-7.770	2.03E-04	A0A8P0SB44	SARS2	-7.035	4.28E-04
A0A8I3S8V0	CCN1	-3.353	1.53E-02	A0A8I3PTI4	TRMT5	-7.309	1.09E-05	Q9XSJ7	COL1A1	-6.740	4.46E-03
A0A8P0NLM5	ACADM	-3.349	3.71E-02	A0A8P0S7F1	HMGA1	-7.216	1.76E-03	A0A8P0SLL9	XPO6	-6.686	4.33E-06
D2YYC0	Madh3	-3.338	1.78E-02	A0A8P0PAW3	CRTAC1	-6.648	1.81E-04	A0A8P0SQL8	3 SV[b]	-6.671	1.34E-03
A0A8I3RUE8	HGH1	-3.327	3.95E-02	A0A8P0SLA8	COL1A1	-6.606	2.87E-04	E2R6K5	H3-3A	-6.592	1.48E-03
A0A8I3NJ93	AARS2	-3.229	2.07E-02	A0A8I3MER3	4 SV[a]	-6.426	2.29E-04	A0A8P0SET5	WDR3	-6.228	1.94E-05

a: Rad60-SLD domain-containing protein, b: Ribosomal protein L37a.

Additional file 7. Top over- and under-represented proteins in EV- derived proteomes and their association with cancer, according to literature research

Nº	Cell line	Sample type	Condition	Protein name	Protein abbreviation	Accession number	Association with cancer
**71**	ZMTH3	EV	Over-represented	CTP synthase	CTPS2	A0A8I3QAX6	Overexpressed in chronic lymphocytic leukaemia, associated with poor prognosis [89].
**72**	ZMTH3	EV	Over-represented	Galectin-3-binding protein	CANT1	A0A8I3NTB5	Overexpressed in lung cancer, renal cell carcinoma [90, 91] and prostate cancer [92].
**73**	ZMTH3	EV	Over-represented	Transcobalamin 2	TCN2	A0A8I3PAV5	Low expression has an effect similar to hypoxia. High expression can reverse it [93]. Overexpressed in canine and feline tumour tissues [94].
**74**	ZMTH3	EV	Over-represented	ISG15 ubiquitin like modifier	ISG15	A0A8I3P342	Described above (nº 5).
**75**	ZMTH3	EV	Over-represented	Microfibril associated protein 2	MFAP2	A0A8I3MLA0	Overexpressed in bladder cancer, brain and central nervous system cancer, breast cancer, colorectal cancer, oesophageal cancer, gastric cancer, head and neck cancer, lymphoma, melanoma, myeloma, ovarian cancer, pancreatic cancer, sarcoma [95] and hepatocellular carcinoma [96]. Associated with poor prognosis.
**76**	ZMTH3	EV	Over-represented	ArfGAP with dual PH domains 1	ADAP1	A0A8P0TVC6	Promotes invasive squamous cell carcinoma progression [97] and metastatic colorectal cancer [98].
**77**	ZMTH3	EV	Over-represented	Zona pellucida like domain containing 1	ZPLD1	A0A8I3PFH2	Associated with pancreatic cancer and breast cancer metastasis [99].
**78**	ZMTH3	EV	Over-represented	Bone marrow stromal antigen 2 type 2	BST2	J9NVI2	Described above (nº 43).
**79**	ZMTH3	EV	Over-represented	Interferon induced protein 44	IFI44	A0A8I3MKR1	Correlated with immune infiltration in head and neck squamous cell carcinoma [100].
**80**	ZMTH3	EV	Over-represented	Serine protease 23	PRSS23	A0A8P0N757	Upregulated in breast cancer cells, prostate cancer, papillary thyroid cancer, pancreatic cancer [101] and gastric cancer [102].
**81**	ZMTH3	EV	Under-represented	Matrix remodeling-associated protein 8	MXRA8	A0A8I3PNQ7	Cancer-associated marker in pancreatic cancer [103]. Expression correlated with malignancy, metastasis, recurrence and immunosuppressive microenvironment [104].
**82**	ZMTH3	EV	Under-represented	Angiopoietin like 2	ANGPTL2	A0A8I3N7R9	Tumourigenic role in thyroid cancer cells [105]. Overexpressed in lung cancer, colorectal cancer, prostate cancer and gastric cancer [106]. Dual function (tumour progression/anti tumour immunity) [107].
**83**	ZMTH3	EV	Under-represented	RAB8A, member RAS onco family	TPM4	A0A8I3Q6P3	Described above (nº 18).
**84**	ZMTH3	EV	Under-represented	Tenascin N	TNN	A0A8I3NDQ9	Overexpressed in high grade tumours [108]. Novel marker for tumour stroma in low-grade human

№	Cell line	Sample type	Condition	Protein name	Protein abbreviation	Accession number	Association with cancer
							breast cancer [109]. Elevated in serum of colon and breast cancer [110].
**85**	ZMTH3	EV	Under-represented	RAB8A, member RAS onco family	TPM4	A0A8I3Q497	Described above (nº 18).
**86**	ZMTH3	EV	Under-represented	LamGL domain-containing protein	PTX3	A0A8I3PGJ4	Dual role. Low expression increases susceptibility to mesenchymal and epithelial carcinogenesis. Expression has anti-tumour activity in oesophageal squamous cell carcinoma and colorectal cancer. Pro-tumorigenic role in head and neck tumours, cervical cancer, glioma and hepatocellular carcinoma. Oncogenic protein in breast cancer [111].
**87**	ZMTH3	EV	Under-represented	NADH dehydrogenase [ubiquinone] 1 beta subcomplex subunit 8, mitochondrial	NDUFB8	A0A8I3NUH5	Low expression in tumour tissues in breast cancer [112].
**88**	ZMTH3	EV	Under-represented	Plasma retinol-binding protein	RBP4	A0A8P0T552	Downregulation indicates poor prognosis in hepatocellular carcinoma [113]. Associated with advanced tumour stages and grades. Overexpressed in glioblastoma with tumourigenic activity [114].
**89**	MTH52C	EV	Over-represented	Lumican	LUM	A0A8I3P684	Oncogene in gastric cancer progression. Correlated with poor overall survival. More frequently detected in carcinoma than adenoma cells [115].
**90**	MTH52C	EV	Over-represented	Folate_rec domain-containing protein	LOC476816	A0A8I3NGU2	Described above (nº 23).
**91**	MTH52C	EV	Over-represented	Platelet-derived growth factor receptor beta	PDGFRB	Q6QNF3	Overexpressed in metastatic medulloblastoma [116]. High expression in glioblastoma [117].
**92**	MTH52C	EV	Over-represented	Fascin	FSCN1	A0A8I3NPK1	Overexpressed in multiple cancers such as laryngeal squamous cell carcinoma, lung cancer, oral squamous cell carcinoma, oesophageal squamous cell carcinoma, gastric cancer, hepatocellular carcinoma, colorectal cancer, renal cell carcinoma, urothelial carcinoma, breast cancer and uterine carcinosarcoma [118].
**93**	MTH52C	EV	Over-represented	Hyaluronan and proteoglycan link protein 1	HAPLN1	A0A8I3PAS0	Overexpressed in pancreatic ductal adenocarcinoma and promotes metastasis [119]. Overexpressed in breast cancer [120], hepatocellular carcinoma [121] and mesothelioma [122]. In gastric cancer, cancer-associated fibroblasts promote tumour invasion through extracellular matrix remodelling [123]. In canine mammary tumours, a significant association between HAPLN1 with early stage canine malignant mammary tumour was reported [124].
**94**	MTH52C	EV	Over-represented	Collagen type XII alpha 1 chain	COL12A1	A0A8I3NL35	Upregulated in gastric cancer. Elevated expression correlated with invasiveness, metastasis and advanced clinical stage [125]. Upregulated in breast cancer tissues [126].

№	Cell line	Sample type	Condition	Protein name	Protein abbreviation	Accession number	Association with cancer
**95**	MTH52C	EV	Over-represented	Spondin-1	SPON1	A0A8I3PBE9	Overexpressed in ovarian cancer [127] and pancreatic cancer [128].
**96**	MTH52C	EV	Over-represented	Reelin	RELN	A0A8P0TJJ1	Silenced in glioblastoma as compared to non-neoplastic tissue [129]. Loss of reelin is associated to poor outcome [130]. Expressed in prostate cancer but not in benign prostate tissue [131].
**97**	MTH52C	EV	Over-represented	C-type lectin domain containing 11A	CLEC11A	A0A8I3NZ42	High expression predicts favourable prognosis in acute myeloid leukaemia [132]. Associated with development of leukaemia, multiple myeloma, and gastrointestinal tumours [133].
**98**	MTH52C	EV	Under-represented	Exportin 6	XPO6	A0A8P0SLL9	Upregulation acts as a tumour suppressor in breast cancer [134]. Highly expressed in prostate cancer tissues, with pro-tumour activity [135].
**99**	MTH52C	EV	Under-represented	Neuronal cell adhesion molecule	NRCAM	A0A8I3PRZ8	Overexpressed in papillary thyroid carcinomas, both in mRNA and protein levels [136]. Overexpressed in high-grade astrocytoma, glioma and glioblastoma tumour tissues; colon cancer, advanced stages of melanoma Low levels observed in neuroblastoma, meningioma, pancreatic adenocarcinoma [137]. Lower expression in gastric cancer tissues in contrast with normal tissues. High NRCAM expression indicates poor prognosis and promotes tumour progression [138].
**100**	MTH52C	EV	Under-represented	ELAV-like protein	ELAVL2	A0A8I3NRJ5	Tumour suppressor activity. Low expression is associated with cancer progression and chemoresistance in glioblastoma [139].
**101**	MTH52C	EV	Under-represented	DIS3-like exonuclease 2	DIS3L2	A0A8I3PMJ4	Loss of DIS3L2 is related with tumour progression in both *Drosophila* and human cells [140].
**102**	MTH52C	EV	Under-represented	FAT atypical cadherin 1	FAT1	A0A8I3PCK9	Upregulated in acute leukaemia, hepatocellular carcinoma, glioblastoma, and gastric cancer. Downregulated in head and neck squamous cell carcinoma, oesophageal squamous cell carcinoma, breast cancer and cervical cancer [141]. Loss of FAT1 and β-catenin are associated with breast cancer progression, aggressive behaviour and poor prognosis [142].
**103**	MTH52C	EV	Under-represented	Cellular communication network factor 1	CCN1	A0A8I3S8V0	Upregulated in prostate, ovarian , endometrial, and pancreatic cancer cells [143]. CCN1 expression promote metastasis and tumour progression in triple negative breast cancer, whereas low expression has tumour suppressor activity [144].
**104**	MTH52C	EV	Under-represented	Medium-chain specific acyl-CoA dehydrogenase, mitochondrial	ACADM	A0A8P0NLM5	Low expression in hepatocellular carcinoma, correlating with aggressive clinicopathologic

Nº	Cell line	Sample type	Condition	Protein name	Protein abbreviation	Accession number	Association with cancer
							features in patients [145]. Overexpressed in breast cancer tissues, acting as an oncogene [146].
**105**	MTH52C	EV	Under-represented	Mothers against decapentaplegic homolog	Madh3	D2YYC0	MADH3 expression may have a critical role in tumour suppression in early stages of gastric carcinogenesis. Loss of expression increases susceptibility to tumorigenicity in human gastric cancer [147].
**106**	MTH52C	EV	Under-represented	Protein HGH1 homolog	HGH1	A0A8I3RUE8	Upregulated in colon adenocarcinoma and rectum adenocarcinoma [148].
**107**	MTH52C	EV	Under-represented	Alanine--tRNA ligase	AARS2	A0A8I3NJ93	Loss of AARS decreases tumour progression of colon cancer cells [149].
**108**	1305	EV	Over-represented	Mitochondrial ribosomal protein L18	MRPL18	A0A8I3MHG8	Dysregulated expression of MRPL18 has been correlated with tumour progression in lung cancer [150].
**109**	1305	EV	Over-represented	Galectin-3-binding protein	CANT1	A0A8I3NTB5	Described above (nº 72).
**110**	1305	EV	Over-represented	Lipase H	LIPH	A0A8I3QWY5	Upregulated mRNA and protein expression in breast cancer tumours, and might be related to worse prognosis [151]. In pancreatic cancer, LIPH is considered a novel unfavourable prognostic marker [152].
**111**	1305	EV	Over-represented	Alpha-2-macroglobulin	A2M	A0A8I3Q5H9	Expression inhibits growth of tumours in mice [153]. Low expression in intrahepatic cholangiocarcinoma was an adverse prognostic factor [154].
**112**	1305	EV	Over-represented	Bone marrow stromal antigen 2 type 2	BST2	J9NVI2	Described above (nº 43).
**113**	1305	EV	Over-represented	Proteasome subunit beta	PSMB10	A0A8I3RZF7	Upregulated in breast cancer, affecting the efficacy of neoadjuvant chemotherapy [155].
**114**	1305	EV	Over-represented	Midkine	MDK	A0A8I3PZB0	Described above (nº 24).
**115**	1305	EV	Over-represented	Collagen type XIV alpha 1 chain	COL14A1	A0A8I3RVN0	Downregulation associated with aggressive breast cancer subtypes [156]. Overexpressed in liver cancer stem cells [157], and gastric cancer with peritoneal metastasis [158].
**116**	1305	EV	Over-represented	G protein-coupled receptor class C group 5 member C	GPRC5C	A0A8I3S3F7	Detected in extracellular vesicles from serum of pancreatic ductal adenocarcinoma; candidate biomarker for early pancreatic cancer [159].
**117**	1305	EV	Under-represented	Collagen alpha-1(I) chain	COL1A1	Q9XSJ7	Increased levels are associated with poor survival and metastasis status in breast cancer [160], colorectal cancer [161], and lung cancer [162]. Knockdown of COL1A1 inhibits metastasis.
**118**	1305	EV	Under-represented	Sorbitol dehydrogenase	SORD	A0A8I3QI05	Upregulated in colorectal adenomas and cancer cell lines [163]. In liver cancer, SORD high expression is associated with favourable effects, playing a functional role in hepatocellular suppression [164].

Nº	Cell line	Sample type	Condition	Protein name	Protein abbreviation	Accession number	Association with cancer
**119**	1305	EV	Under-represented	Ribonucleoprotein	SNU13	A0A8I3P795	Upregulated in breast cancer tumour samples and negatively correlated with prognosis [165].
**120**	1305	EV	Under-represented	Exportin 6	XPO6	A0A8P0SLL9	Described above (nº 98).
**121**	1305	EV	Under-represented	tRNA (guanine(37)-N1)-methyltransferase	TRMT5	A0A8I3PTI4	Upregulated in hepatocellular carcinoma and correlated with poor prognosis [166].
**122**	1305	EV	Under-represented	High mobility group protein HMG-I/HMG-Y	HMGA1	A0A8P0S7F1	Participates in tumourigenesis and tumour progression. Upregulated in many different tumours including epithelial and mesenchymal tissue-originated tumours. Overexpression is correlated with poor clinical outcome, distant metastasis and advanced tumour stage [167].
**123**	1305	EV	Under-represented	Cartilage acidic protein 1	CRTAC1	A0A8P0PAW3	Overexpression inhibits tumour progression in bladder cancer, while low expression correlates with poor survival [168].
**124**	1305	EV	Under-represented	Collagen alpha-1(I) chain	COL1A1	A0A8P0SLA8	Described above (nº 117).
**125**	DT1406TB	EV	Over-represented	Extracellular matrix protein 1	ECM1	A0A8I3P5P0	Overexpressed in colorectal cancer, bladder cancer, glioblastoma, thyroid cancer, cholangiocarcinoma, and other epithelial malignancies, and it is related to poor prognosis in pancreatic cancer, breast cancer, gastric cancer and hepatocellular cancer [169, 170]. Associated with tumour progression and cancer stem cell maintenance [171].
**126**	DT1406TB	EV	Over-represented	Hyaluronan and proteoglycan link protein 1	HAPLN1	A0A8I3PAS0	Described above (nº 93).
**127**	DT1406TB	EV	Over-represented	Fibronectin (Fragment)	FN1	Q28275	Overexpression is correlated with tumour-infiltrating immune cells and therefore poor prognosis in breast cancer [172], gastric cancer [173], head and neck squamous cell carcinoma [174].
**128**	DT1406TB	EV	Over-represented	Thyroglobulin type-1 domain-containing protein	IGFBP6	A0A8I3S4S1	In most studies, its expression is lower in malignant cells than in normal cells. Inhibitor of tumourigenic and metastatic processes. However, it has been described higher expression in adrenocortical tumours, breast cancer and ovarian cancer [175]. Malignant breast tumours with reduced expression of IGFBP6 and ELOVL5 genes can metastasize with a higher probability due to a more efficient invasion of tumours cells [176].
**129**	DT1406TB	EV	Over-represented	Insulin like growth factor binding protein 6	IGFBP6	A0A8P0NSQ8	Described above (nº 128).
**130**	DT1406TB	EV	Over-represented	Protease inhibitor	SLPI	A8QWU1	Highly expressed in gastric cancer tissues, and significantly correlated with survival time, clinical classification and size of the tumour. Involved in metastasis progression [177]. Pro-malignant activity.

Nº	Cell line	Sample type	Condition	Protein name	Protein abbreviation	Accession number	Association with cancer
							Overexpression associated with progression of tumours of different histological origin [178].
**131**	DT1406TB	EV	Over-represented	Protein kinase C and casein kinase substrate in neurons 1	PACSIN1	A0A8P0TAG1	Described as prognostic tool to predict progression of specific breast cancer subtypes [179, 180]. Negatively correlated with malignant degree of gliomas and positively associated with overall survival [181].
**132**	DT1406TB	EV	Over-represented	Olfactomedin like 2B	OLFML2B	A0A8I3Q308	Highly upregulated in gastric cancer [182] and hepatocellular carcinoma. May be a prognostic marker in immunotherapy for diverse tumours [183]. Overexpressed in bladder cancer [184] .
**133**	DT1406TB	EV	Over-represented	Folate_rec domain-containing protein	LOC476816	A0A8I3NGU2	Described above (nº 23).
**134**	DT1406TB	EV	Under-represented	glutaminyl-peptide cyclotransferase	QPCT	A0A8I3PHH6	Diagnostic marker for papillary thyroid carcinoma [185]. High level is correlated with decreased rate of overall survival in adrenocortical carcinoma, mesothelioma, and brain lower grade glioma, whereas in other types of cancer the overexpression indicates a higher survival rate, including cholangiocarcinoma and colon adenocarcinoma [186]. Expressed in local advanced breast tumours and positively correlated with poor-disease free survival [187].
**135**	DT1406TB	EV	Under-represented	Mitochondrial proton/calcium exchanger protein	LETM1	A0A8I3RZY0	Overexpressed and associated with poor prognosis in colorectal [188], liver [189], breast, oesophagus, lung, ovary, stomach and uterine cancer. In thyroid, prostate, ovarian and gastric cancer, overexpression is correlated with increased cell survival [190].
**136**	DT1406TB	EV	Under-represented	Tenascin N	TNN	A0A8P0SUA9	Described above (nº 84).
**137**	DT1406TB	EV	Under-represented	Collagen alpha-1(I) chain	COL1A1	Q9XSJ7	Described above (nº 117).
**138**	DT1406TB	EV	Under-represented	Exportin 6	XPO6	A0A8P0SLL9	Described above (nº 98).
**139**	DT1406TB	EV	Under-represented	Ribosomal protein L37a	3 SV	A0A8P0SQL8	Significantly downregulated in nasopharyngeal carcinoma tissues [191].
**140**	DT1406TB	EV	Under-represented	Histone H3	H3-3A	E2R6K5	Overexpressed in lung cancer, associated with cancer progression [192].
**141**	DT1406TB	EV	Under-represented	WD repeat domain 3	WDR3	A0A8P0SET5	Overexpression is correlated with poor survival in pancreatic cancer [193], prostate cancer [194]. WDR3 may modulate genome stability in thyroid cancer [195].

Additional file 8. Top 20 differentially over- and under-represented proteins identified in complex carcinoma EVs vs healthy control

Accession number	Protein name	Protein abbreviation	1305 Log2FC	DT1406TB Log2FC
**Common over-represented proteins**				
**A0A8I3P5P0**	Extracellular matrix protein 1	ECM1	10.604	13.144
**A0A8P0TAG1**	Protein kinase C and casein kinase substrate in neurons 1	PACSIN1	8.357	9.461
**A0A8I3MVW9**	Actinin alpha 1	ACTN1	8.729	8.593
**A0A8I3PFD7**	Vascular endothelial growth factor C	VEGFC	8.595	8.504
**A8QWU1**	Protease inhibitor	SLPI	7.094	9.622
**A0A8I3P2C5**	Transforming growth factor-beta-induced protein ig-h3	TGFBI	7.130	9.242
**A0A8I3PI03**	Tetraspanin	TSPAN8	9.109	6.952
**A0A8I3RZ77**	Lipoprotein lipase	LIPG	7.959	8.062
**A0A8P0P533**	Phospholipid scramblase	PLSCR1	9.196	6.351
**A0A8I3N0D6**	Kinesin light chain	KLC3	8.815	6.479
**A0A8I3N4I3**	Trophoblast glycoprotein	TPBG	8.303	6.970
**A0A8I3NSN5**	Fibulin-1	FBLN1	6.716	8.238
**A0A8P0S8N5**	AE binding protein 1	AEBP1	5.782	9.163
**A0A8I3NQQ5**	Complement C2	C2	6.548	8.343
**A0A8I3P7V9**	EGF like repeats and discoidin domains 3	EDIL3	6.214	8.627
**A0A8P0TN04**	PDZ domain-containing protein	AHNAK	7.860	6.865
**A0A8I3MK79**	Vasorin	VASN	6.968	7.693
**A0A8I3Q3F0**	non-specific serine/threonine protein kinase	SLK	6.492	8.125
**A0A8I3P614**	Stanniocalcin 1	STC1	7.889	6.637
**A0A8P0N5R9**	Glycoprotein nmb	GPNMB	6.140	8.249
**Common under-represented proteins**				
**Accession number**	**Protein name**	**Protein abbreviation**	**1305 Log2FC**	**DT1406TB Log2FC**
**A0A8I3PX13**	Histone H3	H3C4	-5.206	-7.153
**A0A8P0SET5**	WD repeat domain 3	WDR3	-4.857	-6.228
**E2R6K5**	Histone H3	H3-3A	-4.080	-6.592
**A0A8I3S4C9**	Interleukin enhancer binding factor 3	ILF3	-5.114	-4.823
**A0A8I3PMM3**	Cellular tumor antigen p53	TP53	-3.954	-5.924
**A0A8P0P879**	CSD domain-containing protein	LOC479011	-4.681	-5.156
**A0A8I3P7A1**	Nucleophosmin/nucleoplasmin 3	NPM3	-5.305	-4.130
**A0A8I3P9E1**	Tubulin alpha chain	TUBA1C	-3.287	-5.946
**A0A8I3P801**	Mitogen-activated protein kinase	MAPK7	-4.579	-4.622
**A0A8I3NP87**	40S ribosomal protein S15	RPS15	-4.872	-4.188
**A0A8I3Q7A7**	RTF1 homolog, Paf1/RNA polymerase II complex component	RTF1	-5.619	-3.401
**A0A8I3Q153**	40S ribosomal protein S13	RPS13	-3.164	-5.723
**A0A8I3RQK5**	Protein phosphatase 2 scaffold subunit Abeta	PPP2R1B	-3.430	-5.398
**A0A8I3Q3G4**	Jumonji domain containing 7	JMJD7	-5.165	-3.635
**A0A8I3MXB4**	Ribosome biogenesis protein BRX1 homolog	BRIX1	-4.577	-4.196
**A0A8P0SB07**	RRM domain-containing protein	LOC475399	-3.845	-4.729
**A0A8I3QL49**	Tubulin alpha chain	LOC610636	-4.273	-4.239
**A0A8I3N3V4**	Cyclin dependent kinase 1	CDK1	-4.702	-3.694
**A0A8I3MVT8**	BSD domain containing 1	BSDC1	-4.397	-3.932
**A0A8I3RVI6**	Exportin 5	XPO5	-4.058	-4.264

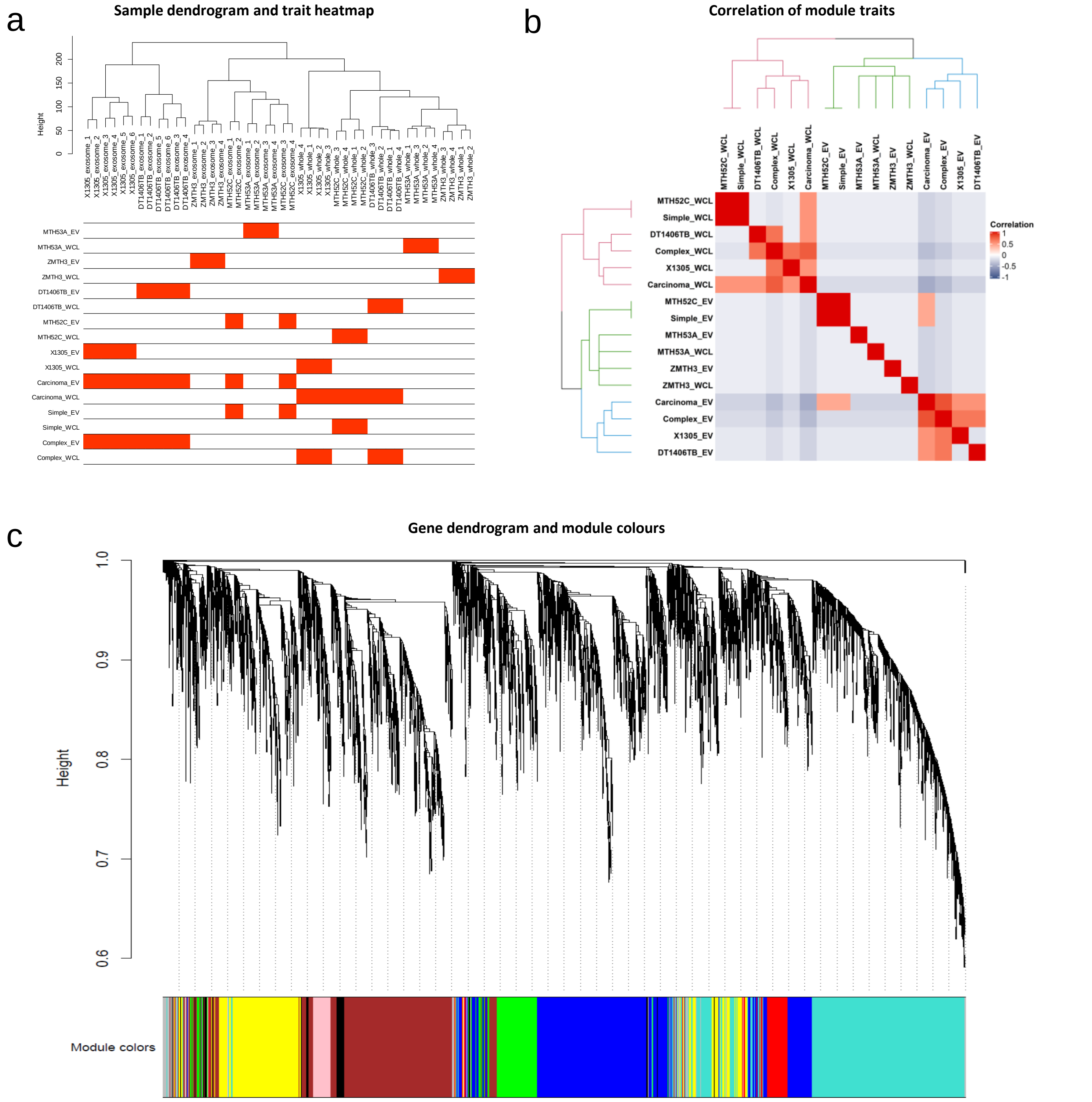

Additional file 9. WGCNA analysis. Identification of modules associated with traits of CMT. **a)** Sample clustering through dendrogram and trait heatmap. Samples were clustered and carcinoma samples grouped in simple carcinoma and complex carcinoma, and traits were subdivided to differentiate WCL and EV-derived protein. **b)** Correlation of module traits. Red represented a positive correlation, while blue represented a negative correlation. **c)** Trait dendrogram and module colours. Clustered proteins were selected into module colours according to the co-abundance of proteins in each sample.

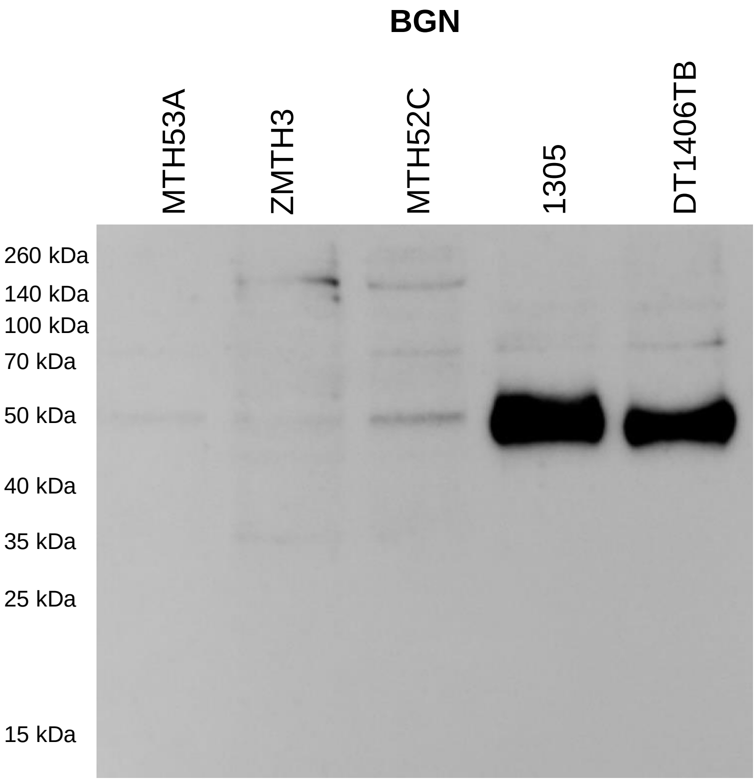

Additional file 10. Western blot of EV proteins for BGN. Samples were de-glycosylated with chondroitinase ABC for 16 hours at 37°C prior to western blotting. EV proteins were then separated by SDS-PAGE and incubated with an anti-BGN antibody as described in Materials and Methods. Images were acquired after 5 minutes of exposure using a Fusion imaging system. The image shows the strong over-representation of BGN in the two complex carcinoma cell lines 1305 and DT1406TB.

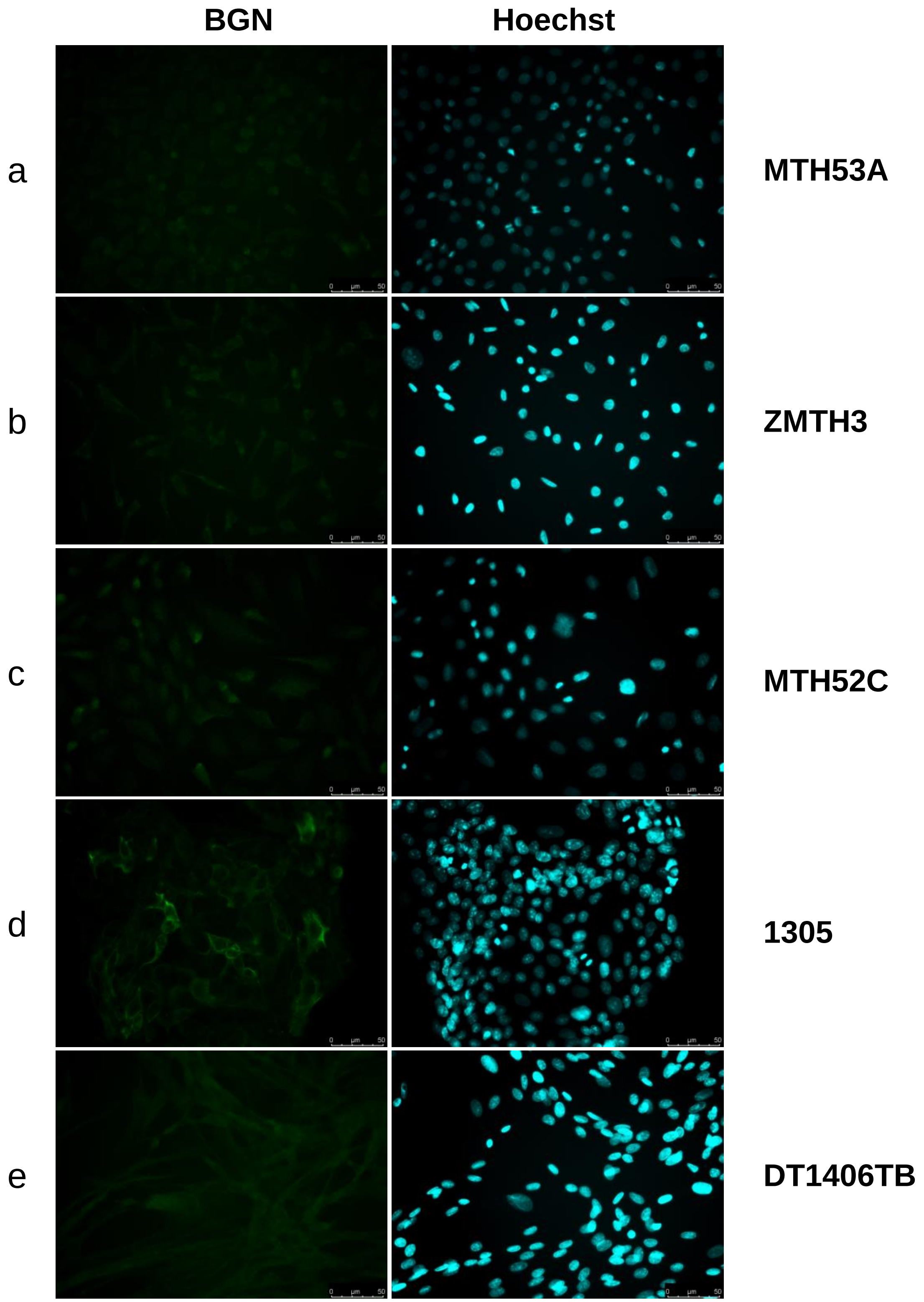

Additional file 11. Immunofluorescence of our cell lines for BGN protein **a)** MTH53A, **b)** ZMTH3, **c)** MTH52C, **d)** 1305, **e)** DT1406TB. Images were acquired with a 40x objective under identical exposure settings.

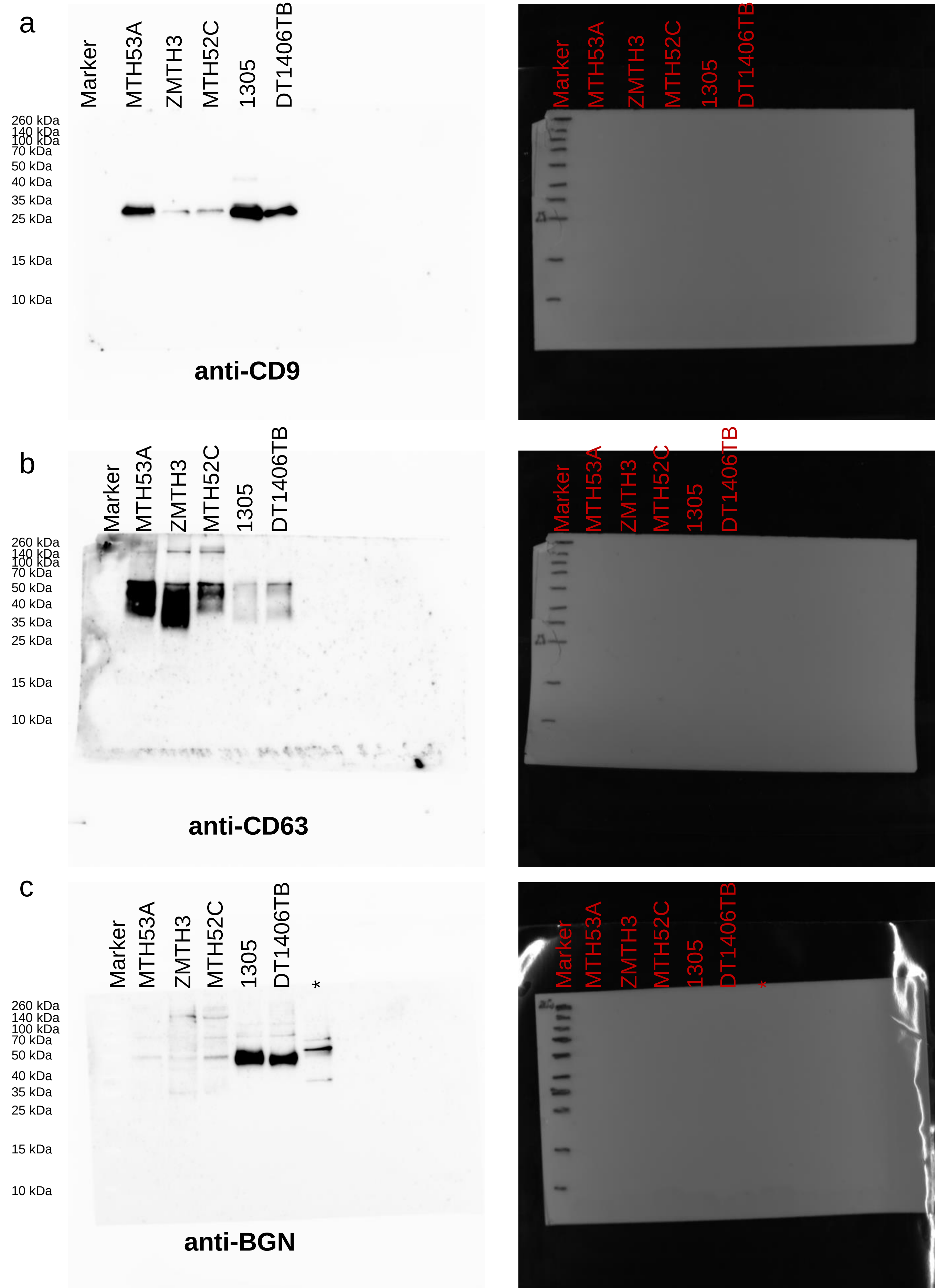

* whole cell lysate of DT1406TB not used in this study

Additional file 12. Original unprocessed images (luminescence left; brightfield on the right) of western blots shown in Additional file 5 (**a)** CD9, **b)** CD63) and Additional file 10 (**c)** BGN). Images were acquired after 5 minutes of exposure using a Fusion imaging system (default settings).

References for Additional file 3 and 7

1. Li Y, Guo L, Ying S, Feng GH, Zhang Y: **Transcriptional repression of p21 by EIF1AX promotes the proliferation of breast cancer cells**. *Cell Proliferation* 2020, **53**(10):e12903.
2. Bandargal S, Chen T, Pusztaszeri MP, Forest V-I, da Silva SD, Payne RJ: **Prognostic Indicators of EIF1AX-Mutated Thyroid Tumor Malignancy and Cancer Aggressiveness**. *Cancers* 2022, **14**(24):6097.
3. Simões-Pereira J, Moura M, Marques I, Rito M, Cabrera R, Leite V, Cavaco B: **The role of EIF1AX in thyroid cancer tumourigenesis and progression**. *Journal of Endocrinological Investigation* 2019, **42**:313-318.
4. Zhao Z, Liu M, Xu Z, Cai Y, Peng B, Liang Q, Yan Y, Liu W, Kang F, He Q: **Identification of ACSF gene family as therapeutic targets and immune-associated biomarkers in hepatocellular carcinoma**. *Aging (Albany NY)* 2022, **14**(19):7926.
5. Hong J, Shiba-Ishii A, Kim Y, Noguchi M, Sakamoto N: **Ovarian carcinoma immunoreactive antigen domain 2 controls mitochondrial apoptosis in lung adenocarcinoma**. *Cancer Science* 2021, **112**(12):5114-5126.
6. Martuszewska D, Ljungberg B, Johansson M, Landberg G, Oslakovic C, Dahlbäck B, Hafizi S: **Tensin3 is a negative regulator of cell migration and all four Tensin family members are downregulated in human kidney cancer**. *PloS one* 2009, **4**(2):e4350.
7. Kang JA, Kim YJ, Jeon YJ: **The diverse repertoire of ISG15: More intricate than initially thought**. *Experimental & Molecular Medicine* 2022, **54**(11):1779-1792.
8. Tokuchi K, Kitamura S, Maeda T, Watanabe M, Hatakeyama S, Kano S, Tanaka S, Ujiie H, Yanagi T: **Loss of FAM83H promotes cell migration and invasion in cutaneous squamous cell carcinoma via impaired keratin distribution**. *Journal of Dermatological Science* 2021, **104**(2):112-121.
9. Kim KM, Hussein UK, Park S-H, Kang M, Moon YJ, Zhang Z, Song Y, Park HS, Bae JS, Park B-H: **FAM83H is involved in stabilization of β-catenin and progression of osteosarcomas**. *Journal of Experimental & Clinical Cancer Research* 2019, **38**(1):1-15.
10. Kim KM, Park S-H, Bae JS, Noh SJ, Tao G-Z, Kim JR, Kwon KS, Park HS, Park B-H, Lee H: **FAM83H is involved in the progression of hepatocellular carcinoma and is regulated by MYC**. *Scientific reports* 2017, **7**(1):3274.
11. Gao Y-F, Zhu T, Mao C-X, Liu Z-X, Wang Z-B, Mao X-Y, Li L, Yin J-Y, Zhou H-H, Liu Z-Q: **PPIC, EMP3 and CHI3L1 are novel prognostic markers for high grade glioma**. *International journal of molecular sciences* 2016, **17**(11):1808.
12. Yu S, Zhao H, Meng H, Shi S, Cao S, Bian T, Ruan C: **CSRP1 Promotes Colon Adenocarcinoma Growth and Serves as an Independent Risk Biomarker for Worse Prognosis**. *Genetics Research* 2023, **2023**.
13. Li D, Yu T, Hu J, Wu J, Feng S, Xu Q, Zhu H, Zhang X, Zhang Y, Zhou B: **Downregulation of CYP39A1 serves as a novel biomarker in hepatocellular carcinoma with worse clinical outcome**. *Oxidative Medicine and Cellular Longevity* 2021, **2021**.
14. Li F, Zhu D, Yang Y, Wu K, Zhao S: **Overexpression of calcyphosine is associated with poor prognosis in esophageal squamous cell carcinoma**. *Oncology Letters* 2017, **14**(5):6231-6237.
15. Sadr-Nabavi A, Bouromand-Noughabi S, Tayebi-Meybodi N, Dadkhah K, Amini N, Meindl A, Abbaszadegan MR: **Non-collagenous extracellular matrix protein dermatopontin may play a role as another component of transforming growth factor-β signaling pathway in colon carcinogenesis**. *Iranian Journal of Basic Medical Sciences* 2021, **24**(4):444.
16. Yamatoji M, Kasamatsu A, Kouzu Y, Koike H, Sakamoto Y, Ogawara K, Shiiba M, Tanzawa H, Uzawa K: **Dermatopontin: a potential predictor for metastasis of human oral cancer**. *International journal of cancer* 2012, **130**(12):2903-2911.

17. Liu S, Qiu J, He G, Geng C, He W, Liu C, Cai D, Pan H, Tian Q: **Dermatopontin inhibits WNT signaling pathway via CXXC finger protein 4 in hepatocellular carcinoma**. *Journal of Cancer* 2020, **11**(21):6288.
18. Lu Y, Yu J, Dong Q, Du Y, Liang Z: **DOCK4 as a Potential Biomarker Associated with Immune Infiltration in Stomach Adenocarcinoma: A Database Analysis**. *International Journal of General Medicine* 2022:6127-6143.
19. Westbrook JA, Wood SL, Cairns DA, McMahon K, Gahlaut R, Thygesen H, Shires M, Roberts S, Marshall H, Oliva MR: **Identification and validation of DOCK4 as a potential biomarker for risk of bone metastasis development in patients with early breast cancer**. *The Journal of pathology* 2019, **247**(3):381-391.
20. Wang Y, Zhao S, Qin Q, Gao X, Zhang X, Zhang M, Jiang Y, Ji X, Zhu H, Zhao X: **CKMT1A is a novel potential prognostic biomarker in patients with endometrial cancer**. *Plos one* 2022, **17**(1):e0262000.
21. Chen Y, Ning J, Cao W, Wang S, Du T, Jiang J, Feng X, Zhang B: **Research progress of TXNIP as a tumor suppressor gene participating in the metabolic reprogramming and oxidative stress of cancer cells in various cancers**. *Frontiers in oncology* 2020, **10**:568574.
22. Wang Z, Hulsurkar M, Zhuo L, Xu J, Yang H, Naderinezhad S, Wang L, Zhang G, Ai N, Li L: **CKB inhibits epithelial-mesenchymal transition and prostate cancer progression by sequestering and inhibiting AKT activation**. *Neoplasia* 2021, **23**(11):1147-1165.
23. Mooney SM, Rajagopalan K, Williams BH, Zeng Y, Christudass CS, Li Y, Yin B, Kulkarni P, Getzenberg RH: **Creatine kinase brain overexpression protects colorectal cells from various metabolic and non-metabolic stresses**. *Journal of cellular biochemistry* 2011, **112**(4):1066-1075.
24. Niu M, Shan M, Liu Y, Song Y, Han J-g, Sun S, Liang X-s, Zhang G-q: **DCTPP1, an oncogene regulated by miR-378a-3p, promotes proliferation of breast cancer via DNA repair signaling pathway**. *Frontiers in oncology* 2021, **11**:641931.
25. Hoang TM, Bui TTH, Nguyen TT: **Comprehensive Analysis of the Expression and Prognosis for DCTPP1 gene in Breast Cancer**. *Asian Pacific Journal of Cancer Biology* 2021, **6**(3):195-200.
26. Chu Y-H, Su C-W, Hsieh Y-S, Chen P-N, Lin C-W, Yang S-F: **Carbonic Anhydrase III Promotes Cell Migration and Epithelial–Mesenchymal Transition in Oral Squamous Cell Carcinoma**. *Cells* 2020, **9**(3):704.
27. Zhao X, Jiang M, Wang Z: **TPM4 promotes cell migration by modulating F-actin formation in lung cancer**. *OncoTargets and therapy* 2019:4055-4063.
28. Shi F, Wu H, Qu K, Sun Q, Li F, Shi C, Li Y, Xiong X, Qin Q, Yu T: **Identification of serum proteins AHSG, FGA and APOA-I as diagnostic biomarkers for gastric cancer**. *Clinical proteomics* 2018, **15**:1-13.
29. Ochieng J, Nangami G, Sakwe A, Moye C, Alvarez J, Whalen D, Thomas P, Lammers P: **Impact of Fetuin-A (AHSG) on tumor progression and type 2 diabetes**. *International Journal of Molecular Sciences* 2018, **19**(8):2211.
30. Zhu Y, Qi X, Yu C, Yu S, Zhang C, Zhang Y, Liu X, Xu Y, Yang C, Jiang W: **Identification of prothymosin alpha (PTMA) as a biomarker for esophageal squamous cell carcinoma (ESCC) by label-free quantitative proteomics and Quantitative Dot Blot (QDB)**. *Clinical Proteomics* 2019, **16**:1-20.
31. Tsai Y-S, Jou Y-C, Lee G-F, Chen Y-C, Shiau A-L, Tsai H-T, Wu C-L, Tzai T-S: **Aberrant prothymosin-alpha expression in human bladder cancer**. *Urology* 2009, **73**(1):188-192.
32. Miyoshi N, Ishii H, Mimori K, Nishida N, Tokuoka M, Akita H, Sekimoto M, Doki Y, Mori M: **Abnormal expression of PFDN4 in colorectal cancer: a novel marker for prognosis**. *Annals of surgical oncology* 2010, **17**:3030-3036.
33. Yuan Y, Nymoen DA, Dong HP, Bjørang O, Shih I-M, Low PS, Trope CG, Davidson B: **Expression of the folate receptor genes FOLR1 and FOLR3 differentiates ovarian carcinoma from breast carcinoma and malignant mesothelioma in serous effusions**. *Human pathology* 2009, **40**(10):1453-1460.
34. Yuan K, Chen Z, Li W, Gao CE, Li G, Guo G, Yang Y, Ai Y, Wu L, Zhang M: **MDK protein overexpression correlates with the malignant status and prognosis of non-small cell lung cancer**. *Archives of medical research* 2015, **46**(8):635-641.

35. Shin DH, Jo JY, Kim SH, Choi M, Han C, Choi BK, Kim SS: **Midkine is a potential therapeutic target of tumorigenesis, angiogenesis, and metastasis in non-small cell lung cancer**. *Cancers* 2020, **12**(9):2402.
36. Filippou PS, Karagiannis GS, Constantinidou A: **Midkine (MDK) growth factor: a key player in cancer progression and a promising therapeutic target**. *Oncogene* 2020, **39**(10):2040-2054.
37. Pathak GP, Shah R, Kennedy BE, Murphy JP, Clements D, Konda P, Giacomantonio M, Xu Z, Schlaepfer IR, Gujar S: **RTN4 knockdown dysregulates the AKT pathway, destabilizes the cytoskeleton, and enhances paclitaxel-induced cytotoxicity in cancers**. *Molecular Therapy* 2018, **26**(8):2019-2033.
38. Direito I, Monteiro L, Melo T, Figueira D, Lobo J, Enes V, Moura G, Henrique R, Santos MA, Jerónimo C: **Protein aggregation patterns inform about breast cancer response to antiestrogens and reveal the RNA ligase RTCB as mediator of acquired tamoxifen resistance**. *Cancers* 2021, **13**(13):3195.
39. Badura M, Braunstein S, Zavadil J, Schneider RJ: **DNA damage and eIF4G1 in breast cancer cells reprogram translation for survival and DNA repair mRNAs**. *Proceedings of the National Academy of Sciences* 2012, **109**(46):18767-18772.
40. Del Valle L, Dai L, Lin HY, Lin Z, Chen J, Post SR, Qin Z: **Role of EIF4G1 network in non-small cell lung cancers (NSCLC) cell survival and disease progression**. *Journal of Cellular and Molecular Medicine* 2021, **25**(6):2795-2805.
41. Yuan Q, Fan Y, Liu Z, Wang X, Jia M, Dong Y, Geng Z, Zheng J, Lu X: **Pleckstrin homology and RhoGEF domain containing G4 (PLEKHG4) leads to the activation of RhoGTPases promoting the malignant phenotypes of thyroid cancer**. *Apoptosis* 2023:1-17.
42. Wang M, Hu Y, Stearns ME: **RPS2: a novel therapeutic target in prostate cancer**. *Journal of Experimental & Clinical Cancer Research* 2009, **28**(1):1-12.
43. Peppino G, Ruiu R, Arigoni M, Riccardo F, Iacoviello A, Barutello G, Quaglino E: **Teneurins: Role in Cancer and Potential Role as Diagnostic Biomarkers and Targets for Therapy**. *International journal of molecular sciences* 2021, **22**(5):2321.
44. Guerrero-Martínez JA, Reyes JC: **High expression of SMARCA4 or SMARCA2 is frequently associated with an opposite prognosis in cancer**. *Scientific reports* 2018, **8**(1):2043.
45. Deng M, Xiong C, He Z-K, Bin Q, Song J-Z, Li W, Qin J: **MCTS1 as a novel prognostic biomarker and its correlation with immune infiltrates in breast cancer**. *Frontiers in Genetics* 2022, **13**:825901.
46. Yu SE: **The role of the MCTS1 and DENR proteins in regulating the mechanisms associated with malignant cell transformation**. *Acta Naturae (англоязычная версия)* 2021, **13**(2):98-105.
47. Huang Z, Su Q, Li W, Ren H, Huang H, Wang A: **MCTS1 promotes invasion and metastasis of oral cancer by modifying the EMT process**. *Annals of Translational Medicine* 2021, **9**(12).
48. Chang I-W, Liu K-W, Ragunanan M, He H-L, Shiue Y-L, Yu S-C: **SERPINB5 expression: association with CCRT response and prognostic value in rectal cancer**. *International Journal of Medical Sciences* 2018, **15**(4):376.
49. Heo K, Lee S: **TSPAN8 as a novel emerging therapeutic target in cancer for monoclonal antibody therapy**. *Biomolecules* 2020, **10**(3):388.
50. Voglstaetter M, Thomsen AR, Nouvel J, Koch A, Jank P, Navarro EG, Gainey-Schleicher T, Khanduri R, Groß A, Rossner F: **Tspan8 is expressed in breast cancer and regulates E-cadherin/catenin signalling and metastasis accompanied by increased circulating extracellular vesicles**. *The Journal of pathology* 2019, **248**(4):421-437.
51. Li Z, Tan Y, Li X, Quan J, Bode AM, Cao Y, Luo X: **DHRS2 inhibits cell growth and metastasis in ovarian cancer by downregulation of CHKα to disrupt choline metabolism**. *Cell Death & Disease* 2022, **13**(10):845.
52. Zhao T, Li Y, Shen K, Wang Q, Zhang J: **Knockdown of OLR1 weakens glycolytic metabolism to repress colon cancer cell proliferation and chemoresistance by downregulating SULT2B1 via c-MYC**. *Cell Death & Disease* 2021, **13**(1):4.

53. Yin L, Chen G: **Verteporfin promotes the apoptosis and inhibits the proliferation, migration, and invasion of cervical cancer cells by downregulating SULT2B1 expression**. *Medical Science Monitor: International Medical Journal of Experimental and Clinical Research* 2020, **26**:e926780-926781.
54. Mohtar MA, Syafruddin SE, Nasir SN, Low TY: **Revisiting the roles of pro-metastatic EpCAM in cancer**. *Biomolecules* 2020, **10**(2):255.
55. Cui W-j, Liu Y, Zhou X-l, Wang F-z, Zhang X-d, Ye L-h: **Myosin light chain kinase is responsible for high proliferative ability of breast cancer cells via anti-apoptosis involving p38 pathway**. *Acta Pharmacologica Sinica* 2010, **31**(6):725-732.
56. Chen S, Huang H, Yao J, Pan L, Ma H: **Heat shock protein B6 potently increases non-small cell lung cancer growth**. *Molecular Medicine Reports* 2014, **10**(2):677-682.
57. Wei R-R, Zhang M-Y, Rao H-L, Pu H-Y, Zhang H-Z, Wang H-Y: **Identification of ADH4 as a novel and potential prognostic marker in hepatocellular carcinoma**. *Medical Oncology* 2012, **29**:2737-2743.
58. Cai D, Cao J, Li Z, Zheng X, Yao Y, Li W, Yuan Z: **Up-regulation of bone marrow stromal protein 2 (BST2) in breast cancer with bone metastasis**. *BMC cancer* 2009, **9**(1):1-10.
59. Mukai S, Oue N, Oshima T, Mukai R, Tatsumoto Y, Sakamoto N, Sentani K, Tanabe K, Egi H, Hinoi T: **Overexpression of transmembrane protein BST2 is associated with poor survival of patients with esophageal, gastric, or colorectal cancer**. *Annals of surgical oncology* 2017, **24**:594-602.
60. Duan Y, Liu T, Li S, Huang M, Li X, Zhao H, Li J: **CHAF1B promotes proliferation and reduces apoptosis in 95-D lung cancer cells and predicts a poor prognosis in non-small cell lung cancer**. *Oncology reports* 2019, **41**(4):2518-2528.
61. Zhang J, Gan Y, Li H, Yin J, He X, Lin L, Xu S, Fang Z, Kim B-w, Gao L: **Inhibition of the CDK2 and Cyclin A complex leads to autophagic degradation of CDK2 in cancer cells**. *Nature communications* 2022, **13**(1):2835.
62. Zhang J, Yang G, Li Q, Xie F: **Increased fibrillarin expression is associated with tumor progression and an unfavorable prognosis in hepatocellular carcinoma**. *Oncology Letters* 2021, **21**(2):1-1.
63. Nguyen Van Long F, Lardy-Cleaud A, Carène D, Rossoni C, Catez F, Rollet P, Pion N, Monchiet D, Dolbeau A, Martin M: **Low level of Fibrillarin, a ribosome biogenesis factor, is a new independent marker of poor outcome in breast cancer**. *BMC cancer* 2022, **22**(1):526.
64. Ladelfa MF, Peche LY, Toledo MF, Laiseca JE, Schneider C, Monte M: **Tumor-specific MAGE proteins as regulators of p53 function**. *Cancer letters* 2012, **325**(1):11-17.
65. Pollok S, Bauerschmidt C, Sänger J, Nasheuer HP, Grosse F: **Human Cdc45 is a proliferation-associated antigen**. *The FEBS journal* 2007, **274**(14):3669-3684.
66. He Z, Wang X, Yang Z, Jiang Y, Li L, Wang X, Song Z, Wang X, Wan J, Jiang S: **Expression and prognosis of CDC45 in cervical cancer based on the GEO database**. *PeerJ* 2021, **9**:e12114.
67. Zhang X, Han S, Zhou H, Cai L, Li J, Liu N, Liu Y, Wang L, Fan C, Li A: **TIMM50 promotes tumor progression via ERK signaling and predicts poor prognosis of non-small cell lung cancer patients**. *Molecular Carcinogenesis* 2019, **58**(5):767-776.
68. Gao S-P, Sun H-F, Jiang H-L, Li L-D, Hu X, Xu X-E, Jin W: **Loss of TIM50 suppresses proliferation and induces apoptosis in breast cancer**. *Tumor Biology* 2016, **37**:1279-1287.
69. Mboge MY, Mahon BP, McKenna R, Frost SC: **Carbonic anhydrases: role in pH control and cancer**. *Metabolites* 2018, **8**(1):19.
70. Wang T, Tang T, Jiang Y, He T, Qi L, Chang H, Qiao Y, Sun M, Shan C, Zhu X: **PRIM2 Promotes Cell Cycle and Tumor Progression in p53-Mutant Lung Cancer**. *Cancers* 2022, **14**(14):3370.
71. Johnston SJ, Ridge SA, Cassidy J, McLeod HL: **Regulation of dihydropyrimidine dehydrogenase in colorectal cancer**. *Clinical Cancer Research* 1999, **5**(9):2566-2570.
72. Meng L, Yang X, Xie X, Wang M: **Mitochondrial NDUFA4L2 protein promotes the vitality of lung cancer cells by repressing oxidative stress**. *Thoracic Cancer* 2019, **10**(4):676-685.

73. Chen Z, Wei X, Wang X, Zheng X, Chang B, Shen L, Zhu H, Yang M, Li S, Zheng X: **NDUFA4L2 promotes glioblastoma progression, is associated with poor survival, and can be effectively targeted by apatinib**. *Cell Death & Disease* 2021, **12**(4):377.
74. Lv Y, Nie SL, Zhou JM, Liu F, Hu YB, Jiang JR, Li N, Liu JS: **Overexpression of NDUFA4L2 is associated with poor prognosis in patients with colorectal cancer**. *ANZ journal of surgery* 2017, **87**(12):E251-E255.
75. Liu X, Wang J, Chen M, Liu S, Yu X, Wen F: **Combining data from TCGA and GEO databases and reverse transcription quantitative PCR validation to identify gene prognostic markers in lung cancer**. *OncoTargets and therapy* 2019, **12**:709.
76. Handa T, Katayama A, Yokobori T, Yamane A, Horiguchi J, Kawabata-Iwakawa R, Rokudai S, Bao P, Gombodorj N, Altan B: **Caspase14 expression is associated with triple negative phenotypes and cancer stem cell marker expression in breast cancer patients**. *Journal of Surgical Oncology* 2017, **116**(6):706-715.
77. Al-Juboori SI, Vadakekolathu J, Idri S, Wagner S, Zafeiris D, Pearson JR, Almshayakhchi R, Caraglia M, Desiderio V, Miles AK: **PYK2 promotes HER2-positive breast cancer invasion**. *Journal of Experimental & Clinical Cancer Research* 2019, **38**(1):1-14.
78. Dai D, Chen B, Feng Y, Wang W, Jiang Y, Huang H, Liu J: **Prognostic value of prostaglandin I2 synthase and its correlation with tumor-infiltrating immune cells in lung cancer, ovarian cancer, and gastric cancer**. *Aging (Albany NY)* 2020, **12**(10):9658.
79. Wu X, Lu W, Xu C, Jiang C, Zhang W, Zhang D, Cui S, Zhuo Z, Cui Y, Mei H: **PTGIS May Be a Predictive Marker for Ovarian Cancer by Regulating Fatty Acid Metabolism**. *Computational and Mathematical Methods in Medicine* 2023, **2023**.
80. El Ansari R, Craze ML, Miligy I, Diez-Rodriguez M, Nolan CC, Ellis IO, Rakha EA, Green AR: **The amino acid transporter SLC7A5 confers a poor prognosis in the highly proliferative breast cancer subtypes and is a key therapeutic target in luminal B tumours**. *Breast Cancer Research* 2018, **20**:1-17.
81. Chen X, Wang L, Yu H, Shen Q, Hou Y, Xia Y-X, Li L, Chang L, Li W-H: **Irradiated lung cancer cell-derived exosomes modulate macrophage polarization by inhibiting MID1 via miR-4655-5p**. *Molecular Immunology* 2023, **155**:58-68.
82. Zhang W, Wu C, Zhou K, Cao Y, Zhou W, Zhang X, Deng D: **Clinical and immunological characteristics of TGM3 in pan-cancer: A potential prognostic biomarker**. *Frontiers in Genetics* 2023, **13**:993438.
83. Wu X, Cao W, Wang X, Zhang J, Lv Z, Qin X, Wu Y, Chen W: **TGM3, a candidate tumor suppressor gene, contributes to human head and neck cancer**. *Molecular cancer* 2013, **12**(1):1-13.
84. Wu Y, Xu Y: **Bioinformatics for the prognostic value and function of cubilin (CUBN) in colorectal cancer**. *Medical science monitor: international medical journal of experimental and clinical research* 2020, **26**:e922447-922441.
85. Achari C, Winslow S, Larsson C: **Down regulation of CLDND1 induces apoptosis in breast cancer cells**. *PLoS One* 2015, **10**(6):e0130300.
86. Yoshimaru T, Nakamura Y, Katagiri T: **Functional genomics for breast cancer drug target discovery**. *Journal of Human Genetics* 2021, **66**(9):927-935.
87. Song X, Yang W, Wu C, Han Y, Lu Y: **USP9X promotes the proliferation, invasion and metastasis of liver cancer cells through regulating the JAK2/STAT3 signaling**. *Oncology Letters* 2020, **20**(3):2897-2905.
88. Chen W, Song J, Liu S, Tang B, Shen L, Zhu J, Fang S, Wu F, Zheng L, Qiu R: **USP9X promotes apoptosis in cholangiocarcinoma by modulation expression of KIF1Bβ via deubiquitinating EGLN3**. *Journal of Biomedical Science* 2021, **28**(1):44.
89. Hu X, Han Y, Liu J, Wang H, Tian Z, Zhang X, Zhang Y, Wang X: **CTP synthase 2 predicts inferior survival and mediates DNA damage response via interacting with BRCA1 in chronic lymphocytic leukemia**. *Experimental Hematology & Oncology* 2023, **12**(1):6.
90. Gao F, Hu X, Liu W, Wu H, Mu Y, Zhao Y: **Calcium-activated nucleotides 1 (CANT1)-driven nuclear factor-k-gene binding (NF-κB) signaling pathway facilitates the lung cancer progression**. *Bioengineered* 2022, **13**(2):3183-3193.
91. Yao Q, Yu Y, Wang Z, Zhang M, Ma J, Wu Y, Zheng Q, Li J: **CANT1 serves as a potential prognostic factor for lung adenocarcinoma and promotes cell proliferation and invasion in vitro**. *BMC cancer* 2022, **22**(1):1-17.

92. Gerhardt J, Steinbrech C, Büchi O, Behnke S, Bohnert A, Fritzsche F, Liewen H, Stenner F, Wild P, Hermanns T: **The androgen-regulated Calcium-Activated Nucleotidase 1 (CANT1) is commonly overexpressed in prostate cancer and is tumor-biologically relevant in vitro**. *The American journal of pathology* 2011, **178**(4):1847-1860.
93. Zhang K, Xu P, Sowers JL, Machuca DF, Mirfattah B, Herring J, Tang H, Chen Y, Tian B, Brasier AR: **Proteome analysis of hypoxic glioblastoma cells reveals sequential metabolic adaptation of one-carbon metabolic pathways**. *Molecular & Cellular Proteomics* 2017, **16**(11):1906-1921.
94. Sysel AM, Valli VE, Bauer JA: **Quantification of the cobalamin (vitamin B12) TCN2 transport protein, cobalamin CD320 cell surface receptor and Ki-67 in naturally occurring canine and feline tumors and comparison to expression in adjacent normal tissues**. *Cancer Research* 2015, **75**(15_Supplement):553-553.
95. Xu W, Wang M, Bai Y, Chen Y, Ma X, Yang Z, Zhao L, Li Y: **The role of microfibrillar-associated protein 2 in cancer**. *Frontiers in Oncology* 2022, **12**:1002036.
96. Zhu X, Cheng Y, Wu F, Sun H, Zheng W, Jiang W, Shi J, Ma S, Cao H: **MFAP2 promotes the proliferation of cancer cells and is associated with a poor prognosis in hepatocellular carcinoma**. *Technology in Cancer Research & Treatment* 2020, **19**:1533033820977524.
97. Van Duzer A, Taniguchi S, Elhance A, Tsujikawa T, Oshimori N: **ADAP1 promotes invasive squamous cell carcinoma progression and predicts patient survival**. *Life science alliance* 2019, **2**(6).
98. Talebi A, Shahidsales S, Aliakbarian M, Pezeshki Rad M, Kerachian MA: **Oncogenic fusion transcript analysis identified ADAP1-NOC4L, potentially associated with metastatic colorectal cancer**. *Cancer Medicine* 2023, **12**(1):525-540.
99. Knepper MI, Dernedde J: **Zona Pellucida like Domain Protein 1 (ZPLD1) Polymerization Is Regulated by Two Distinguished Hydrophobic Motifs**. *International Journal of Molecular Sciences* 2022, **23**(22):13894.
100. Pan H, Wang X, Huang W, Dai Y, Yang M, Liang H, Wu X, Zhang L, Huang W, Yuan L: **Interferon-induced protein 44 correlated with immune infiltration serves as a potential prognostic indicator in head and neck squamous cell carcinoma**. *Frontiers in Oncology* 2020, **10**:557157.
101. Chan H-S, Chang S-J, Wang T-Y, Ko H-J, Lin Y-C, Lin K-T, Chang K-M, Chuang Y-J: **Serine protease PRSS23 is upregulated by estrogen receptor α and associated with proliferation of breast cancer cells**. *PLoS One* 2012, **7**(1):e30397.
102. Qin S, Wang Z, Huang C, Huang P, Li D: **Serine protease PRSS23 drives gastric cancer by enhancing tumor associated macrophage infiltration via FGF2**. *Frontiers in Immunology* 2022, **13**:955841.
103. Ichihara R, Shiraki Y, Mizutani Y, Iida T, Miyai Y, Esaki N, Kato A, Mii S, Ando R, Hayashi M: **Matrix remodeling-associated protein 8 is a marker of a subset of cancer-associated fibroblasts in pancreatic cancer**. *Pathology International* 2022, **72**(3):161-175.
104. Tan L, Fu D, Liu F, Liu J, Zhang Y, Li X, Gao J, Tao K, Wang G, Wang L: **MXRA8 is an immune-relative prognostic biomarker associated with metastasis and CD8+ T cell infiltration in colorectal cancer**. *Frontiers in Oncology* 2023, **12**:1094612.
105. Yang L, Sun R, Wang Y, Fu Y, Zhang Y, Zheng Z, Ji Z, Zhao D: **Expression of ANGPTL2 and its impact on papillary thyroid cancer**. *Cancer Cell International* 2019, **19**(1):1-10.
106. Osumi H, Horiguchi H, Kadomatsu T, Tashiro K, Morinaga J, Takahashi T, Ikeda K, Ito T, Suzuki M, Endo M: **Tumor cell-derived angiopoietin-like protein 2 establishes a preference for glycolytic metabolism in lung cancer cells**. *Cancer science* 2020, **111**(4):1241-1253.
107. Horiguchi H, Kadomatsu T, Kurahashi R, Hara C, Miyata K, Baba M, Osumi H, Terada K, Araki K, Takai T: **Dual functions of angiopoietin-like protein 2 signaling in tumor progression and anti-tumor immunity**. *Genes & development* 2019, **33**(23-24):1641-1656.
108. Brellier F, Martina E, Degen M, Heuzé-Vourc'h N, Petit A, Kryza T, Courty Y, Terracciano L, Ruiz C, Chiquet-Ehrismann R: **Tenascin-W is a better cancer biomarker than tenascin-C for most human solid tumors**. *BMC clinical pathology* 2012, **12**:1-10.
109. Degen M, Brellier F, Kain R, Ruiz C, Terracciano L, Orend G, Chiquet-Ehrismann R: **Tenascin-W is a novel marker for activated tumor stroma in low-grade human breast cancer and influences cell behavior**. *Cancer research* 2007, **67**(19):9169-9179.

110. Degen M, Brellier F, Schenk S, Driscoll R, Zaman K, Stupp R, Tornillo L, Terracciano L, Chiquet-Ehrismann R, Rüegg C: **Tenascin-W, a new marker of cancer stroma, is elevated in sera of colon and breast cancer patients**. *International journal of cancer* 2008, **122**(11):2454-2461.
111. Doni A, Stravalaci M, Inforzato A, Magrini E, Mantovani A, Garlanda C, Bottazzi B: **The long pentraxin PTX3 as a link between innate immunity, tissue remodeling, and cancer**. *Frontiers in Immunology* 2019, **10**:712.
112. Koc EC, Koc FC, Kartal F, Tirona M, Koc H: **Role of mitochondrial translation in remodeling of energy metabolism in ER/PR (+) breast cancer**. *Frontiers in Oncology* 2022, **12**:897207.
113. Li M, Wang Z, Zhu L, Shui Y, Zhang S, Guo W: **Down-regulation of RBP4 indicates a poor prognosis and correlates with immune cell infiltration in hepatocellular carcinoma**. *Bioscience reports* 2021, **41**(4):BSR20210328.
114. Deng X, Ren J, Bi Z, Fu Z: **Positive Expression of Retinol-Binding Protein 4 Is Related to the Malignant Clinical Features Leading to Poor Prognosis of Glioblastoma**. *Genetics Research* 2022, **2022**.
115. Chen X, Li X, Hu X, Jiang F, Shen Y, Xu R, Wu L, Wei P, Shen X: **LUM expression and its prognostic significance in gastric cancer**. *Frontiers in oncology* 2020, **10**:605.
116. Gilbertson RJ, Clifford SC: **PDGFRB is overexpressed in metastatic medulloblastoma**. *Nature genetics* 2003, **35**(3):197-198.
117. Xu G, Li JY: **Differential expression of PDGFRB and EGFR in microvascular proliferation in glioblastoma**. *Tumor Biology* 2016, **37**:10577-10586.
118. Li Z, Shi J, Zhang N, Zheng X, Jin Y, Wen S, Hu W, Wu Y, Gao W: **FSCN1 acts as a promising therapeutic target in the blockade of tumor cell motility: a review of its function, mechanism, and clinical significance**. *Journal of Cancer* 2022, **13**(8):2528.
119. Wiedmann L, De Angelis Rigotti F, Vaquero-Siguero N, Donato E, Espinet E, Moll I, Alsina-Sanchis E, Bohnenberger H, Fernandez-Florido E, Mülfarth R: **HAPLN1 potentiates peritoneal metastasis in pancreatic cancer**. *Nature Communications* 2023, **14**(1):2353.
120. Kim Y-S, Sun DS, Ahn J, Kim Y, Yoon J-S, Won HS: **Analysis of Genomic Alterations Associated with Recurrence in Early Stage HER2-Positive Breast Cancer**. *Cancers* 2022, **14**(15):3650.
121. Mebarki S, Désert R, Sulpice L, Sicard M, Desille M, Canal F, Schneider HD-P, Bergeat D, Turlin B, Bellaud P: **De novo HAPLN1 expression hallmarks Wnt-induced stem cell and fibrogenic networks leading to aggressive human hepatocellular carcinomas**. *Oncotarget* 2016, **7**(26):39026.
122. Ivanova AV, Goparaju CM, Ivanov SV, Nonaka D, Cruz C, Beck A, Lonardo F, Wali A, Pass HI: **Protumorigenic role of HAPLN1 and its IgV domain in malignant pleural mesothelioma**. *Clinical cancer research* 2009, **15**(8):2602-2611.
123. Zhang T, Li X, He Y, Wang Y, Shen J, Wang S, You Q, Zhai J, Shen L: **Cancer-associated fibroblasts-derived HAPLN1 promotes tumour invasion through extracellular matrix remodeling in gastric cancer**. *Gastric Cancer* 2022:1-14.
124. Chang SC, Yuan SHC, Li CY, Chang HM, Wang HC, Pan YA, Hsueh PC, Wu CC, Yang Y, Liu HP: **Significant association of serum autoantibodies to TYMS, HAPLN1 and IGFBP5 with early stage canine malignant mammary tumours**. *Veterinary and Comparative Oncology* 2021, **19**(1):172-182.
125. Jiang X, Wu M, Xu X, Zhang L, Huang Y, Xu Z, He K, Wang H, Wang H, Teng L: **COL12A1, a novel potential prognostic factor and therapeutic target in gastric cancer**. *Molecular medicine reports* 2019, **20**(4):3103-3112.
126. Yan Y, Liang Q, Liu Y, Zhou S, Xu Z: **COL12A1 as a prognostic biomarker links immunotherapy response in breast cancer**. *Endocrine-Related Cancer* 2023, **30**(5).
127. Miyakawa R, Kobayashi M, Sugimoto K, Endo Y, Kojima M, Kobayashi Y, Furukawa S, Honda T, Watanabe T, Asano S: **SPON1 is an independent prognostic biomarker for ovarian cancer**. *Journal of Ovarian Research* 2023, **16**(1):95.
128. Huo Y, Yang J, Zheng J, Xu D, Yang M, Tao L, Yao H, Fu X, Yang J, Liu D: **Increased SPON1 promotes pancreatic ductal adenocarcinoma progression by enhancing IL-6 trans-signalling**. *Cell Proliferation* 2022, **55**(5):e13237.
129. Schulze M, Violonchi C, Swoboda S, Welz T, Kerkhoff E, Hoja S, Brüggemann S, Simbürger J, Reinders J, Riemenschneider MJ: **RELN signaling modulates glioblastoma growth and substrate-dependent migration**. *Brain Pathology* 2018, **28**(5):695-709.

130. Stein T, Cosimo E, Yu X, Smith PR, Simon R, Cottrell L, Pringle M-A, Bell AK, Lattanzio L, Sauter G: **Loss of reelin expression in breast cancer is epigenetically controlled and associated with poor prognosis**. *The American journal of pathology* 2010, **177**(5):2323-2333.
131. Perrone G, Vincenzi B, Zagami M, Santini D, Panteri R, Flammia G, Verzì A, Lepanto D, Morini S, Russo A: **Reelin expression in human prostate cancer: a marker of tumor aggressiveness based on correlation with grade**. *Modern pathology* 2007, **20**(3):344-351.
132. Yin C, Zhang J, Guan W, Dou L, Liu Y, Shen M, Jia X, Xu L, Wu R, Li Y: **High expression of clec11a predicts favorable prognosis in acute myeloid leukemia**. *Frontiers in Oncology* 2021, **11**:608932.
133. Wang M, Guo J, Zhang L, Kuek V, Xu J, Zou J: **Molecular structure, expression, and functional role of Clec11a in skeletal biology and cancers**. *Journal of Cellular Physiology* 2020, **235**(10):6357-6365.
134. Zhu C, Kim S-J, Mooradian A, Wang F, Li Z, Holohan S, Collins PL, Wang K, Guo Z, Hoog J: **Cancer-associated exportin-6 upregulation inhibits the transcriptionally repressive and anticancer effects of nuclear profilin-1**. *Cell reports* 2021, **34**(7).
135. Wang H, Teng X, Lin Y, Jiang C, Chen X, Zhang Y: **Targeting XPO6 inhibits prostate cancer progression and enhances the suppressive efficacy of docetaxel**. *Discover Oncology* 2023, **14**(1):82.
136. Gorka B, Skubis-Zegadło J, Mikula M, Bardadin K, Paliczka E, Czarnocka B: **NrCAM, a neuronal system cell-adhesion molecule, is induced in papillary thyroid carcinomas**. *British journal of cancer* 2007, **97**(4):531-538.
137. Janik J, Czarnocka B: **NRCAM (neuronal cell adhesion molecule)**. 2010.
138. Bai C, Chen D-g: **NRCAM acts as a prognostic biomarker and promotes the tumor progression in gastric cancer via EMT pathway**. *Tissue and Cell* 2022, **77**:101859.
139. Kim Y, You JH, Ryu Y, Park G, Lee U, Moon HE, Park HR, Song C, Ku J-L, Park S-H: **Loss of ELAVL2 is associated with aggressive mesenchymal transition in glioblastoma**. 2023.
140. Towler BP, Pashler AL, Haime HJ, Przybyl KM, Viegas SC, Matos RG, Morley SJ, Arraiano CM, Newbury SF: **Dis3L2 regulates cell proliferation and tissue growth through a conserved mechanism**. *PLoS genetics* 2020, **16**(12):e1009297.
141. Chen ZG, Saba NF, Teng Y: **The diverse functions of FAT1 in cancer progression: good, bad, or ugly?** *Journal of Experimental & Clinical Cancer Research* 2022, **41**(1):248.
142. Wang L, Lyu S, Wang S, Shen H, Niu F, Liu X, Liu J, Niu Y: **Loss of FAT1 during the progression from DCIS to IDC and predict poor clinical outcome in breast cancer**. *Experimental and molecular pathology* 2016, **100**(1):177-183.
143. Kim H, Son S, Shin I: **Role of the CCN protein family in cancer**. *BMB reports* 2018, **51**(10):486.
144. Kleer CG: **Dual roles of CCN proteins in breast cancer progression**. *Journal of cell communication and signaling* 2016, **10**:217-222.
145. Ma AP, Yeung CL, Tey SK, Mao X, Wong SW, Ng TH, Ko FC, Kwong EM, Tang AH, Ng IO-L: **Suppression of ACADM-mediated fatty acid oxidation promotes hepatocellular carcinoma via aberrant CAV1/SREBP1 signaling**. *Cancer research* 2021, **81**(13):3679-3692.
146. Yu Y, Zhao L, Li R: **Medium-chain acyl-CoA dehydrogenase enhances invasion and metastasis ability of breast cancer cells**. *Nan Fang yi ke da xue xue bao= Journal of Southern Medical University* 2019, **39**(6):650-656.
147. Han S-U, Kim H-T, Seong DH, Kim Y-S, Park Y-S, Bang Y-J, Yang H-K, Kim S-J: **Loss of the Smad3 expression increases susceptibility to tumorigenicity in human gastric cancer**. *Oncogene* 2004, **23**(7):1333-1341.
148. Zhang C, E J, Yu E: **LncRNA CASC21 induces HGH1 to mediate colorectal cancer cell proliferation, migration, EMT and stemness**. *RNA biology* 2021, **18**(sup1):369-381.

149. Zhu Z, Hou Q, Wang B, Li C, Liu L, Gong W, Chai J, Guo H: **A novel mitochondria-related gene signature for controlling colon cancer cell mitochondrial respiration and proliferation**. *Human Cell* 2022, **35**(4):1126-1139.
150. Prokopidis K, Giannos P, Witard OC, Peckham D, Ispoglou T: **Aberrant mitochondrial homeostasis at the crossroad of musculoskeletal ageing and non-small cell lung cancer**. *PloS one* 2022, **17**(9):e0273766.
151. Gao P, Liu Q, Ai B, Fang Y, Wang Z, Wang J: **Prognostic value and clinical significance of LIPH in breast cancer**. *International Journal of General Medicine* 2021:7613-7623.
152. Zhuang H, Chen X, Wang Y, Huang S, Chen B, Zhang C, Hou B: **Identification of LIPH as an unfavorable biomarkers correlated with immune suppression or evasion in pancreatic cancer based on RNA-seq**. *Cancer Immunology, Immunotherapy* 2021:1-12.
153. Kurz S, Thieme R, Amberg R, Groth M, Jahnke H-G, Pieroh P, Horn L-C, Kolb M, Huse K, Platzer M: **The anti-tumorigenic activity of A2M—A lesson from the naked mole-rat**. *PloS one* 2017, **12**(12):e0189514.
154. Zhang G, Liu X, Sun Z, Feng X, Wang H, Hao J, Zhang X: **A2M is a potential core gene in intrahepatic cholangiocarcinoma**. *BMC cancer* 2022, **22**(1):1-12.
155. Liu X, Jin G, Qian J, Yang H, Tang H, Meng X, Li Y: **Digital gene expression profiling analysis and its application in the identification of genes associated with improved response to neoadjuvant chemotherapy in breast cancer**. *World Journal of Surgical Oncology* 2018, **16**(1):1-8.
156. Malvia S, Chintamani C, Sarin R, Dubey US, Saxena S, Bagadi SAR: **ABERRANT EXPRESSION OF COL14A1, CELRS3, and CTHRC1 IN BREAST CANCER CELLS**. 2023.
157. Wang Y, He L, Du Y, Zhu P, Huang G, Luo J, Yan X, Ye B, Li C, Xia P: **The long noncoding RNA lncTCF7 promotes self-renewal of human liver cancer stem cells through activation of Wnt signaling**. *Cell stem cell* 2015, **16**(4):413-425.
158. Jiang Y, Chen F, Ren X, Yang Y, Luo J, Yuan J, Yuan J, Tong Q: **RNA-binding protein COL14A1, TNS1, NUSAP1 and YWHAE are valid biomarkers to predict peritoneal metastasis in gastric cancer**. *Frontiers in Oncology* 2022, **12**:830688.
159. Yoshioka Y, Shimomura M, Saito K, Ishii H, Doki Y, Eguchi H, Nakatsura T, Itoi T, Kuroda M, Mori M: **Circulating cancer-associated extracellular vesicles as early detection and recurrence biomarkers for pancreatic cancer**. *Cancer Science* 2022, **113**(10):3498.
160. Liu J, Shen J-X, Wu H-T, Li X-L, Wen X-F, Du C-W, Zhang G-J: **Collagen 1A1 (COL1A1) promotes metastasis of breast cancer and is a potential therapeutic target**. *Discovery medicine* 2018, **25**(139):211-223.
161. Zhang Z, Wang Y, Zhang J, Zhong J, Yang R: **COL1A1 promotes metastasis in colorectal cancer by regulating the WNT/PCP pathway**. *Molecular Medicine Reports* 2018, **17**(4):5037-5042.
162. Hou L, Lin T, Wang Y, Liu B, Wang M: **Collagen type 1 alpha 1 chain is a novel predictive biomarker of poor progression-free survival and chemoresistance in metastatic lung cancer**. *Journal of Cancer* 2021, **12**(19):5723.
163. Uzozie A, Nanni P, Staiano T, Grossmann J, Barkow-Oesterreicher S, Shay JW, Tiwari A, Buffoli F, Laczko E, Marra G: **Sorbitol dehydrogenase overexpression and other aspects of dysregulated protein expression in human precancerous colorectal neoplasms: a quantitative proteomics study**. *Molecular & Cellular Proteomics* 2014, **13**(5):1198-1218.
164. Lee S-Y, Kim S, Song Y, Kim N, No J, Kim KM, Seo HR: **Sorbitol dehydrogenase induction of cancer cell necroptosis and macrophage polarization in the HCC microenvironment suppresses tumor progression**. *Cancer Letters* 2022, **551**:215960.
165. Zhang H, Han B, Han X, Zhu Y, Liu H, Wang Z, Cui Y, Tian R, Gao Z, Tian R: **Comprehensive analysis of splicing factor and alternative splicing event to construct subtype-specific prognosis-predicting models for breast cancer**. *Frontiers in Genetics* 2021, **12**:736423.
166. Zhao Q, Zhang L, He Q, Chang H, Wang Z, Cao H, Zhou Y, Pan R, Chen Y: **Targeting TRMT5 suppresses hepatocellular carcinoma progression via inhibiting the HIF-1α pathways**. *Journal of Zhejiang University-SCIENCE B* 2023, **24**(1):50-63.
167. Wang Y, Hu L, Zheng Y, Guo L: **HMGA1 in cancer: Cancer classification by location**. *Journal of Cellular and Molecular Medicine* 2019, **23**(4):2293-2302.

168. Yang J, Fan L, Liao X, Cui G, Hu H: **CRTAC1 (Cartilage acidic protein 1) inhibits cell proliferation, migration, invasion and epithelial-mesenchymal transition (EMT) process in bladder cancer by downregulating Yin Yang 1 (YY1) to inactivate the TGF-β pathway**. *Bioengineered* 2021, **12**(2):9377-9389.
169. Long S, Wang J, Weng F, Xiang D, Sun G: **Extracellular matrix protein 1 regulates colorectal cancer cell proliferative, migratory, invasive and epithelial-mesenchymal transition activities through the PI3K/AKT/GSK3β/snail signaling axis**. *Frontiers in Oncology* 2022, **12**:889159.
170. Wang L, Yu J, Ni J, Xu X-M, Wang J, Ning H, Pei X-F, Chen J, Yang S, Underhill CB: **Extracellular matrix protein 1 (ECM1) is over-expressed in malignant epithelial tumors**. *Cancer letters* 2003, **200**(1):57-67.
171. Lee K, Nam K, Oh S, Lim J, Kim R, Shim D, Choi J, Lee S, Yu J, Lee J: **ECM1 regulates tumor metastasis and CSC-like property through stabilization of β-catenin**. *Oncogene* 2015, **34**(50):6055-6065.
172. Zhang X-X, Luo J-H, Wu L-Q: **FN1 overexpression is correlated with unfavorable prognosis and immune infiltrates in breast cancer**. *Frontiers in Genetics* 2022, **13**:913659.
173. Wang H, Zhang J, Li H, Yu H, Chen S, Liu S, Zhang C, He Y: **FN1 is a prognostic biomarker and correlated with immune infiltrates in gastric cancers**. *Frontiers in Oncology* 2022, **12**:918719.
174. Zhou W-H, Du W-D, Li Y-F, Al-Aroomi MA, Yan C, Wang Y, Zhang Z-Y, Liu F-Y, Sun C-F: **The overexpression of fibronectin 1 promotes cancer progression and associated with M2 macrophages polarization in head and neck squamous cell carcinoma patients**. *International Journal of General Medicine* 2022:5027-5042.
175. Bach LA: **Recent insights into the actions of IGFBP-6**. *Journal of cell communication and signaling* 2015, **9**:189-200.
176. Nikulin S, Zakharova G, Poloznikov A, Raigorodskaya M, Wicklein D, Schumacher U, Nersisyan S, Bergquist J, Bakalkin G, Astakhova L: **Effect of the expression of ELOVL5 and IGFBP6 genes on the metastatic potential of breast cancer cells**. *Frontiers in genetics* 2021, **12**:662843.
177. Du X-Y, Liu X, Wang Z-J, Wang Y-Y: **SLPI promotes the gastric cancer growth and metastasis by regulating the expression of P53, Bcl-2 and Caspase-8**. *European Review for Medical & Pharmacological Sciences* 2017, **21**(7).
178. Devoogdt N, Hassanzadeh Ghassabeh G, Zhang J, Brys L, De Baetselier P, Revets H: **Secretory leukocyte protease inhibitor promotes the tumorigenic and metastatic potential of cancer cells**. *Proceedings of the National Academy of Sciences* 2003, **100**(10):5778-5782.
179. Xiao B, Chen L, Ke Y, Hang J, Cao L, Zhang R, Zhang W, Liao Y, Gao Y, Chen J: **Identification of methylation sites and signature genes with prognostic value for luminal breast cancer**. *BMC cancer* 2018, **18**(1):1-13.
180. Dumont V, Lehtonen S: **PACSIN proteins in vivo: Roles in development and physiology**. *Acta Physiologica* 2022, **234**(3):e13783.
181. Zimu Z, Jia Z, Xian F, Rui M, Yuting R, Yuan W, Tianhong W, Mian M, Yinlong L, Enfang S: **Decreased expression of PACSIN1 in brain glioma samples predicts poor prognosis**. *Frontiers in Molecular Biosciences* 2021, **8**:696072.
182. Liu J, Liu Z, Zhang X, Gong T, Yao D: **Bioinformatic exploration of OLFML2B overexpression in gastric cancer base on multiple analyzing tools**. *BMC cancer* 2019, **19**(1):1-10.
183. Hu P, Zhang X, Li Y, Xu L, Qiu H: **Pan-Cancer Analysis of OLFML2B Expression and Its Association With Prognosis and Immune Infiltration**. *Frontiers in Genetics* 2022, **13**:882794.
184. Lin J, Xu X, Li T, Yao J, Yu M, Zhu Y, Sun D: **OLFML2B is a robust prognostic biomarker in bladder cancer through genome-wide screening: A study based on seven cohorts**. *Frontiers in Oncology* 2021, **11**:650678.
185. Liang T, Wu X, Wang L, Ni Z, Fan Y, Wu P, Wang H, Niu Y, Huang H: **Clinical significance and diagnostic value of QPCT, SCEL and TNFRSF12A in papillary thyroid cancer**. *Pathology-Research and Practice* 2023, **245**:154431.
186. Zhang Y, Wang Y, Zhao Z, Peng W, Wang P, Xu X, Zhao C: **Glutaminyl cyclases, the potential targets of cancer and neurodegenerative diseases**. *European Journal of Pharmacology* 2022:175178.

187. Wang H, Li Y, Qi Y, Zhao E, Kong X, Yang C, Yang Q, Zhang C, Liu Y, Song Z: **Pegylated liposomal doxorubicin, docetaxel, and trastuzumab as neoadjuvant treatment for HER2-positive breast cancer patients: A phase II and biomarker study**. *Frontiers in Oncology* 2022, **12**:909426.
188. Che N, Yang Z, Liu X, Li M, Feng Y, Zhang C, Li C, Cui Y, Xuan Y: **Suppression of LETM1 inhibits the proliferation and stemness of colorectal cancer cells through reactive oxygen species–induced autophagy**. *Journal of Cellular and Molecular Medicine* 2021, **25**(4):2110-2120.
189. Zhou B, Yang C, Yan X, Shi Z, Xiao H, Wei X, Jiang N, Wu Z: **LETM1 knockdown promotes autophagy and apoptosis through AMP-activated protein kinase phosphorylation-mediated Beclin-1/Bcl-2 complex dissociation in hepatocellular carcinoma**. *Frontiers in Oncology* 2021, **10**:606790.
190. Natarajan GK, Mishra J, Camara AK, Kwok W-M: **LETM1: a single entity with diverse impact on mitochondrial metabolism and cellular signaling**. *Frontiers in Physiology* 2021, **12**:637852.
191. Sim EUH, Ang CH, Ng CC, Lee CW, Narayanan K: **Differential expression of a subset of ribosomal protein genes in cell lines derived from human nasopharyngeal epithelium**. *Journal of human genetics* 2010, **55**(2):118-120.
192. Park S-M, Choi E-Y, Bae M, Kim S, Park JB, Yoo H, Choi JK, Kim Y-J, Lee S-H, Kim I-H: **Histone variant H3F3A promotes lung cancer cell migration through intronic regulation**. *Nature communications* 2016, **7**(1):12914.
193. Su W, Zhu S, Chen K, Yang H, Tian M, Fu Q, Shi G, Feng S, Ren D, Jin X: **Overexpressed WDR3 induces the activation of Hippo pathway by interacting with GATA4 in pancreatic cancer**. *Journal of Experimental & Clinical Cancer Research* 2021, **40**(1):1-15.
194. Liu W, Xie A, Xiong J, Li S, Yang L, Liu W: **WDR3 promotes stem cell-like properties in prostate cancer by inhibiting USF2-mediated transcription of RASSF1A**. *The Journal of Gene Medicine* 2023:e3498.
195. García-Quispes WA, Pastor S, Galofre P, Biarnes J, Castell J, Velazquez A, Marcos R: **Possible role of the WDR3 gene on genome stability in thyroid cancer patients**. 2012.
